# Population-based body–brain mapping links brain morphology with anthropometrics and body composition

**DOI:** 10.1038/s41398-021-01414-7

**Published:** 2021-05-18

**Authors:** Tiril P. Gurholt, Tobias Kaufmann, Oleksandr Frei, Dag Alnæs, Unn K. Haukvik, Dennis van der Meer, Torgeir Moberget, Kevin S. O’Connell, Olof D. Leinhard, Jennifer Linge, Rozalyn Simon, Olav B. Smeland, Ida E. Sønderby, Adriano Winterton, Nils Eiel Steen, Lars T. Westlye, Ole A. Andreassen

**Affiliations:** 1 Norwegian Centre for Mental Disorders Research (NORMENT), Division of Mental Health and Addiction, Oslo University Hospital and University of Oslo, Oslo, Norway; 2 Department of Psychiatry and Psychotherapy, University of Tübingen, Tübingen, Germany; 3 School of Mental Health and Neuroscience, Faculty of Health, Medicine and Life Sciences, Maastricht University, Maastricht, The Netherlands; 4 Department of Psychology, University of Oslo, Oslo, Norway; 5 AMRA Medical, Linköping, Sweden; 6 Division of Diagnostics and Specialist Medicine, Department of Health, Medicine and Caring Sciences, Linköping University, Linköping, Sweden; 7 Department of Medical Genetics, Oslo University Hospital, Oslo, Norway

**Keywords:** Neuroscience, Biomarkers

## Abstract

Understanding complex body–brain processes and the interplay between adipose tissue and brain health is important for understanding comorbidity between psychiatric and cardiometabolic disorders. We investigated associations between brain structure and anthropometric and body composition measures using brain magnetic resonance imaging (MRI; *n* = 24,728) and body MRI (*n* = 4973) of generally healthy participants in the UK Biobank. We derived regional and global measures of brain morphometry using FreeSurfer and tested their association with (i) anthropometric measures, and (ii) adipose and muscle tissue measured from body MRI. We identified several significant associations with small effect sizes. Anthropometric measures showed negative, nonlinear, associations with cerebellar/cortical gray matter, and brain stem structures, and positive associations with ventricular volumes. Subcortical structures exhibited mixed effect directionality, with strongest positive association for accumbens. Adipose tissue measures, including liver fat and muscle fat infiltration, were negatively associated with cortical/cerebellum structures, while total thigh muscle volume was positively associated with brain stem and accumbens. Regional investigations of cortical area, thickness, and volume indicated widespread and largely negative associations with anthropometric and adipose tissue measures, with an opposite pattern for thigh muscle volume. Self-reported diabetes, hypertension, or hypercholesterolemia were associated with brain structure. The findings provide new insight into physiological body–brain associations suggestive of shared mechanisms between cardiometabolic risk factors and brain health. Whereas the causality needs to be determined, the observed patterns of body–brain relationships provide a foundation for understanding the underlying mechanisms linking psychiatric disorders with obesity and cardiovascular disease, with potential for the development of new prevention strategies.

## Introduction

Obesity is a risk factor for disorders of both the body^1^ and the brain^1,2^, and represents a global health challenge. Although causal mechanisms remain unclear, the associations between brain and physical health likely reflect body–brain interactions and common mechanisms across the soma and the mind. Indeed, patients with psychiatric disorders show subtle structural brain alterations as revealed using brain imaging^3^, and are at increased risk for poor physical health, including obesity, metabolic syndrome, cardiovascular disorders, and shorter life-expectancy^4^. Yet, how these factors relate to brain health remains poorly understood.

Several regions of the brain are recognized to be involved in the complex functions regulating food intake, metabolism, and body composition features^5^. This includes subcortical and cortical structures related to regulation of metabolism, reward system, and higher-level emotional and cognitive functioning^5^. Indeed, cross-sectional brain magnetic resonance imaging (MRI) studies have documented negative associations between obesity/poor metabolic health and gray matter volumes^6–11^ and white matter microstructure^6,7^, while there is conflicting evidence for white matter volumes^6,7^. Regional variations have been reported^6,7,9–11^. Yet, it is not clear how different aspects of obesity, e.g., adipose tissue distribution, or intra-abdominal fat—a known risk factor for adverse health outcomes^1,12,13^—relate to brain health. Nonlinear associations are a common phenomenon in neuroimaging (e.g., accelerating brain atrophy at higher ages^14^), but it is unknown whether aspects of obesity are nonlinearly related to brain structure. The genetic contribution to obesity is substantial and interacts with the environment, lifestyle, and sex^15^. Body mass index (BMI) is associated with genetic loci^16^ and shows genetic overlap with psychiatric disorders^17^. Additionally, body composition, obesity, and brain structure are modifiable and sensitive to environmental and lifestyle factors and sex. Indeed, physical fitness and activity have been positively associated with gray and white matter measures^18,19^, indicating their importance for brain health, while negative associations have been reported for mobility impairment^20^.

Prior studies have largely investigated associations between brain structure and anthropometric measures (e.g., BMI, waist-to-hip ratio (WHR), waist circumference), which are nonspecific measures of body composition. These measures capture slightly different aspects of body composition and each provides independent information relevant for cardiometabolic risk. BMI is a general measure that accounts neither for regional/abdominal adipose tissue nor muscle volume, while both WHR and waist circumference are indicators of abdominal adipose tissue and are better indicators of cardiometabolic risk than BMI (see Tchernof 2013^12^ and Huxley 2010^21^). Regional ectopic fat accumulation appears central for cardiometabolic risk^13^. Visceral adipose tissue (i.e., intra-abdominal fat) have stronger links to cardiometabolic risk than abdominal subcutaneous adipose tissue^12,13^. Ectopic liver fat characterizes non-alcoholic fatty liver disease and is considered a component of metabolic syndrome^22^. Insulin resistance is a risk factor for cardiovascular disease^23^, and the insulin-sensitive skeletal muscle plays an important role in metabolic health;^24^ Ectopic muscle fat infiltration has been associated with obesity^25^ and insulin resistance^24,25^, and muscle mass/strength with insulin sensitivity^26^. Body MRI enables regional specific and detailed in vivo measures of adipose and muscle tissue distribution, allowing for individual body composition profiling with relevance for clinical prediction^27^. How novel body MRI measures relate to individual differences in brain structure is unknown.

The pathophysiology of psychiatric disorders has proven elusive. To disentangle complex and multifactorial mechanisms, with each contributing factor having small effects, we need an improved understanding of normal body–brain connections in healthy individuals. To accurately describe such small effects, and thereby improve our understanding of normative body–brain connections, large-scale investigations are needed^28^.

To this end, we tested for associations between anthropometrics and body composition and brain structure in generally healthy individuals aged 44–82 years using anthropometric measures and brain (*n* = 24,728) and body (*n* = 4973) MRI from the UK Biobank^29^. Based on prior studies^6–10,18^, we expected brain structure to show negative associations with anthropometric and adipose tissue measures, possibly with stronger associations for ectopic fat, and positive associations for muscle volume.

## Methods

### Study design and participants

We included 24,728 generally healthy UK Biobank participants (13,051 women, 11,677 men) with brain MRI and anthropometric measures. A subsample (*n* = 4973) had body composition measures of adipose and muscle tissue from body MRI available. We excluded participants with known diagnosis of cancers, selected traumas, neurological, psychiatric, substance abuse, cardiovascular, liver, or severe infectious conditions (Note S1), with incomplete demographic or clinical data, or who withdrew their informed consent (opt-out-list dated August 20, 2020). We did not exclude based on common metabolic syndrome or lifestyle factors, but adjust for these in the analyses.

UK Biobank has IRB approval from North West Multi-center Research Ethics Committee, its Ethics Advisory Committee (https://www.ukbiobank.ac.uk/ethics) oversees the UK Biobank Ethics & Governance Framework, and informed consent was obtained from all participants^29^. We obtained access to the UK Biobank cohort through Application Number 27412. The study was approved by the Regional Committees for Medical and Health Research Ethics (https://rekportalen.no/) and conducted in accordance with the Helsinki Declaration.

### Demographic and clinical data

We extracted demographic data (age, sex, ethnicity) and variables reflecting cardiovascular risk (including history of diabetes, hypercholesterolemia, hypertension, current cigarette smoker, current alcohol consumption), waist circumference, hip circumference, weight and height, and computed BMI and WHR (Note S2). Tables S1 and S2 summarize the demographic and clinical data of the full sample and the body MRI subsample, respectively.

### MRI acquisition

Participants underwent 3 T brain and 1.5 T body/liver MRI on the same day and site. Brain MRI was available from three sites (Cheadle, Reading, and Newcastle), while body/liver MRI was from one site (Cheadle). Similar scanners/protocols were used across all sites^29^ (Note S3).

### MRI processing

We processed brain MRI DICOM images in-house using FreeSurfer^30^ (version 5.3.0; http://www.freesurfer.net). We extracted mean cortical thickness and white surface area from the cortical parcellation, volumes of cortical/cerebellum gray/white matter, brain stem, CSF, lateral ventricle, third ventricle, thalamus, hippocampus, amygdala, accumbens, caudate, putamen, and pallidum, and computed the average across the hemispheres. We refer to average measures unless otherwise specified. We extracted bilateral measures of cortical area, thickness, and volume for all parcellations of the Desikan-Killiany cortical atlas^31^. Additionally, we extracted Euler numbers^32^ as a proxy of image quality^33^.

We extracted body/liver MRI data processed for visceral adipose tissue (VAT), abdominal subcutaneous adipose tissue (ASAT), liver proton density fat fraction (PDFF), muscle fat infiltration (MFI), and total thigh muscle volume (TTMV), together with an abdominal MRI quality indicator, provided by AMRA (https://www.amramedical.com) and subsequently released by UK Biobank. We computed total abdominal adipose tissue as the sum visceral and subcutaneous adipose tissue (VAT + ASAT) from the available data (Note S4).

### MRI quality control

Among the 42,068 participants with available brain MRI, 27,203 met inclusion criteria. Of these, we excluded two participants that did not have Euler numbers available for automated data quality assessment, prior to iteratively excluding Euler outliers defined as participants with higher negative Euler numbers that exceeded three standard deviations from the mean in either hemisphere. We iterated until no outliers remained, resulting in seven iterations. This led to further exclusion of 2602 participants, yielding a total sample of *n* = 24,728 (Fig. S1; Note S5).

For body MRI, participants labelled with severe motion artifacts, corrupted data, broken coil element, outer field-of-view inhomogeneities, or metal contamination were removed. We further removed 180 participants with incomplete body composition measures, yielding a total body MRI subsample of *n* = 4973 (Fig. S1).

### Statistical analysis

**Fig. 1:**
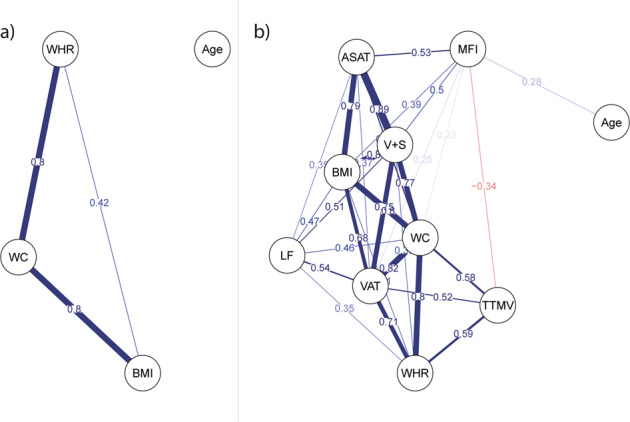
Network correlation graphs of anthropometric and body composition measures and age. The network graphs visualize the structure of the correlation matrices of anthropometric and body composition measures and age; for (**a**) the full sample (*n* = 24,728) and (**b**) the body MRI subsample (*n* = 4973). It was created using the *qgraph*^34^ R function (R version 4.0.0). The edges illustrate the correlation among variables, and the node placement reflects the correlation pattern. Only correlations with r ≥ 0.2 are displayed. ASAT abdominal subcutaneous adipose tissue, BMI body mass index, LF liver proton density fat fraction, MFI muscle fat infiltration, TTMV total thigh muscle volume, VAT visceral adipose tissue, V + S total abdominal adipose tissue (VAT + ASAT), WC waist circumference, WHR waist-to-hip ratio.

We investigated the sample demographics across and within sexes. Categorical variables were compared using χ^2^-test. For normally/non-normally distributed continuous variables we used the two-sample *t*-test/Wilcoxon rank-sum test. For unequal variance across sexes, *t*-test was replaced by Welch approximation. Normality was assessed by visual evaluation of density plots (Figs. S2–S3). We derived correlation matrices of anthropometric and body composition measures and age, and evaluated the corresponding network graphs^34^ (Fig. 1). For brain structure, we assessed density plots for expected distribution patterns (Figs. S4–S5; not shown for cortical parcellations), and scatter plots of body–brain associations (Figs. S6–S14; not performed for cortical parcellations).

For descriptive purposes we initially assessed age- and sex-related associations on anthropometric and body composition measures and brain structure using a three-step multiple linear regression approach: model 1a included age, age^2^, and sex; model 1b additionally included age-by-sex and age^2^-by-sex interactions; and model 1c additionally included metabolic/lifestyle variables, including ethnicity (due to differences in adipose tissue distribution/accumulation^12^), current cigarette smoking (yes/no), current alcohol consumption (yes/no), diabetes (yes/no), hypertension (yes/no), and hypercholesterolemia (yes/no). Model 1c was only applied to anthropometric and body composition measures.

In the main analyses, building upon model 1b/c, we tested for linear and quadratic associations between brain structure (dependent variable) and anthropometric and body composition measures. We used a three-step multiple linear regression approach: model 2a included a linear body measure term; model 2b additionally included a quadratic body measure term; and model 2c additionally included metabolic/lifestyle variables. Model 2a/b extends model 1b, while model 2c extends model 1c. Additionally, for model 2c only, we conducted: (i) follow-up analyses for laterality effects; and (ii) regional analyses of cortical area, thickness, and volume.

We used regression models of incremental complexity to motivate the fully adjusted models, and to explore the importance of nonlinearities in the observed body–brain associations. Separate analyses were conducted for the full sample and body MRI subsample (when applicable). For brain MRI, we additionally adjusted for intracranial volume (except for cortical thickness), image quality (average Euler number), and site (when applicable). Categorical variables were included as factors, continuous variables were mean-centered.

We evaluated model residuals for normality using residual vs fitted value and Q–Q plots, leading to log-transformation of dependent variables for models showing a significant (and consistent) departure from normality, namely: all outcomes for sample description analyses of anthropometric and body composition and CSF, lateral, and third ventricle volumes for brain MRI analyses (Figs. S15–S20 presents selected illustrations). Remaining dependent variables were not log-transformed.

All statistical analyses were conducted in R (version 3.6.0; https://www.r-project.org). We used *lm* for the regression analyses (Note S6), and computed the partial correlation coefficients, *r*, effect size directly from the *t*-statistics for continuous variables and via Cohen’s d for categorical variables^35^. We used Bonferroni correction to adjust for multiple comparison at α = 0.05 across N_1_ = (3 + 9)(1 + 17)+17 = 233 independent tests, which is the number of regression models from sample description (i.e., n = 3 + 9 + 17 tests) and main analyses (i.e., n = (3 + 9)×17 tests) for both the full sample and body MRI subsample, counting partly overlapping models once, reflecting the included 17 brain structures, 3 anthropometric, and 6 body composition measures (i.e., 3 + 6 measures in body MRI subsample). This resulted in a study-wide significance threshold of *p* ≤ α/N_1_ = 0.0002. Similarly, we computed a separate Bonferroni threshold for the regional cortical analyses across N_2_ = 34×3(3 + 9)=1224 independent tests, which is the number of cortical parcellations, cortical measures (i.e., area, thickness, and volume), and anthropometric and body composition measures from the full sample and body MRI subsample, yielding a significance threshold of p ≤ α/N_2_ = 4.1e-05. We present the overall global picture of significant findings from model 2c, and the range of *p*-values and *r* effect sizes (absolute values; indicated by |*r* | ). The full results are presented in the supplemental material.

## Results

### Demographic variables

The full sample (*n* = 24,728) included more women (*n* = 13,051; 52.8%) than men (*n* = 11,677; 47.2%), and was largely of self-identified European ancestry (96.5%). The age range was 44–82 years. Compared to women, men had significantly higher age, more alcohol consumers and cigarette smokers, higher anthropometric measures (except hip circumference), and more were diagnosed with diabetes, hypercholesterolemia, and hypertension (Table S1; Fig. S2). Similarly, the body MRI subsample (*n* = 4973) included more women (*n* = 2652; 53.3%) than men (*n* = 2321; 46.7%), the age range was 44–79 years, and men had higher age and generally higher levels of adverse factors than women. For body composition measures, men had higher liver PDFF, VAT, and TTMV, but lower MFI and ASAT than women (Table S2; Fig. S3). We derived network correlation graphs of anthropometric and body composition measures and age (Fig. 1). Both liver PDFF and MFI showed weaker ties to the other adipose tissue measures, while TTMV showed links to measures of abdominal adipose tissue.

Primarily for descriptive purposes, the sample characteristics were further explored for age- and sex-associations on anthropometric and body composition measures and brain structure (Note S7; Tables S3–S5).

### Brain structure and anthropometric measures

**Fig. 2:**
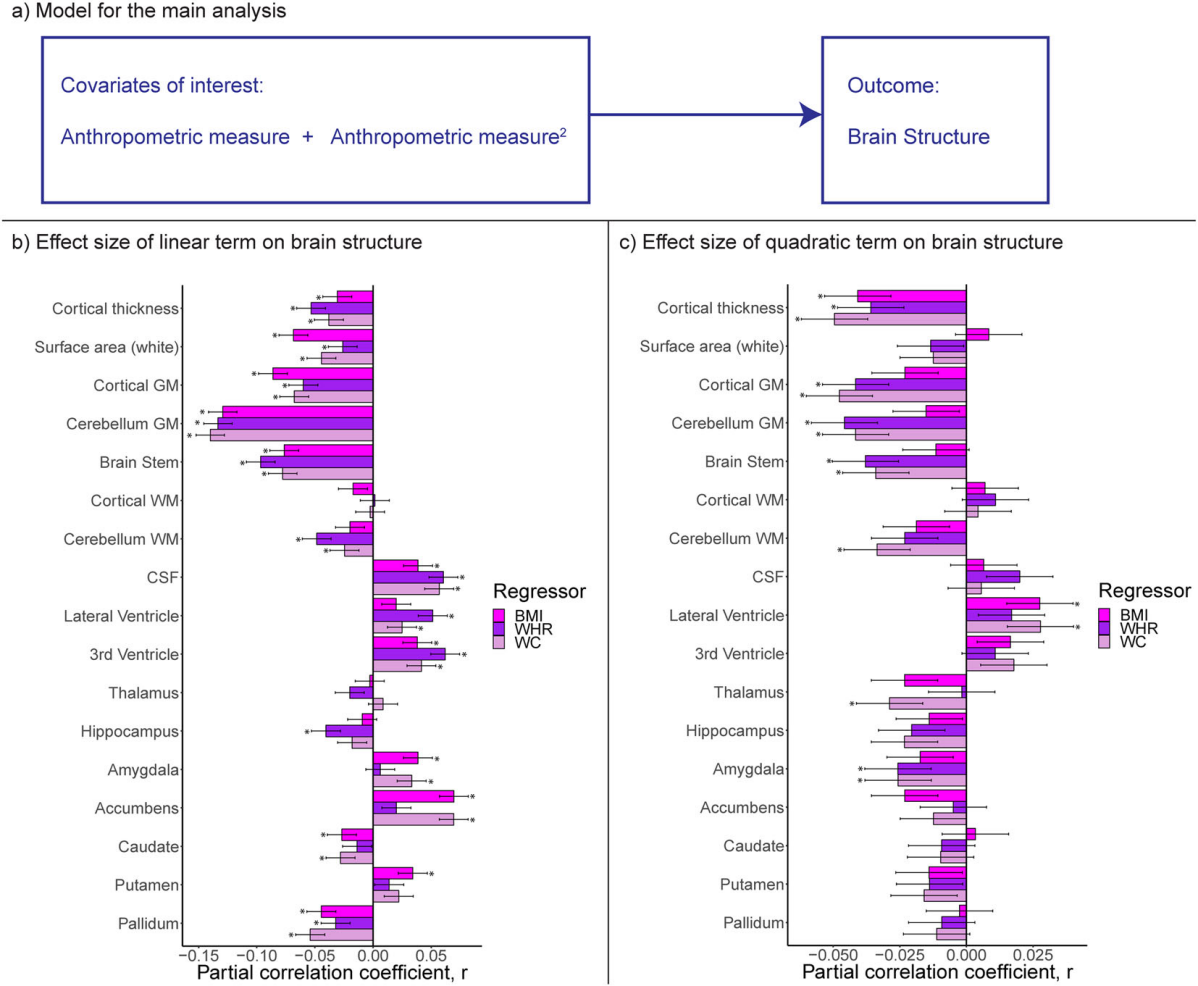
Body–brain association patterns for anthropometric measures (*n* = 24,728). Results from model 2c (**a**) that investigates body–brain connections through the inclusion of linear (**b**) and quadratic (**c**) terms of anthropometric measures. The regression model was adjusted for age, age^2^, sex, age-by-sex, age^2^-by-sex, intracranial volume (except cortical thickness), lifestyle/metabolic factors, Euler number, and site. Significant associations indicated by asterisk. Dependent variables CSF, lateral/3rd ventricle were log-transformed. BMI body mass index, GM gray matter, WC waist circumference, WHR waist-to-hip ratio, WM white matter.

Analyses in the full sample revealed overall negative and nonlinear associations between anthropometric measures and global brain volumes, positive associations for ventricular volumes, and a mixed picture for subcortical structures (Figs. S6–S8). Using model 2c, which investigates both linear and quadratic terms of anthropometric measures on brain structure (Fig. 2a), we observed the largest negative effect sizes of the linear term for cerebellum/cortical gray matter and brain stem volumes, mean surface area, and mean cortical thickness (Fig. 2b), together with significant quadratic terms suggestive of negative and accelerating reductions for cerebellum/cortical gray matter and brain stem volumes, and mean cortical thickness (Fig. 2c) at higher anthropometric measures (Tables S6–S8). Further, we observed smaller cerebellum white matter and indications of accelerating reduction with higher anthropometric measures. There were positive associations between anthropometric measures and CSF, third, and lateral ventricle. Lateral ventricle also displayed positive quadratic associations suggestive of accelerated expansion. Subcortical structures exhibited mixed results. Higher anthropometric measures were negatively associated with pallidum, caudate, and hippocampus volumes, and positively associated with amygdala, accumbens, and putamen volumes. There were significant negative quadratic terms for thalamus, and amygdala volumes, suggestive of accelerating reduction for thalamus, while amygdala increase was quadratically attenuated. In general, both waist circumference and WHR showed a stronger negative, nonlinear, component than BMI. Effect sizes of the quadratic terms were small (|*r* | ≤0.05). The associations were similar bilaterally (Fig. S21; Table S9).

The results were robust across models 2a/b/c, with some adjustment of significant levels and effect sizes, with *p* in [7.8 × 10^−118^, 0.0002], and |*r* | in [0.02, 0.15] (Fig. S22; Tables S6–S8). For model 2c, compared to models 2a/b, we observed attenuation of significance levels and effect sizes with *p* in [2.3 × 10^−108^, 0.0002] and |*r* | in [0.02, 0.14] between body and brain anthropometrics. Additionally, we here observed significant associations (|*r* | in [0.02, 0.06]) between self-reported diabetes, hypertension or hypercholesterolemia, and brain structure, including smaller cortical/cerebellum gray matter and brain stem volumes, and larger ventricles. Hypercholesterolemia showed significant association only with brain stem volume. Current cigarette smoking was negatively associated with mean cortical thickness, while current alcohol consumption was not significantly associated with brain structure.

**Fig. 3:**
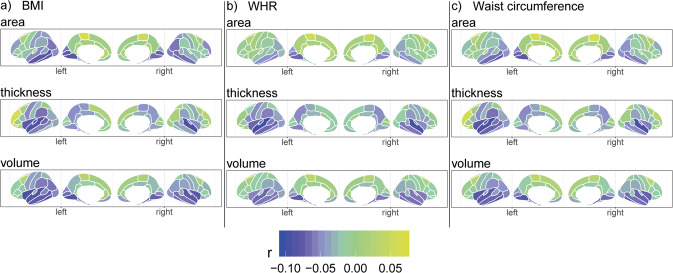
Linear body–brain association patterns across cortical parcellations for anthropometric measures (*n* = 24,728). Results from model 2c that investigates body–brain connections through the inclusion of linear and quadratic terms of anthropometric measures, here showing the results of the linear term for (**a**) BMI, (**b**) WHR, and (**c**) waist circumference. The regression model was adjusted for age, age^2^, sex, age-by-sex, age^2^-by-sex, intracranial volume (except cortical thickness), lifestyle/metabolic factors, Euler number, and site. Figure was created using the *ggseg*^85^ R function. BMI body mass index, r partial correlation coefficient, WHR waist-to-hip ratio.

Regional analyses showed a number of significant, predominantly negative, linear associations between anthropometric measures and cortical area, thickness, and volume (Fig. 3; Tables S10–S12; model 2c), together with some indications of nonlinearities in the association (|*r* | ≤0.06) particularly for thickness (Fig. S23). The significance levels and effect sizes were in range *p* in [1.0 × 10^−59^, 4.1 × 10^−05^] and |*r* | in [0.03, 0.10]. These regions were predominantly in the temporal/occipital lobes and insula, but were also observed in the frontal/parietal lobes. We observed peak negative effect sizes for (i) area of inferior temporal (temporal lobe) and lingual and pericalcarine (occipital lobe) regions; (ii) thickness of the superior temporal region (temporal lobe) and insula; and (iii) volume of the entorhinal, fusiform, superior temporal regions (temporal lobe), and insula. Positive associations were observed, and were most pronounced for area of the paracentral region (frontal lobe) and thickness of the rostral middle frontal region. The association patterns were similar—although not identical—across hemispheres and anthropometric measures. Regional associations with self-reported diabetes, hypertension, and/or smoking were observed (Tables S10–S12).

### Brain structure, anthropometric, and body composition measures

**Fig. 4:**
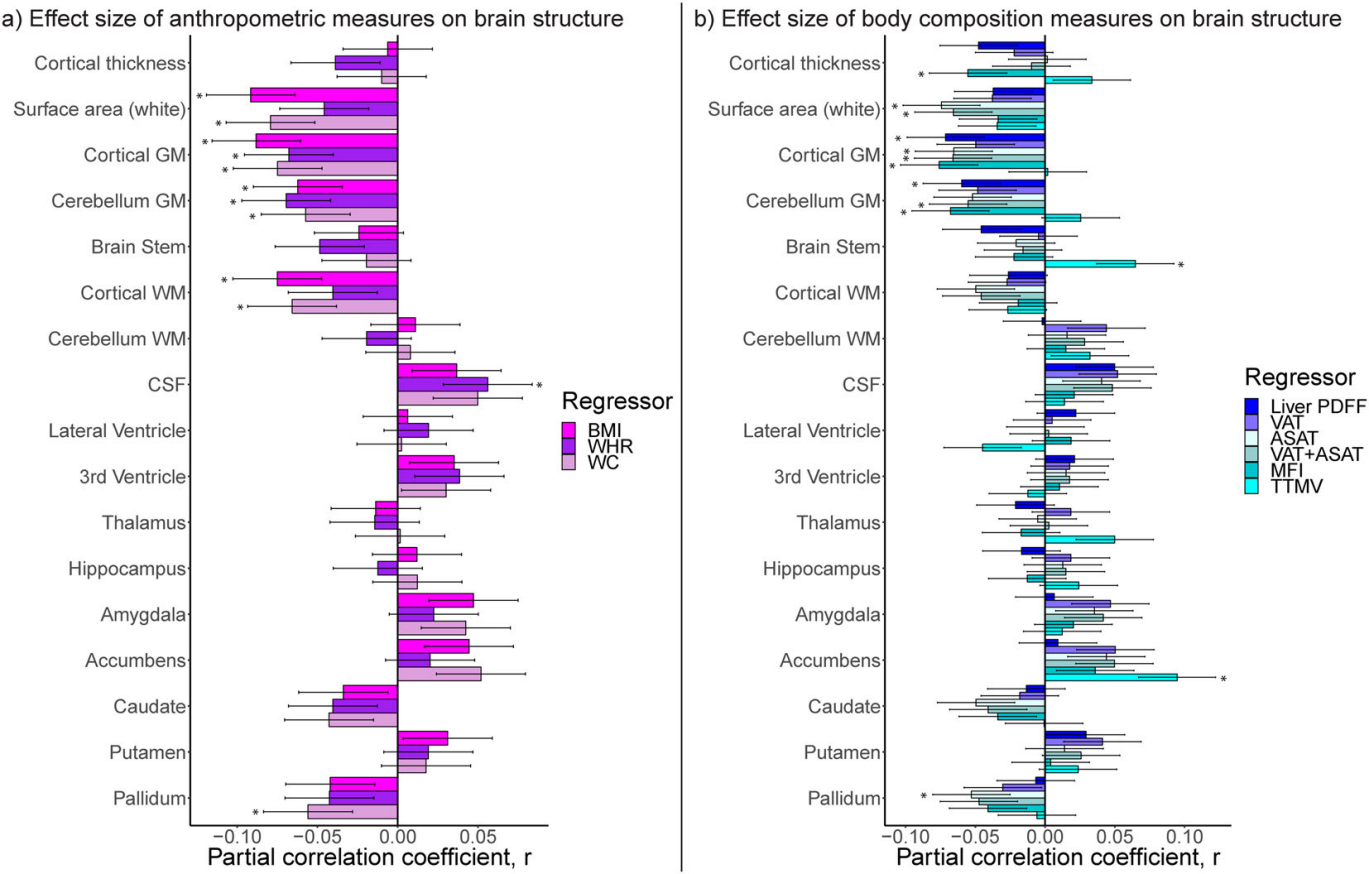
Linear body–brain association patterns for anthropometric and body composition measures (*n* = 4973). Results from model 2c that investigates body–brain connections through the inclusion of linear and quadratic terms of anthropometric and body composition measures, here showing the results for the linear term for in (**a**) anthropometric measures, and in (**b**) body composition measures, on brain structure. The regression model was adjusted for age, age^2^, sex, age-by-sex, age^2^-by-sex, intracranial volume (except cortical thickness), lifestyle/metabolic factors, Euler number, and site. Significant associations indicated by asterisk. Dependent variables CSF, lateral/3rd ventricle were log-transformed. ASAT abdominal subcutaneous adipose tissue, BMI body mass index, GM gray matter, MFI Muscle Fat Infiltration, PDFF proton density fat fraction, TTMV total thigh muscle volume, VAT visceral adipose tissue, WC waist circumference, WHR waist-to-hip ratio, WM white matter.

Analyses in the body MRI subsample revealed similar patterns of brain structure associations for anthropometric and adipose tissue measures (Fig. 4). For anthropometric measures (Fig. 4a), there were significant negative associations with mean surface area (except WHR) and cortical/cerebellum gray matter volume. BMI/waist circumference were negatively associated with cortical white matter, which was not observed in the full sample. WHR was positively associated with CSF, and waist circumference negatively associated with pallidum volume. For adipose tissue measures (Fig. 4b), liver PDFF was negatively associated with cortical/cerebellum gray matter volumes. ASAT was negatively associated with mean surface area, cortical gray matter, and pallidum volumes, while VAT + ASAT was negatively associated with mean surface area and cortical/cerebellum gray matter volumes. MFI was negatively associated with mean cortical thickness and cortical/cerebellum gray matter volumes. TTMV was positively associated with brain stem and accumbens volumes. The associations were similar across models 2a/b/c, with anthropometric measures showing significant *p* in [1.3 × 10^−12^, 0.0002], effect sizes |*r* | in [0.05, 0.1], and body composition measures showing significant *p* in [1.8 × 10^−11^, 0.0002] and |*r* | in [0.05, 0.1]. There were fewer significant findings when using the fully adjusted model 2c compared to models 2a/b (Figs. S24–S25; Tables S13–S21). The associations were similar bilaterally (Figs. S26–S27; Table S22).

**Fig. 5:**
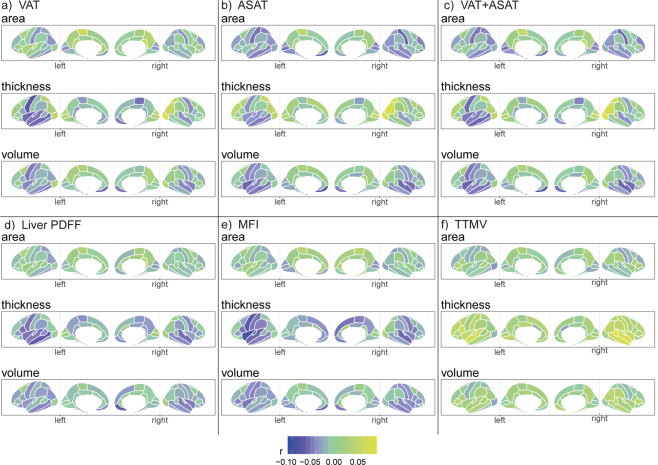
Linear body–brain association patterns across cortical parcellations for body composition measures (*n* = 4973). Results from model 2c that investigates body–brain connections through the inclusion of linear and quadratic body composition terms, here showing the results of the linear term for (**a**) VAT, (**b**) ASAT, (**c**) VAT + ASAT, (**d**) Liver PDFF, (**e**) MFI, and (**f**) TTMV. The regression model was adjusted for age, age^2^, sex, age-by-sex, age^2^-by-sex, intracranial volume (except cortical thickness), lifestyle/metabolic factors, Euler number, and site. Figure created using the *ggseg*^85^ R function. ASAT abdominal subcutaneous adipose tissue, MFI Muscle Fat Infiltration, PDFF proton density fat fraction, TTMV total thigh muscle volume, VAT visceral adipose tissue.

Regional analyses of anthropometric and body composition measures (Figs. 5, S28–S29; Tables S23–S31) revealed fewer significant findings for anthropometric measures in the body MRI subsample (Fig. S28; Tables S23–S25) than in the full sample (Figs. 3, S23; Tables S10–S12), and the overall association patterns differed somewhat (Figs. 3, S28). There were, however, several similarities across anthropometric and adipose tissue measures. We observed similar, negative, association patterns particularly for (i) area of entorhinal and fusiform (temporal lobe), medial orbitofrontal (frontal lobe), and postcentral (parietal lobe) regions; (ii) thickness of the middle/superior temporal (temporal lobe), precentral (frontal lobe) regions, and insula; and (iii) volume of the entorhinal, fusiform, superior temporal (temporal lobe), medial orbitofrontal (frontal lobe) regions, and insula. Measures of ectopic fat (VAT, MFI, and Liver PDFF) showed no significant associations with area. Peak positive effect sizes were observed for thickness of the lateral occipital region (occipital lobe) for VAT, ASAT, VAT + ASAT, BMI, and waist circumference, but not the other measures. For TTMV we observed an overall positive pattern with peak effect sizes for thickness of the banks of the superior temporal sulcus/superior temporal (temporal lobe) regions. Anthropometric measures had significant *p* in [4.8 × 10^−12^, 4.1 × 10^−05^] and effect sizes |*r* | in [0.06, 0.10], while body composition measures had *p* in [1.05 × 10^−10^, 4.1 × 10^−05^] and |*r* | in [0.06, 0.09]. There were indications of some nonlinearities (|*r* | ≤0.07).

## Discussion

In the largest study of body–brain relationships to date, we mapped connections between brain structures and anthropometric measures (i.e., BMI, WHR, and waist circumference) in 24,728 generally healthy participants without secondary disease effects. Further, we examined novel body MRI measures of adipose and muscle tissue in a subsample (*n* = 4973). For global brain measures, the observed effect sizes were strongest between anthropometric and body composition measures and cortical/cerebellum and brain stem structures. Widespread regional association patterns were observed across the cortex for both anthropometric and body composition measures, with opposing patterns between measures of adipose and muscle tissue. Together, these findings revealed a picture of distributed small, highly significant, associations between anthropometrics and body composition and the majority of brain structures. This comprehensive map of widespread body–brain relationships can be used to delineate their role in human health.

For global brain measures, we observed negative and nonlinear associations between anthropometric measures and cerebellar/cortical gray matter, brain stem structures, and positive associations with ventricles. Across the cortex, widespread regional associations were observed with peak negative effect sizes in temporal and occipital lobe and insula. These findings are in line with our hypothesis and prior research showing smaller gray matter volume^6–9^, and regional cortical^10,11^ and cerebellar reductions^10^ in obese individuals. These structures are also frequently implicated in brain disorders^3^. Brain atrophy is observed at higher ages^14^. Although cross-sectional evidence and small effect sizes, our findings suggest accelerating brain atrophy at higher levels of adipose tissue, possibly relating to regulatory differences in brain and body lipids. We observed the strongest nonlinear effect sizes for waist circumference, which may imply accelerated reductions particularly at higher abdominal adipose tissue. Prior studies on subcortical structures and anthropometrics are limited. Here, we showed a mixed subcortical association pattern, that were similar across anthropometric measures. Accumbens, a structure associated with motivation and reward, and part of the dopamine motivation system^36^, showed the strongest positive associations. This is generally in line with prior studies documenting larger accumbens volume in children with increased genetic risk for obesity^37^, and in predominantly adult obesity across patients with major depressive disorders and healthy controls^11^, and increased accumbens cell density in obese children^38^, and supports the assumption of a critical role of brain mechanisms for reward and reinforcement learning for lifestyle and dietary choices and obesity.

The association patterns were similar for anthropometric and adipose tissue measures, showing smaller global measures of cortical/cerebellum structures for most measures. For cortical thickness/volume, we observed patterns of peak negative effect sizes in temporal, insula, and precentral regions that appear fairly general. This may suggest that general mechanisms related to adipose tissue accumulation also affect cortical structures. Notably, cortical area did not show any significant associations with measures of ectopic fat, but did show significant associations with the other anthropometric and adipose tissue measures. We might speculate, in light of the genetic contribution to surface area^39^ and abdominal adipose tissue^40,41^, that common genetic factors are involved. However, this may also reflect links between adipose tissue and lifestyle-related metabolic risk factors^13,41^. Our observation of an opposing pattern for muscle volume is in line with literature suggesting a link between fitness and brain structure^18,19^, and between muscular strength and brain health^42^. Further investigations in intervention studies are needed.

Our findings point towards ongoing biological processes related to adipose tissue may also affect brain structures, or vice versa, in generally healthy individuals. Obesity is prevalent in psychiatric disorders^4^, and adipose tissue may contribute to both psychiatric and somatic outcomes. Indeed, non-alcoholic fatty liver disease is linked with metabolic factors, shows increasing prevalence, and increased mortality from cardiovascular disease^43^. Studies indicate links between non-alcoholic fatty liver disease and sarcopenia^44,45^—a muscle disease characterized by low muscular strength and muscle fat infiltration^46^. Both non-alcoholic fatty liver disease and sarcopenia are linked to both metabolic factors^45^ and brain-related traits including cognition^47–50^ and mood^47–49,51^. Further, non-alcoholic fatty liver disease has together with type 2 diabetes been associated with brain structure alterations^52,53^. This suggests that understanding the complex mechanisms relating regional adipose tissue with brain-related traits may be important for understanding the interplay between cardiometabolic and mental disorders.

It is also of interest that the association patterns were similar for regional adipose tissue measures versus general anthropometric measures. Further, the association patterns appeared more focused across cortical parcellations in the full sample, demonstrating the need for large samples when investigating complex human phenotypes driven by multiple small effects. Contrary to our expectations, we did not observe stronger associations between ectopic fat and brain structures. Instead, we observed a largely analogous pattern across anthropometric and adipose tissue measures. Effect sizes were generally stronger in the full sample and for anthropometric measures, which may reflect the need for large samples when dealing with small effects and the combinatorial effects of multiple factors that likely influence anthropometric measures. Still, the body composition measures from body MRI may capture more specific features, but were available for only ~20% of the sample.

Earlier studies have linked vascular risk factors to brain structure^6,9^. Our study further corroborates this in generally healthy individuals, and indicates an association between adipose tissue distribution and brain structure. Effect sizes and significance levels were attenuated when we adjusted for lifestyle and metabolic factors, suggestive of complex body–brain mechanisms. Self-reported diagnosis of hypercholesterolemia, hypertension, or diabetes—factors related to metabolic health—were associated with several brain structures. In line with prior research^54^, current cigarette smoking was associated with thinner cortex. Thus, cardiometabolic risk factors appear important for brain health, and may underlie the cardiometabolic comorbidity in psychiatric and other brain disorders.

The observed body–brain connections cut across several anthropometric and body compositions measures and brain structures, and appeared fairly global. Causal mechanisms are unknown and likely highly complex and multifactorial. Our findings of a negative link between adipose tissue and brain structure could be mediated by modifiable lifestyle choices with known links to obesity^1^; e.g., metabolic factors could influence both somatic and brain health, impaired brain health could influence somatic health, or the effects could be reciprocal as previously implied for obesity and depression^2^. Further, physical fitness and activity are associated with reduced risk for obesity^1^, may counteract a genetic predisposition for obesity^15^, have neuroprotective effects^55^, and have been positively related to brain structure^18,19^—as also corroborated by this study. This further support the notion that lifestyle interventions for promoting metabolic health may be important for brain health^56,57^. Although it is premature to conclude, from a public health perspective, this may imply that lifestyle interventions for normalizing adipose tissue composition and promoting physical activity may affect biological processes related to brain function and disease.

The high degree of somatic comorbidity in psychiatric and other brain disorders requires a better understanding of the complex biological body–brain interactions, and of how they relate to lifestyle or environmental factors. The observed body–brain connections could be associated with obesity-related neuroinflammatory processes^58^. A recent large-scale meta-analysis showed increased risk for vascular dementia—similar to other vascular conditions—in both underweight and obese individuals^59^, which is interesting in light of our observed nonlinear associations between anthropometrics and body composition and brain structure. Shared genetic underpinnings may influence both adipose tissue accumulation and brain structure, similar to the genetic overlap relating immune-related disorders^60^, BMI^17^, or cardiovascular risk factors^61^, to brain disorders. Circuits that link cerebral cortex and muscle tissue and adrenal medulla have been identified in monkeys, which may imply that cortical regions influence the functioning of internal organs and relate to somatic comorbidities of mental states^62^. Yet other complex biological and possibly polygenic processes, lifestyle/environmental factors, and/or their combinatorial effects could influence the findings. To understand both nature and nurture of somatic comorbidities in psychiatric disorders, and brain disorders per se, further mechanistic investigations are warranted. The findings of this study may suggest that anthropometrics and body composition are important confounding factors that should be considered in future case-control studies.

The findings of the present study suggests multiple small effects, in line with previous reports on the relationship between BMI and brain structure^9,11^. A similar pattern of small and distributed effects are also observed across a series of studies relating sociodemographic and lifestyle factors^63–66^, and mental disorders^3,67–79^, to brain structures, as well as studies of neuroimaging genetics^3,28,39,80^. This is in line with the concept that small effects are the new normal in brain imaging research^28,80^, and of small polygenic effects and genetic pleiotropy across psychiatric disorders^81^, making single underlying causal mechanisms unlikely^80^. Combinatorial complex mechanisms of additive small effects are more likely, and these can only be robustly captured by large studies with adequate power to identify true effects with stable estimates of effect sizes^28^. Thus, large-scale investigations are needed, where effect size convergence and increasing accuracy is obtained^28^, but this is challenging to achieve. To our knowledge, this is the first study of its magnitude investigating anthropometrics, body composition, and brain structure in largely healthy individuals. Through such large-scale investigations we may better understand small, but normal, body–brain processes. This will provide us with a better understanding of putative interactive or confounding factors in psychiatric and other brain disorders, thereby enhancing our conceptual understanding of the complex mechanisms at play.

This study had some limitations. Data from the UK Biobank are released in bulks, and we may only work with the data we have access to. At the time of MRI, the available diagnostic information was self-reported and we did not have available serum measures. We neither investigate cumulative or unit effect of alcohol consumption or cigarette smoking, nor severity of hypertension, hypercholesterolemia, or diabetes diagnosis. The observed liver–brain associations could be influenced by alcohol consumption although this was not captured by the current study. The subsample with available body MRI may have been underpowered to detect small body–brain associations in generally healthy individuals. We limited our investigations to coarser brain measures, and vertex-wise analyses might provide a more refined picture. Findings deviated somewhat from prior studies using partly overlapping samples^8,9,82^, probably due to differences in inclusion/exclusion criteria, sample size, image processing, and analyses pipeline. The exploratory cross-sectional design makes it difficult to disentangle cause from effect, determine underlying body–brain mechanisms, and draw final conclusions. For the statistical analyses, we used multiple linear regression, and we did not investigate multivariate association patterns of the body–brain-link. Further investigations of multivariate and possibly sex- or age-specific body–brain patterns of anthropometrics, body composition, metabolic markers, and brain structure, together with links to cognition, brain disorders, lifestyle, and genotype are needed. Additionally, UK Biobank participants are healthier than the general UK population^83^, likely reflecting that ability and willingness to participate in medical research is not randomly distributed in the population. For MRI studies, physical constraints of the MRI scanner (e.g., difficult to scan severely obese participants), contraindicators of MRI, and participant removal during quality assessment (e.g., due to motion) may further bias the sample. Future investigations of how the MRI subsample compares to the full UK Biobank sample would be interesting.

Strengths of the study include an unprecedented sample size, more than double that of prior UK Biobank studies^8,9,82^, that were assessed using standardized procedures and MRI protocols^29^. Fully automated data cleaning, inclusion and exclusion criteria, and quality control limits chance for subjective variations or errors. We build upon confirmatory analyses of known age- and sex-related associations on both anthropometrics and body composition^12^ and brain structure^14,84^ that largely mirrored the current knowledge, which strengthened our confidence in the reported body–brain patterns. We applied rigorous diagnosis-based exclusion criteria to capture normative body–brain associations in generally healthy individuals, and rigorous correction for multiple comparisons.

## Conclusions

Through large-scale body–brain mapping we link normally varying anthropometric and body composition measures to brain structure in a largely healthy population. The results imply correlated associations between higher adipose tissue and poor metabolic health and brain structure, affecting global brain structures, brain cavities, and accumbens. While the causal mechanism remain unknown, it is of vital importance to investigate the underlying complex body–brain pathways, shared mechanisms of cardiometabolic risk factors and brain disorders, and lifestyle-related modifying factors. If brain structure alterations can be linked to lifestyle-related anthropometric and body composition characteristics, then preventive public health interventions for normalizing cardiometabolic risk factors could prevent the development of disorders of the body and the brain.

## Supplementary information

Supplemental Material

Supplemental Material Tables

## Data Availability

The UK Biobank resource is open for eligible researchers upon application (http://www.ukbiobank.ac.uk/register-apply/).

## Code availability

We made use of publicly available resources for processing the image data and for conducting the statistical analyses. The project R-scripts are available at https://github.com/comorment/BodyBrainImaging.
