# Supplementary material for: Population-based body–brain mapping links brain morphology with anthropometrics and body composition
Source: Transl Psychiatry. 2021 May 18;11:295. doi: 10.1038/s41398-021-01414-7 (PMC8131380; doi:10.1038/s41398-021-01414-7)
Supplement: Supplementary file 1 — Supplemental Material [file 41398_2021_1414_MOESM1_ESM.pdf]

Supplementary Information

Table of contents

SUPPLEMENTAL FIGURES 4

Figure S1: Inclusion/exclusion pipeline, including overview of automatic quality control step. 4

Figure S2: Distribution continuous demographic variables for the whole sample (n=24,728). 5

Figure S3: Distribution of continuous demographic variables for the body MRI subsample (n=4,973). 6

Figure S4: Distribution of included brain structures for the whole sample (n=24,728). 7

Figure S5: Distribution of included brain structures for the body MRI subsample (n=4,973). 8

Figure S6: Scatter plots of brain structures with BMI (n=24,728). 9

Figure S7: Scatter plots of brain structures with WHR (n=24,728). 10

Figure S8: Scatter plots of brain structures with waist circumference (n=24,728). 11

Figure S9: Scatter plots of brain structures with liver PDFF (n=4,973). 12

Figure S10: Scatter plots of brain structures with VAT (n=4,973). 13

Figure S11: Scatter plots of brain structures with ASAT (n=4,973). 14

Figure S12: Scatter plots of brain structures with VAT+ASAT (n=4,973). 15

Figure S13: Scatter plots of brain structures with MFI (n=4,973). 16

Figure S14: Scatter plots of brain structures with TTMV (n=4,973). 17

Figure S15: Residual versus fitted value plots and Q-Q plots for models with anthropometric measures as dependent variable, with (left) and without (right) log-transformation of dependent variables (full sample; n=24,728). 18

Figure S16: Residual versus fitted value plots and Q-Q plots for models with models with anthropometric and body composition measures as dependent variable: with (left) and without (right) log-transformation of dependent variables (body MRI subsample; n=4,973). 19

Figure S17: Residual versus fitted value plots and Q-Q plots for models investigating brain structures as dependent variable after log-transformation of CSF, lateral ventricle, and 3rd ventricle (full sample; n=24,728). 20

Figure S18: Residual versus fitted value plots and Q-Q plots for CSF, lateral ventricle, and 3rd ventricle as the respective dependent variables: with (left) and without (right) log-transformation (full sample; n=24,728). 21

Figure S19: Residual versus fitted value plots and Q-Q plots for models investigating brain structures as dependent variable after log-transformation of CSF, lateral ventricle, and 3rd ventricle (body MRI subsample; n=4,973). 22

Figure S20: Evaluation of multiple linear regression model residuals for normality using residual versus fitted value plots and Q-Q plots for CSF, lateral ventricle, and 3rd ventricle as the respective dependent variables: with (left) and without (right) log-transformation (body MRI subsample; n=4,973). 23

Figure S21: Body-brain associations in healthy for bilateral measures of brain structure (n=24,728). 24

Figure S22-a: Linear body-brain associations in healthy across models 2a/b/c (n=24,728). 25

Figure S22-b: Quadratic body-brain associations in healthy across models 2b/c (n=24,728). 26

Figure S23: Quadratic body-brain association patterns across cortical parcellations in healthy individuals (n=24,728). 27

Figure S24-a: Linear body-brain associations in healthy across models 2a/b/c (n=4,973) for anthropometric measures. 28

Figure S24-b: Quadratic body-brain associations in healthy across models 2b/c (n=4,973) for anthropometric measures. 29

Figure S25-a: Linear body-brain associations in healthy across models 2a/b/c (n=4,973) for body composition measures. 30

Figure S25-b: Quadratic body-brain associations in healthy across models 2b/c (n=4,973) for body composition measures. 31

Figure S26-a: Bilateral effects for anthropometric measures on brain structure in body MRI subsample (n=4,973) – Left hemisphere. 32

Figure S26-b: Bilateral effects for anthropometric measures on brain structure in body MRI subsample (n=4,973) – Right hemisphere. 33

Figure S27-a: Bilateral effects for body composition measures on brain structure in body MRI subsample (n=4,973) – Left hemisphere. 34

Figure S27-b: Bilateral effects for body composition measures on brain structure in body MRI subsample (n=4,973) – Right hemisphere. 35

Figure S28: Linear and quadratic body-brain association patterns across cortical parcellations for anthropometric measures in the body MRI subsample (n=4,973). 36

Figure S29: Quadratic body-brain association patterns across cortical parcellations for body composition measures (n=4,973). 37

SUPPLEMENTAL TABLES 38

Table S1: Demographics for the full sample (n=24,728) 38

Table S2: Demographics for the body MRI subsample (n=4,973). 39

Tables S2-S31 are presented sheet-wise in a separate supplemental excel document 40

SUPPLEMENTAL NOTES 42

Note S1: Exclusion criteria for the study 42

Note S2: Extracted/computed demographic and clinical variables 44

Note S3: MRI acquisition **44**

Note S4: Body MRI processing details **45**

Note S5: Brain MRI Quality control **46**

Note S6: Linear regression models using the *lm* in r **47**

Note S7: Sample description analyses of anthropometric and body composition measures and brain structure. **48**

REFERENCES **51**

Supplemental Figures

Figure S1: Inclusion/exclusion pipeline, including overview of automatic quality control step.

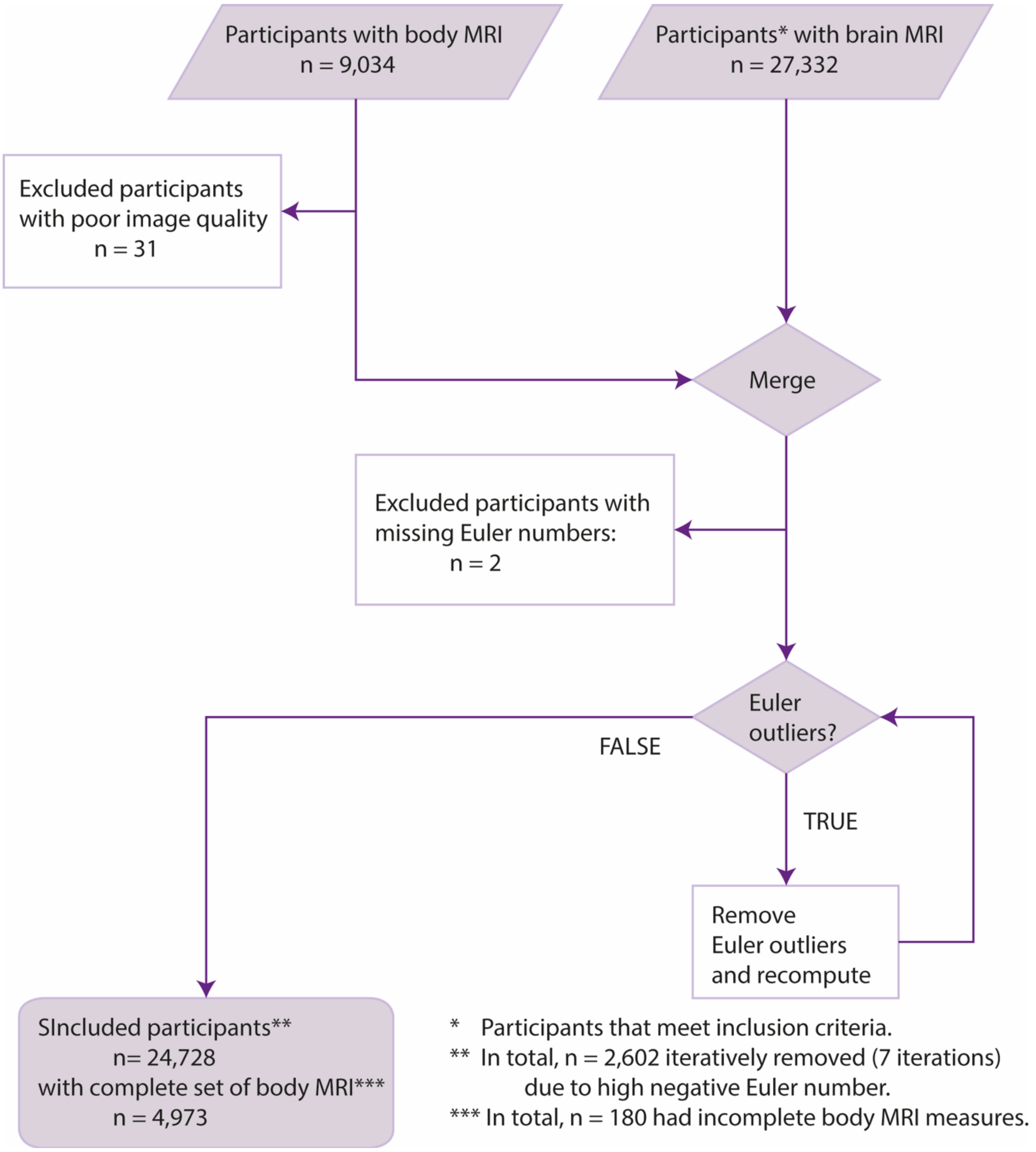

Note: We had brain MRI on 42,068 participants. Initially, 14,736 were excluded based on diagnosis exclusion criteria, or incomplete demographic data. *Abbreviations:* MRI – magnetic resonance imaging.

Figure S2: Distribution continuous demographic variables for the whole sample (n=24,728).

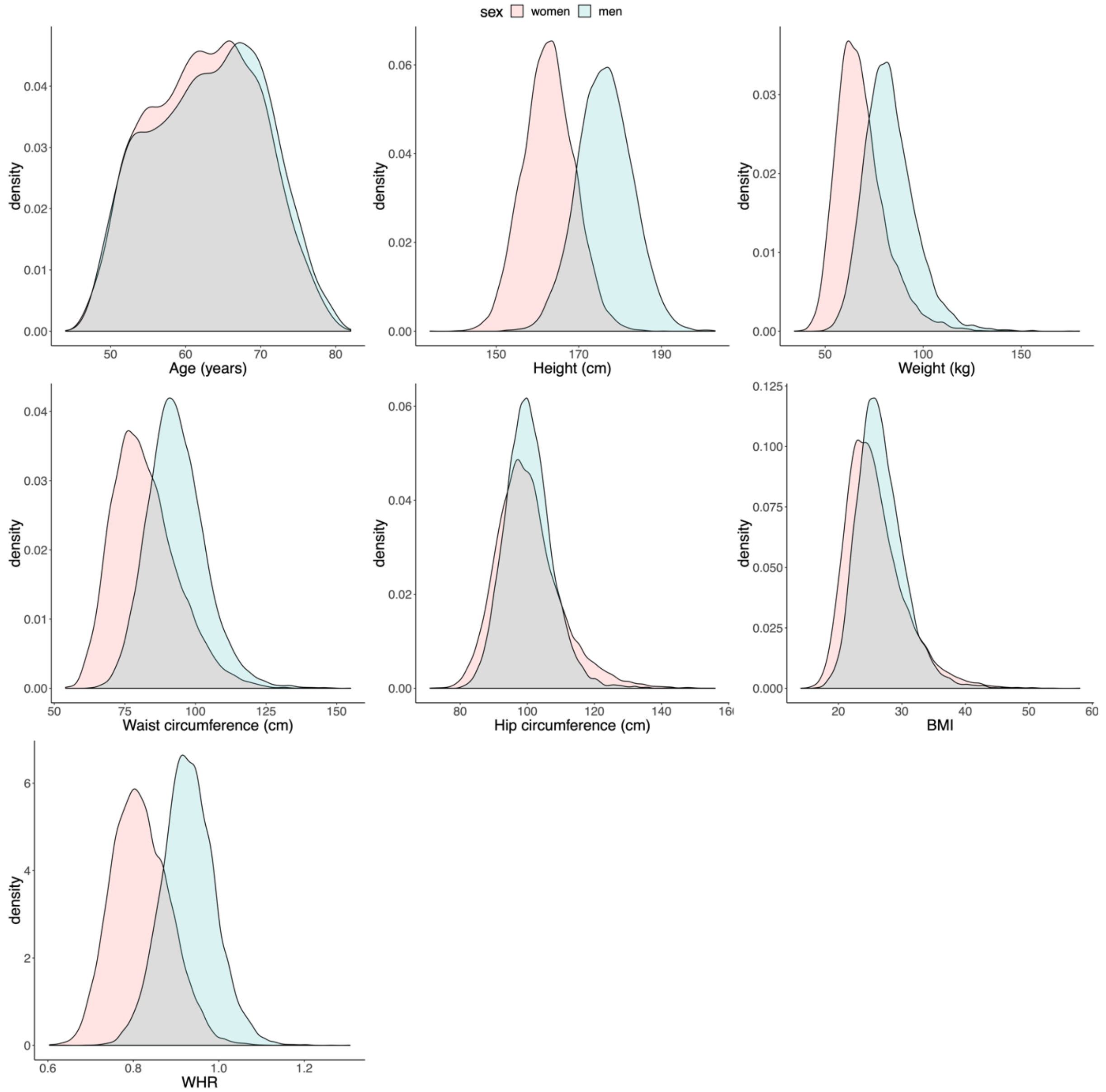

Notes: BMI is computed as weight in kg / (height in meters)^2 and WHR is computed as waist circumference in cm / hip circumference in cm. *Abbreviations:* BMI - body mass index; WHR – waist-to-hip ratio.

Figure S3: Distribution of continuous demographic variables for the body MRI subsample (n=4,973).

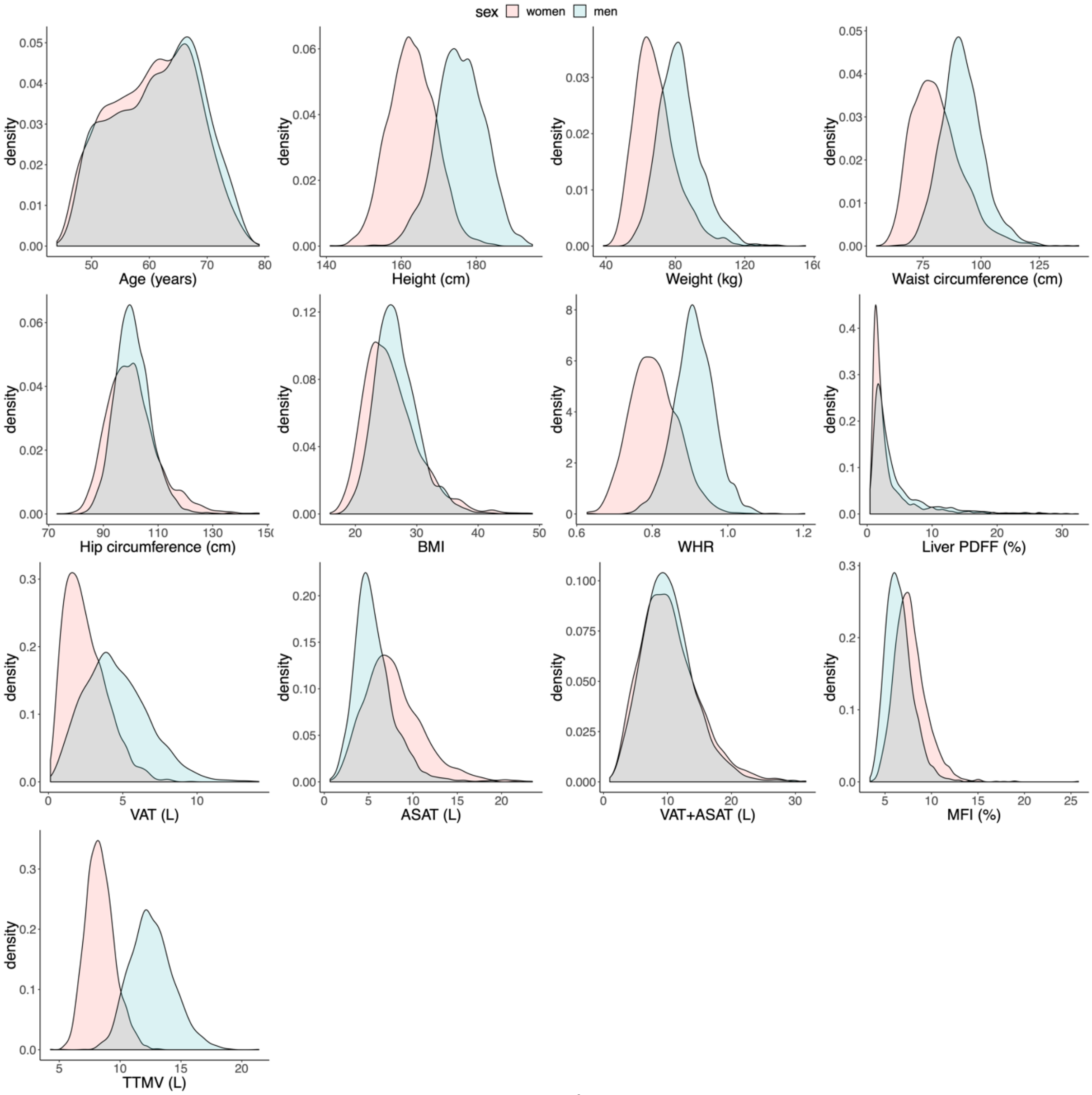

Notes: BMI is computed as weight in kg / (height in meters)^2 and WHR is computed as waist circumference in cm / hip circumference in cm. *Abbreviations:* ASAT – abdominal subcutaneous adipose tissue; BMI - body mass index; L – liter; MFI – muscle fat infiltration; PDFF – Proton density fat fraction; TTMV - Total thigh muscle volume; VAT – visceral adipose tissue; VAT+ASAT – total abdominal adipose tissue; WHR – waist-to-hip ratio.

Figure S4: Distribution of included brain structures for the whole sample (n=24,728).

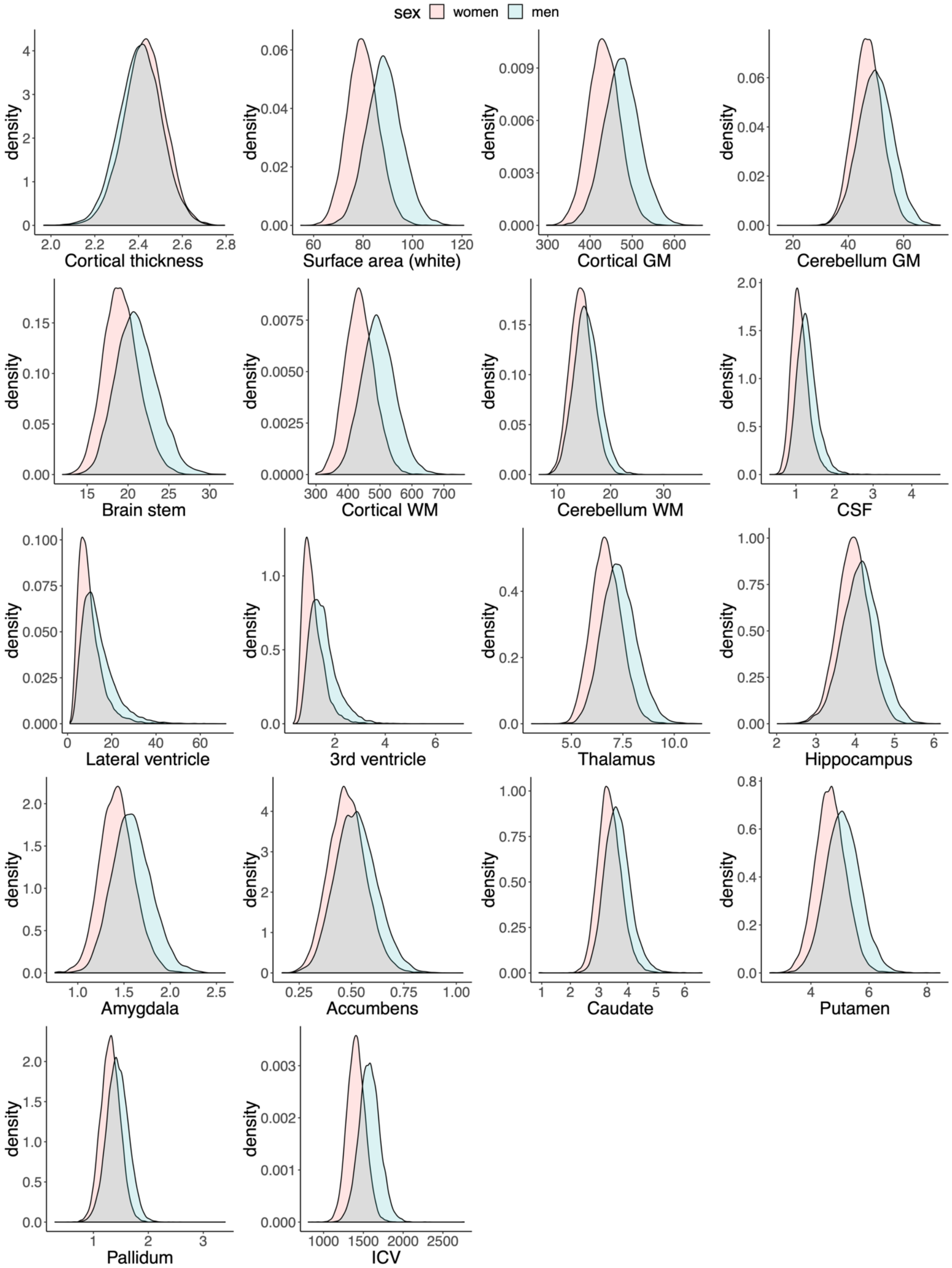

Notes: All brain structures are given in ml (except surface area given in m^2 and cortical thickness given in mm).
Abbreviations: CSF – cerebrospinal fluid; GM – gray matter; ICV – intracranial volume; WM – white matter.

Figure S5: Distribution of included brain structures for the body MRI subsample (n=4,973).

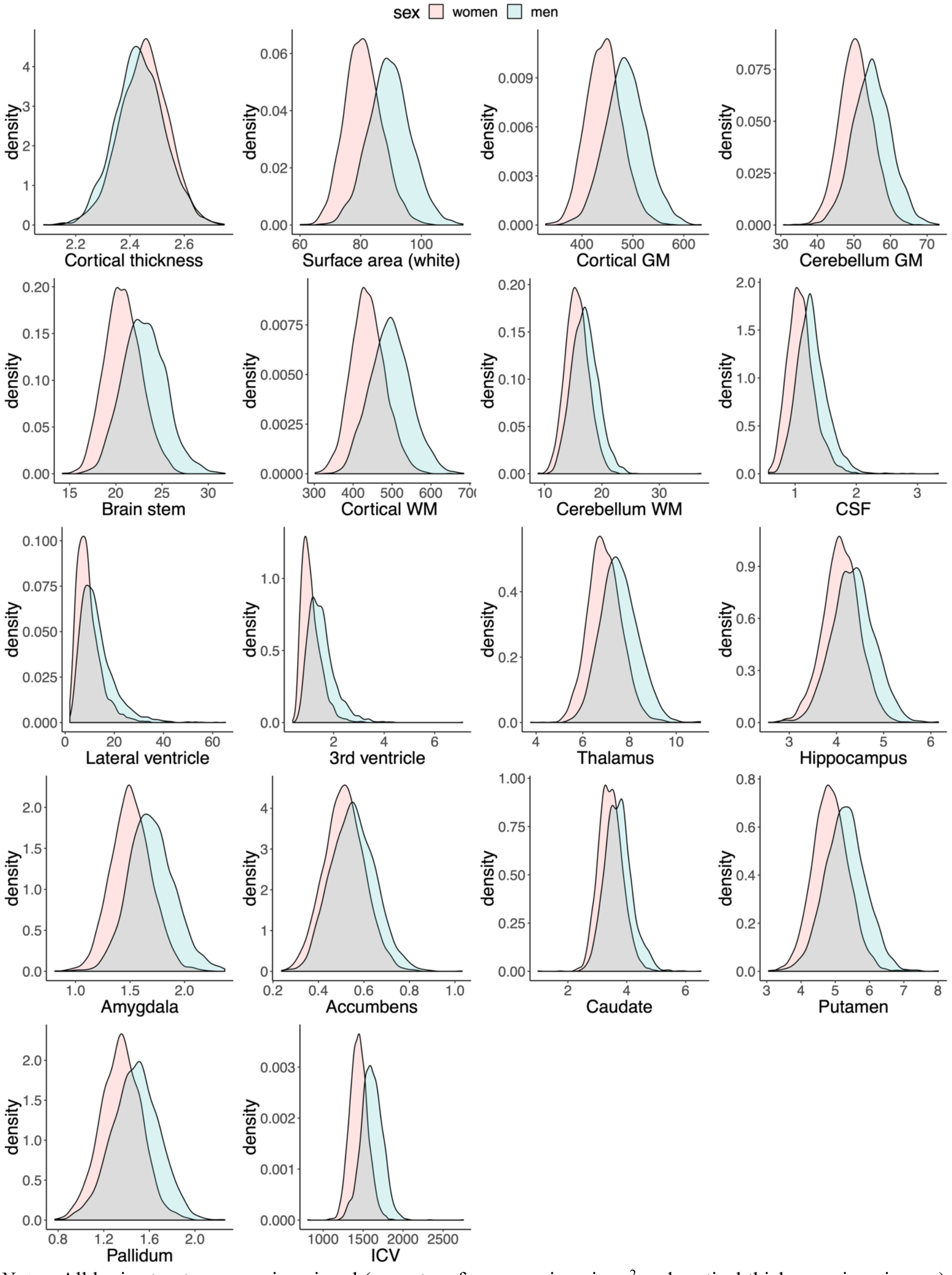

Notes: All brain structures are given in ml (except surface area given in m^2 and cortical thickness given in mm).
Abbreviations: CSF – cerebrospinal fluid; GM – gray matter; ICV – intracranial volume; WM – white matter.

Figure S6: Scatter plots of brain structures with BMI (n=24,728).

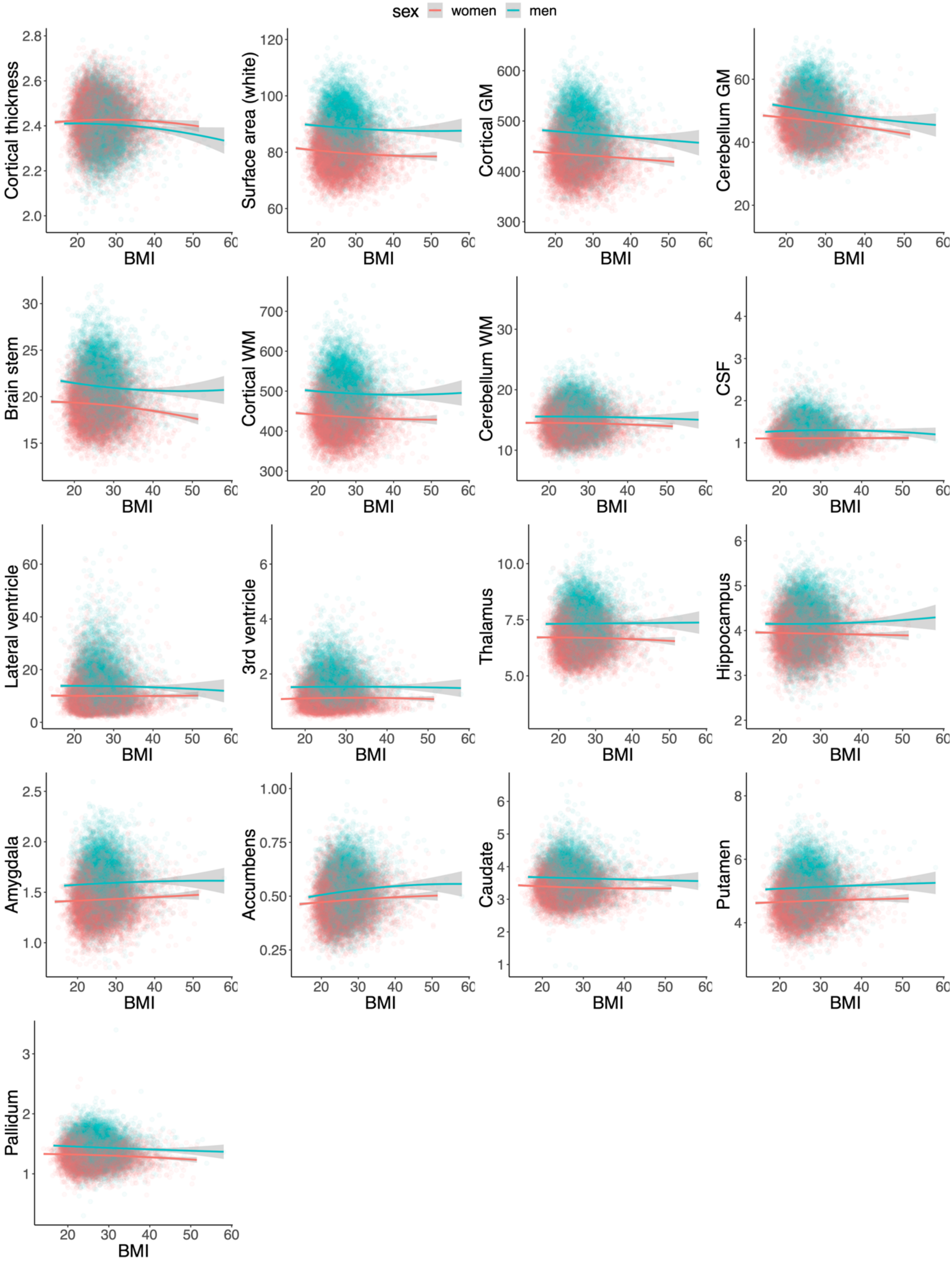

Notes: Regression lines are modeled as brain structure = BMI + BMI^2. The 95% confidence intervals are indicated in gray. Illustrations were split on sex (commonly a significant factor in neuroimaging studies), but were not adjusted for other confounders. All brain structures are given in ml (except surface area given in m^2 and cortical thickness given in mm). BMI is computed as weight in kg / (height in meters)^2. *Abbreviations:* BMI – body mass index.

Figure S7: Scatter plots of brain structures with WHR (n=24,728).

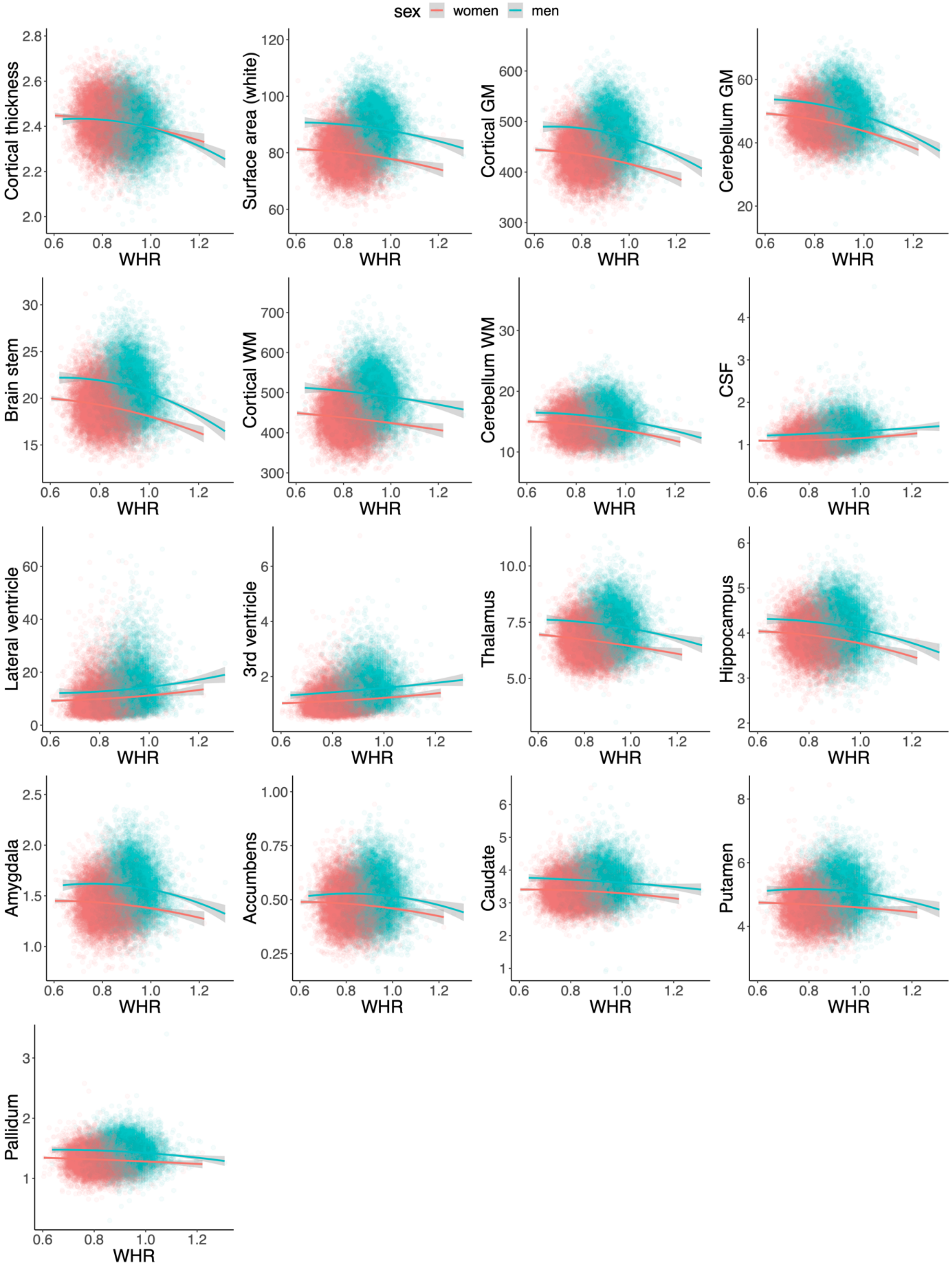

Notes: Regression lines are modeled as brain structure = WHR + WHR^2. The 95% confidence intervals are indicated in gray. Illustrations were split on sex (commonly a significant factor in neuroimaging studies), but were not adjusted for other confounders. All brain structures are given in ml (except surface area given in m^2 and cortical thickness given in mm). WHR is computed as waist circumference in cm / hip circumference in cm. *Abbreviations:* WHR – waist-to-hip ratio.

Figure S8: Scatter plots of brain structures with waist circumference (n=24,728).

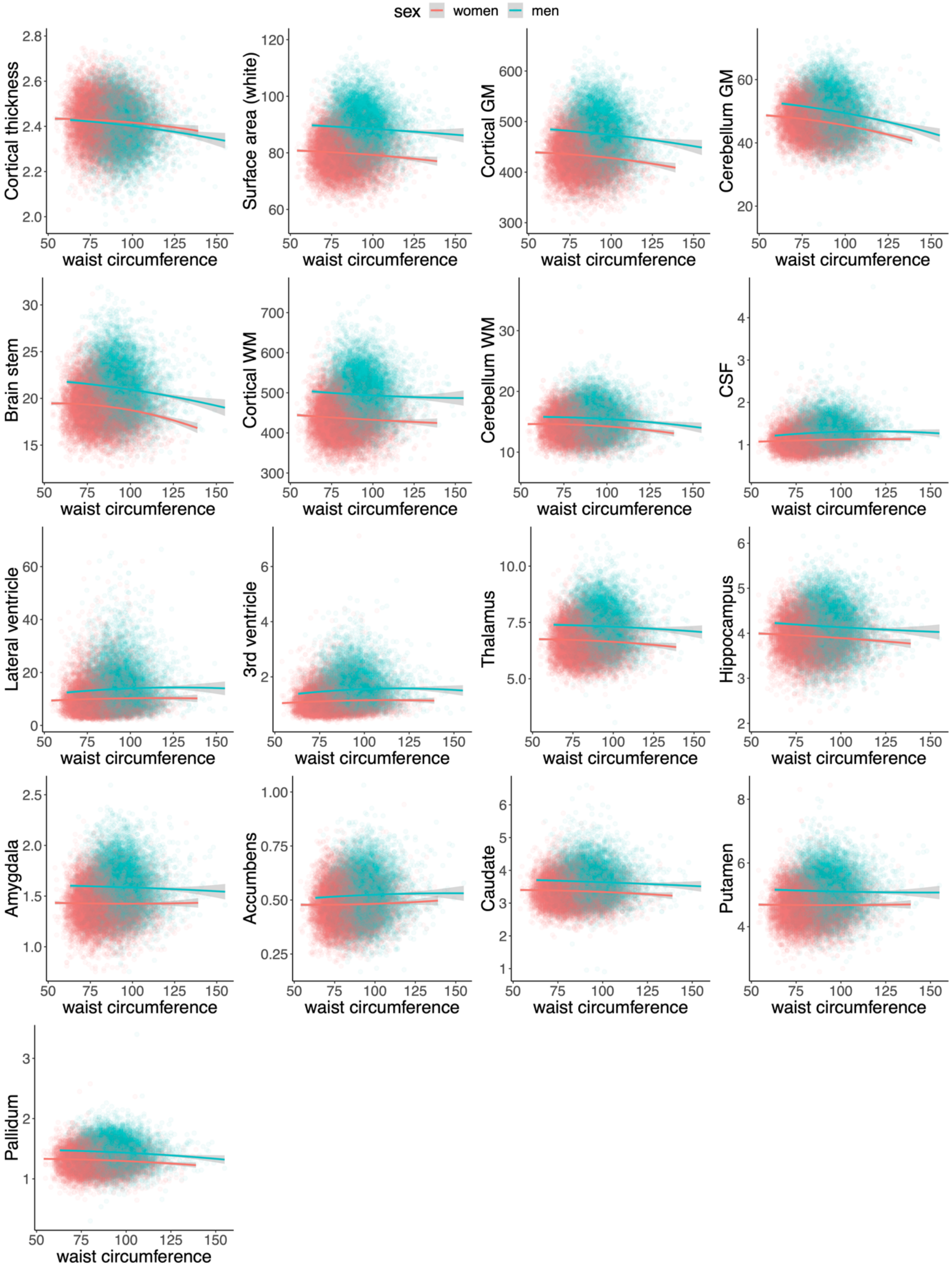

Notes: Regression lines are modeled as brain structure = waist circumference + waist circumference^2. The 95% confidence intervals are indicated in gray. Illustrations were split on sex (commonly a significant factor in neuroimaging studies), but were not adjusted for other confounders. All brain structures are given in ml (except surface area given in m^2 and cortical thickness given in mm). Waist circumference is given in cm.

Figure S9: Scatter plots of brain structures with liver PDFF (n=4,973).

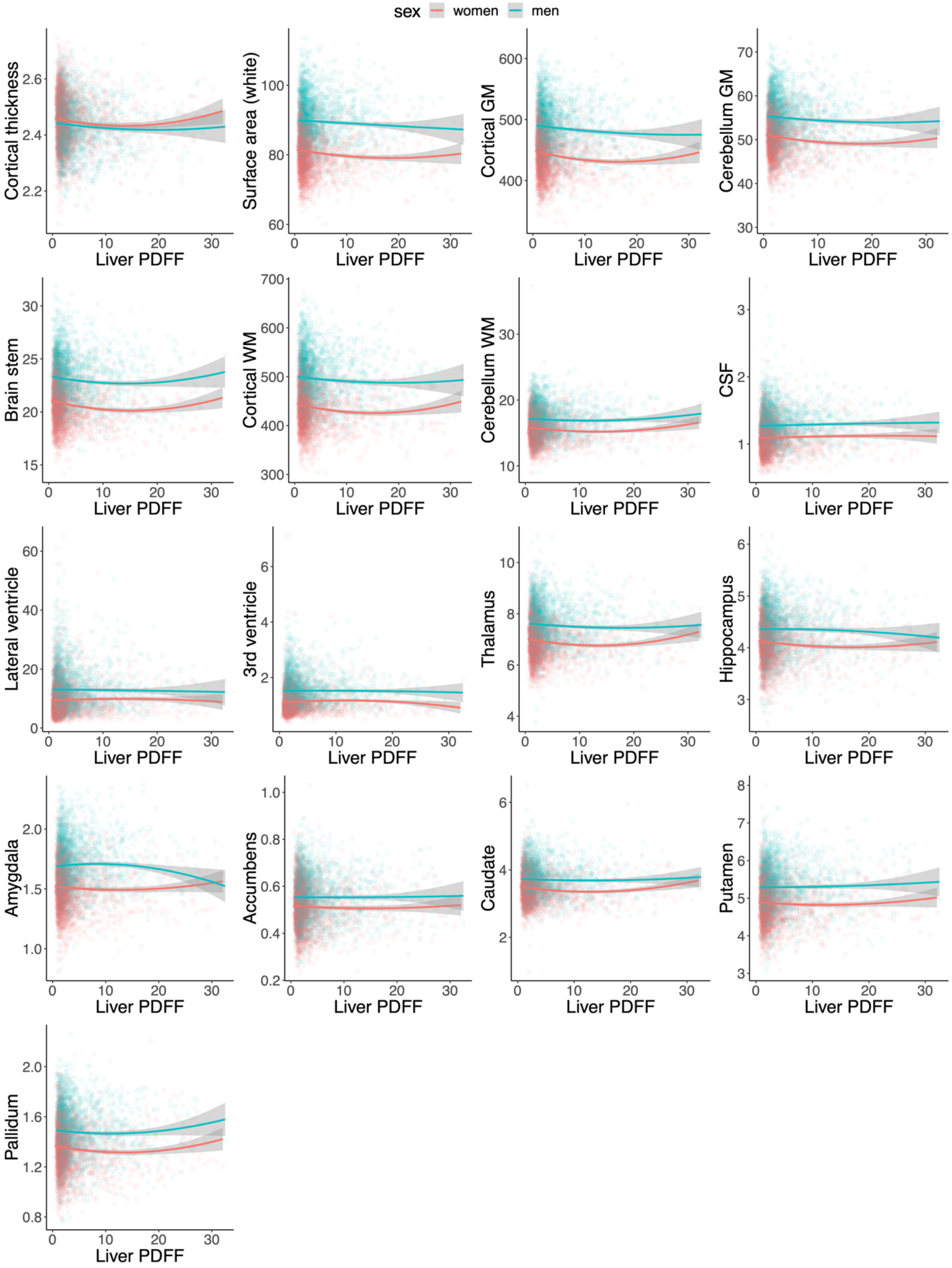

Notes: Regression lines are modeled as brain structure = liver PDFF + liver PDFF^2. The 95% confidence intervals are indicated in gray. Illustrations were split on sex (commonly a significant factor in neuroimaging studies), but were not adjusted for other confounders. All brain structures are given in ml (except surface area given in m^2 and cortical thickness given in mm). Liver PDFF is given in %. *Abbreviations*: PDFF – proton density fat fraction.

Figure S10: Scatter plots of brain structures with VAT (n=4,973).

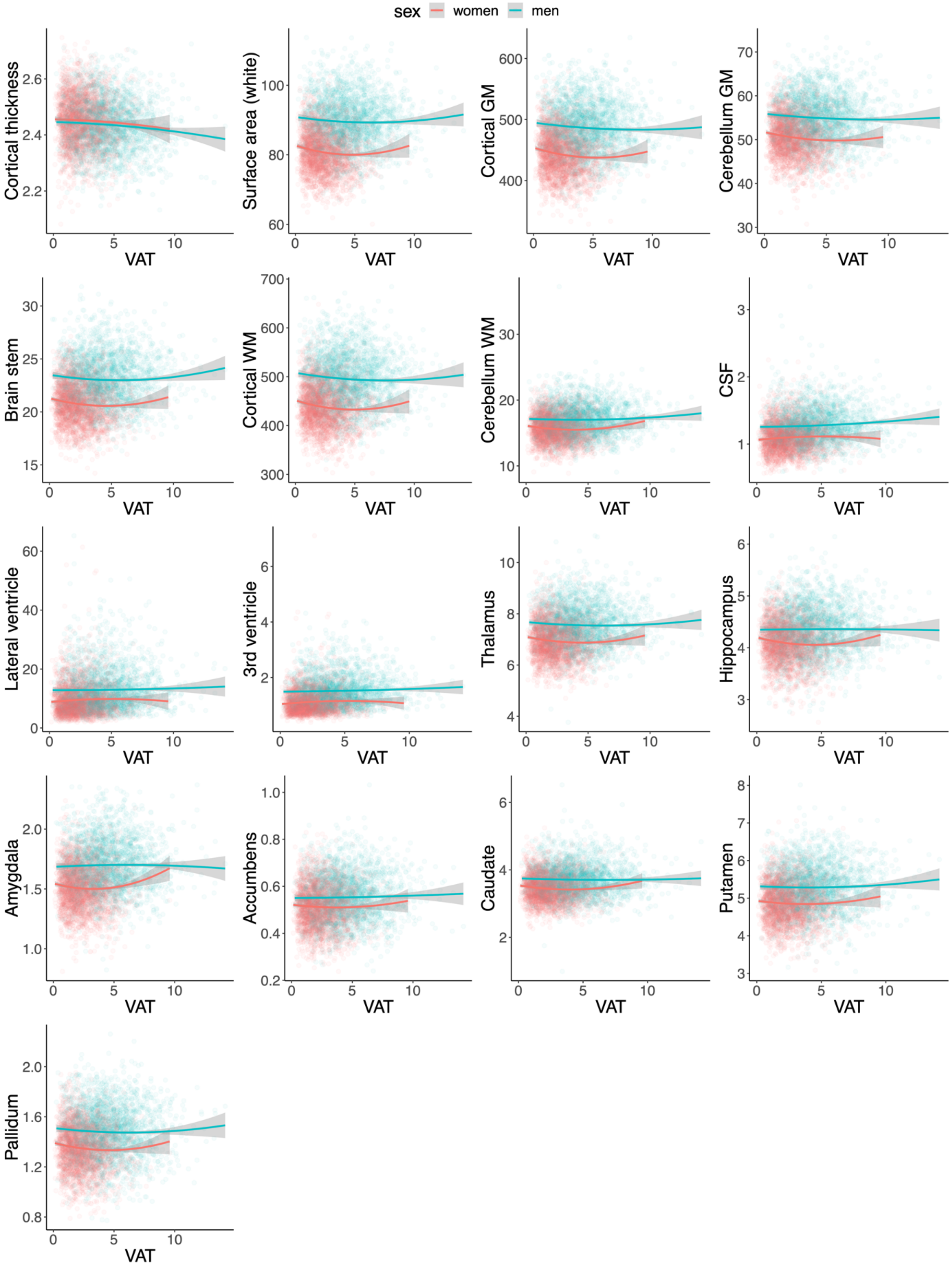

Notes: Regression lines are modeled as brain structure = VAT + VAT^2. The 95% confidence intervals are indicated in gray. Illustrations were split on sex (commonly a significant factor in neuroimaging studies), but were not adjusted for other confounders. All brain structures are given in ml (except surface area given in m^2 and cortical thickness given in mm). VAT is given in L. *Abbreviations*: L – liter; VAT – visceral adipose tissue.

Figure S11: Scatter plots of brain structures with ASAT (n=4,973).

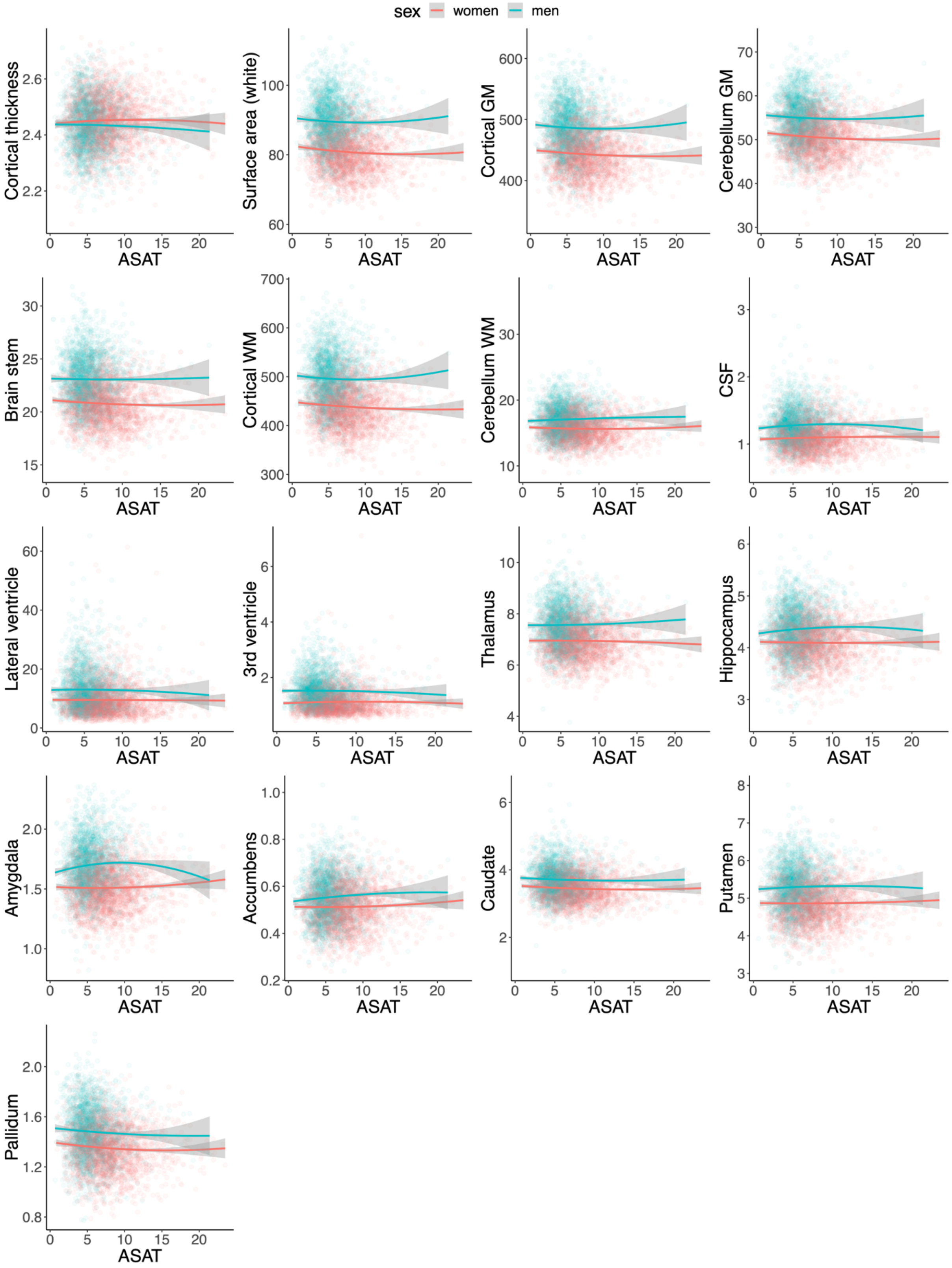

Notes: Regression lines are modeled as brain structure = ASAT + ASAT^2. The 95% confidence intervals are indicated in gray. Illustrations were split on sex (commonly a significant factor in neuroimaging studies), but were not adjusted for other confounders. All brain structures are given in ml (except surface area given in m^2 and cortical thickness given in mm), ASAT is given in L. *Abbreviations*: ASAT – abdominal subcutaneous adipose tissue; L – liter.

Figure S12: Scatter plots of brain structures with VAT+ASAT (n=4,973).

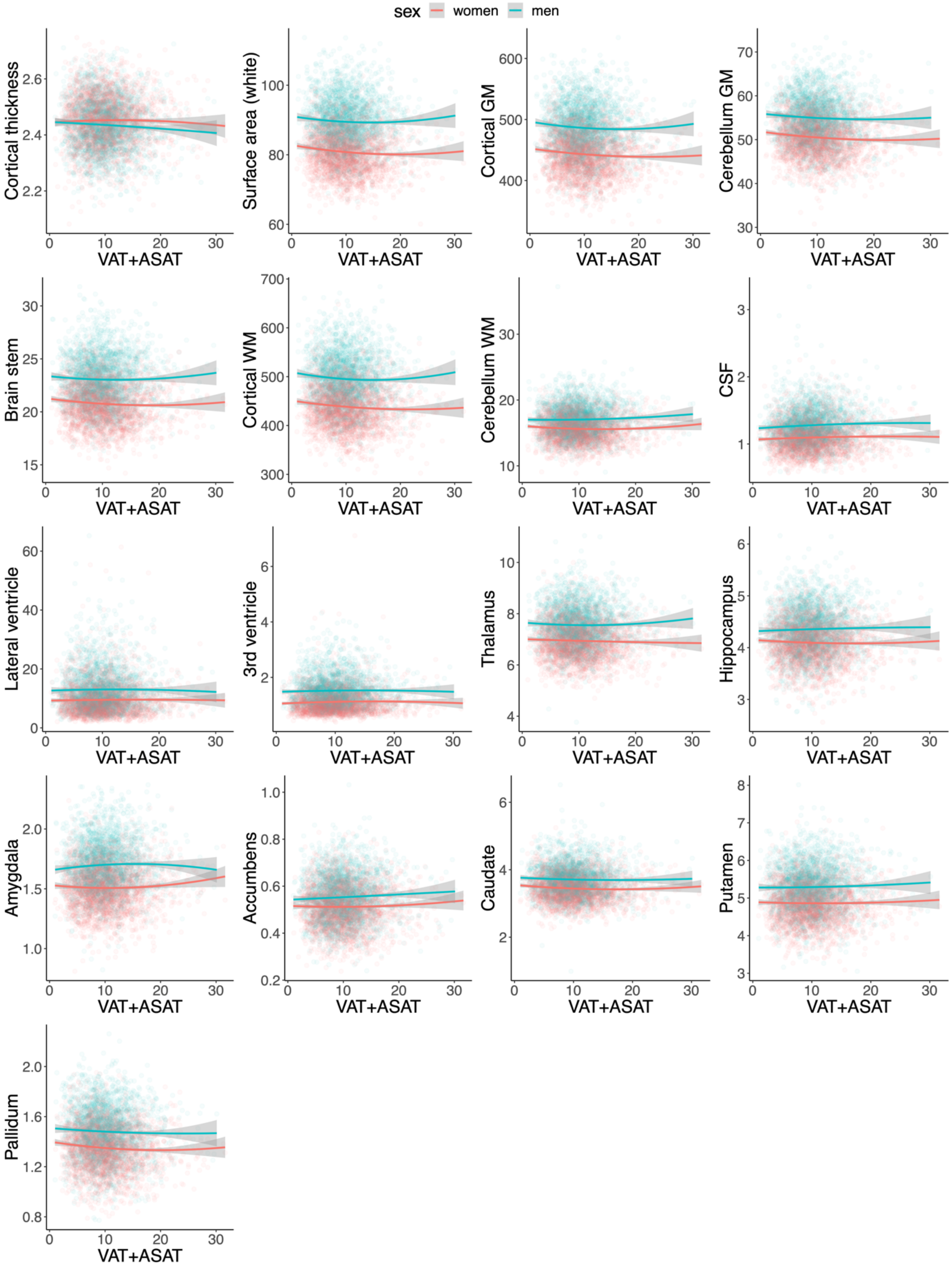

Notes: Regression lines are modeled as brain structure = (VAT+ASAT) + $(VAT+ASAT)^2$. The 95% confidence intervals are indicated in gray. Illustrations were split on sex (commonly a significant factor in neuroimaging studies), but were not adjusted for other confounders. All brain structures are given in ml (except surface area given in m^2 and cortical thickness given in mm). VAT+ASAT is given in L. *Abbreviations*: L – liter; VAT+ASAT – total abdominal adipose tissue.

Figure S13: Scatter plots of brain structures with MFI (n=4,973).

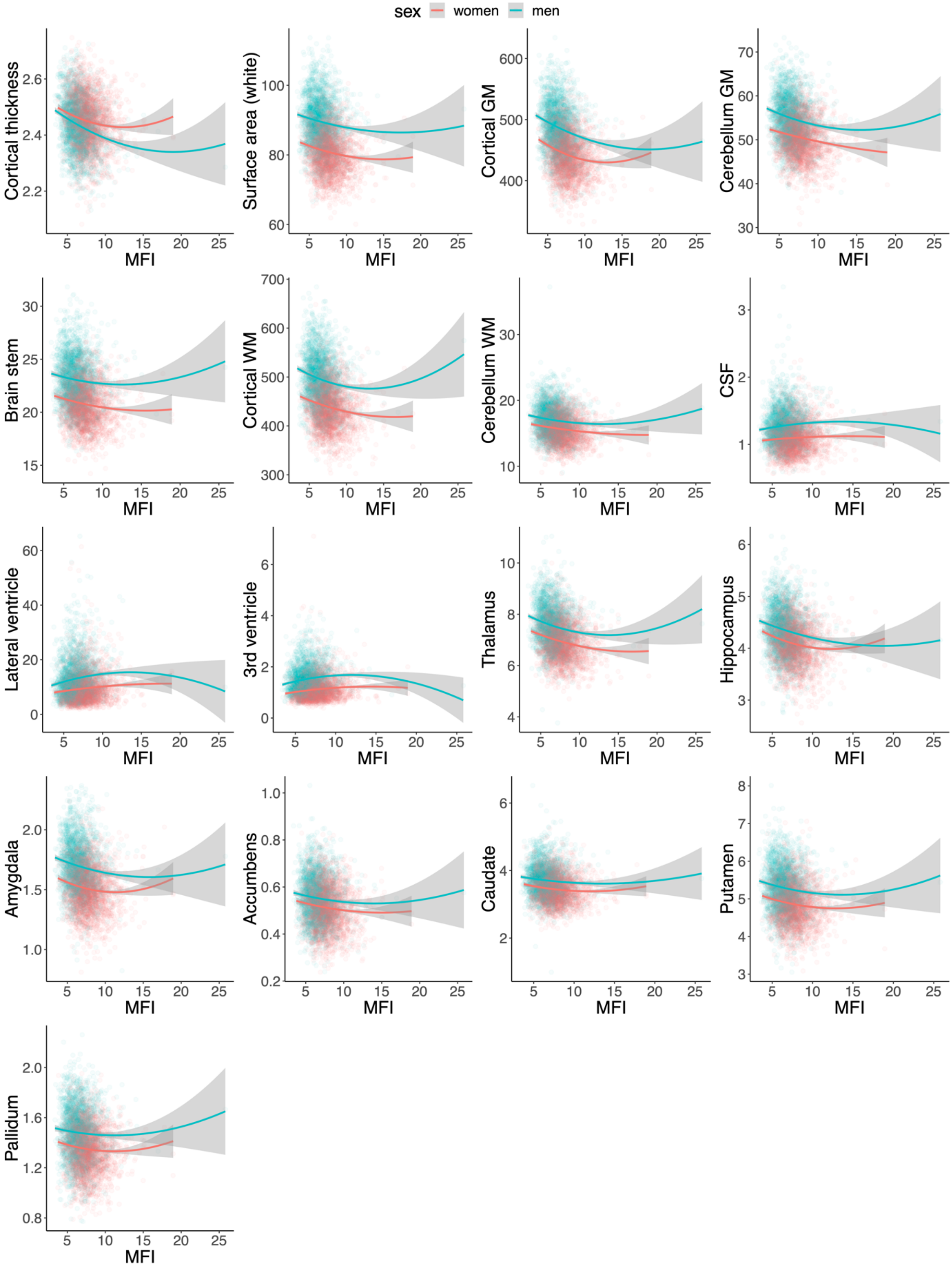

Notes: Regression lines are modeled as brain structure = MFI + MFI^2. The 95% confidence intervals are indicated in gray. Illustrations were split on sex (commonly a significant factor in neuroimaging studies), but were not adjusted for other confounders. All brain structures are given in ml (except surface area given in m^2 and cortical thickness given in mm). MFI is given in %. *Abbreviations*: MFI – muscle fat infiltration.

Figure S14: Scatter plots of brain structures with TTMV (n=4,973).

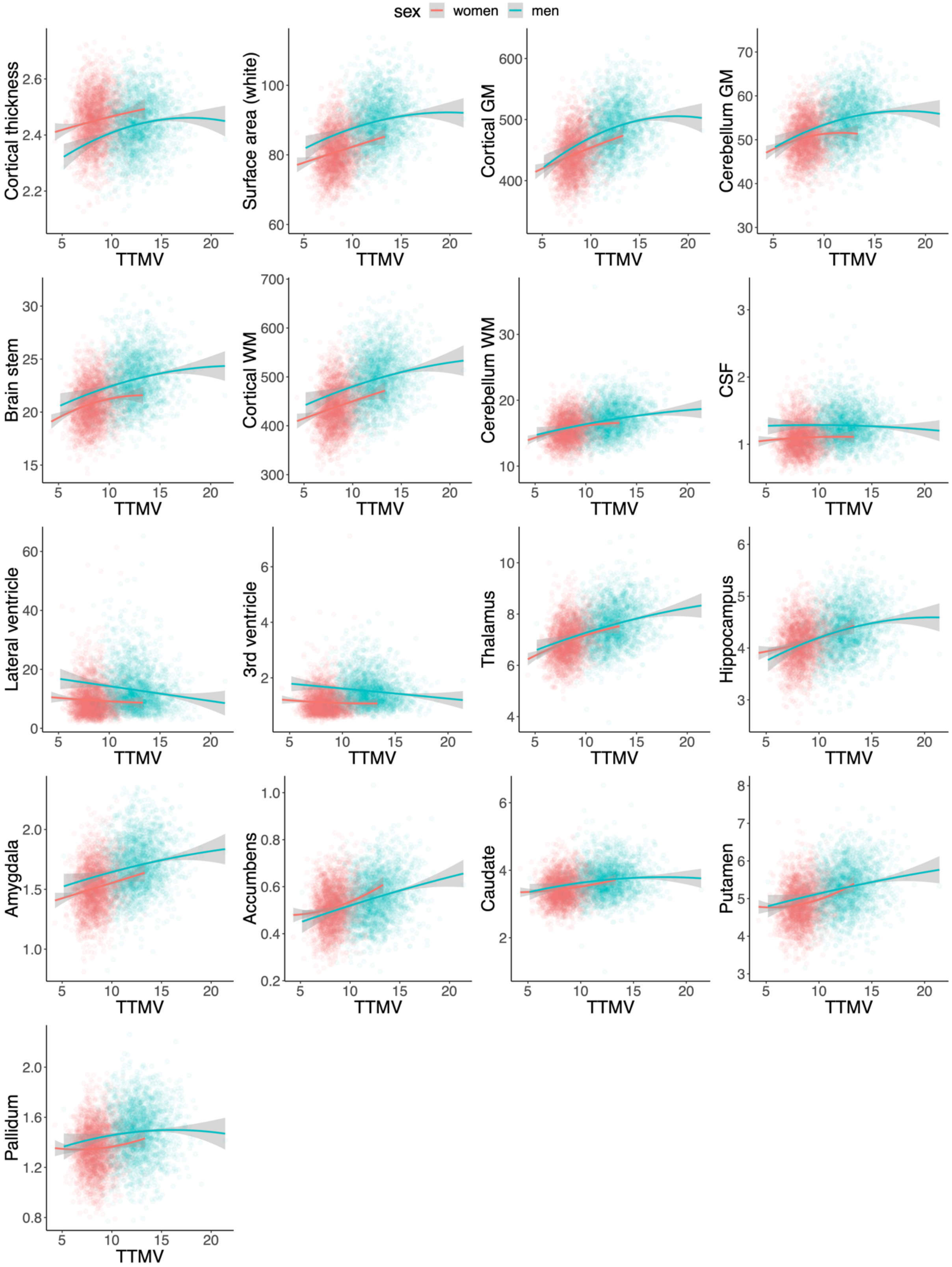

Notes: Regression lines are modeled as brain structure = TTMV + $TTMV^2$. The 95% confidence intervals are indicated in gray. Illustrations were split on sex (commonly a significant factor in neuroimaging studies), but were not adjusted for other confounders. All brain structures are given in ml (except surface area given in m^2 and cortical thickness given in mm). TTMV is given in L. *Abbreviations*: L – liter; TTMV – total thigh muscle volume.

Figure S15: Residual versus fitted value plots and Q-Q plots for models with anthropometric measures as dependent variable, with (left) and without (right) log-transformation of dependent variables (full sample; n=24,728).

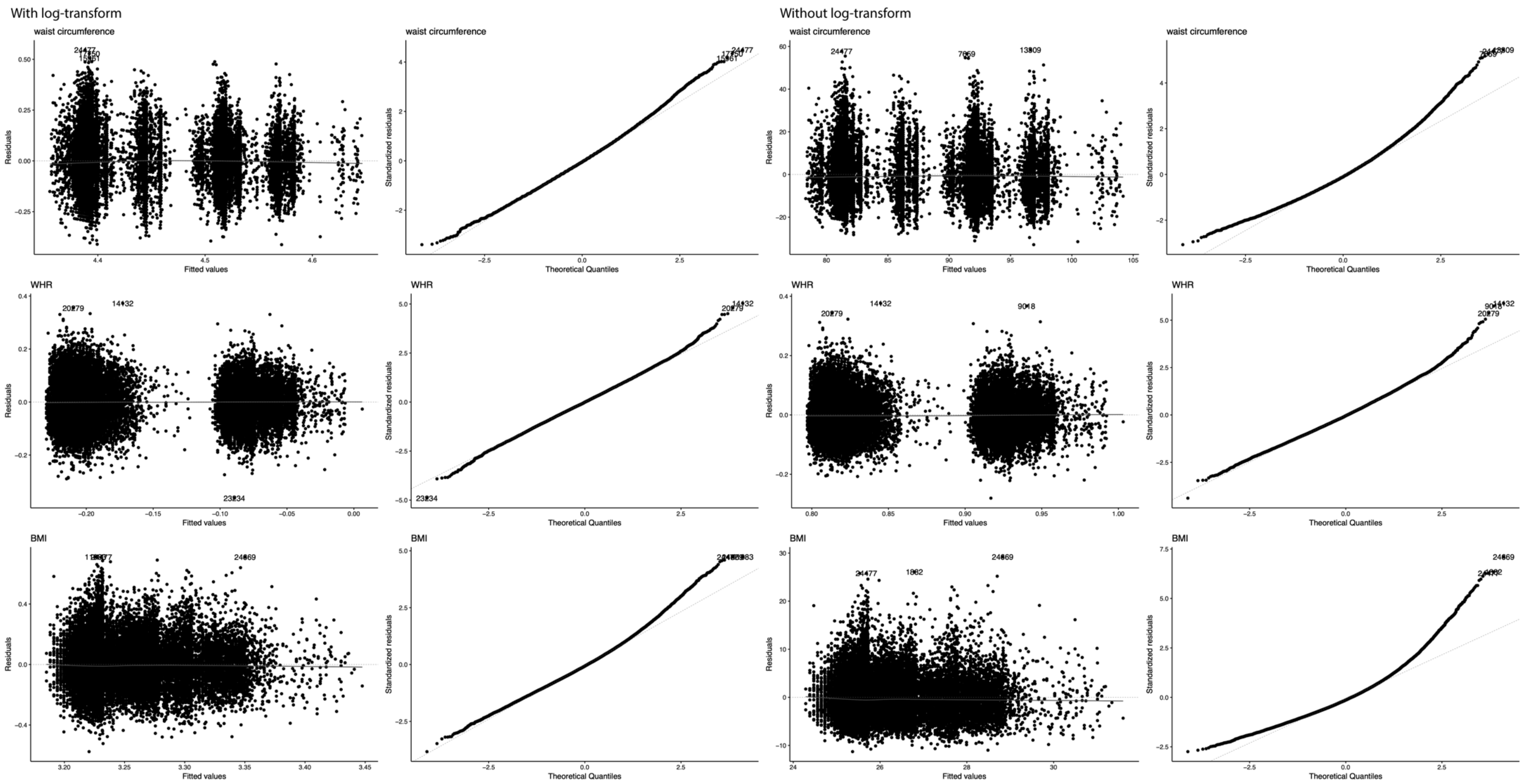

Notes: Residuals derived from model 1c. *Abbreviations:* BMI – body mass index; WHR – waist-hip-ratio.

Figure S16: Residual versus fitted value plots and Q-Q plots for models with models with anthropometric and body composition measures as dependent variable: with (left) and without (right) log-transformation of dependent variables (body MRI subsample; n=4,973).

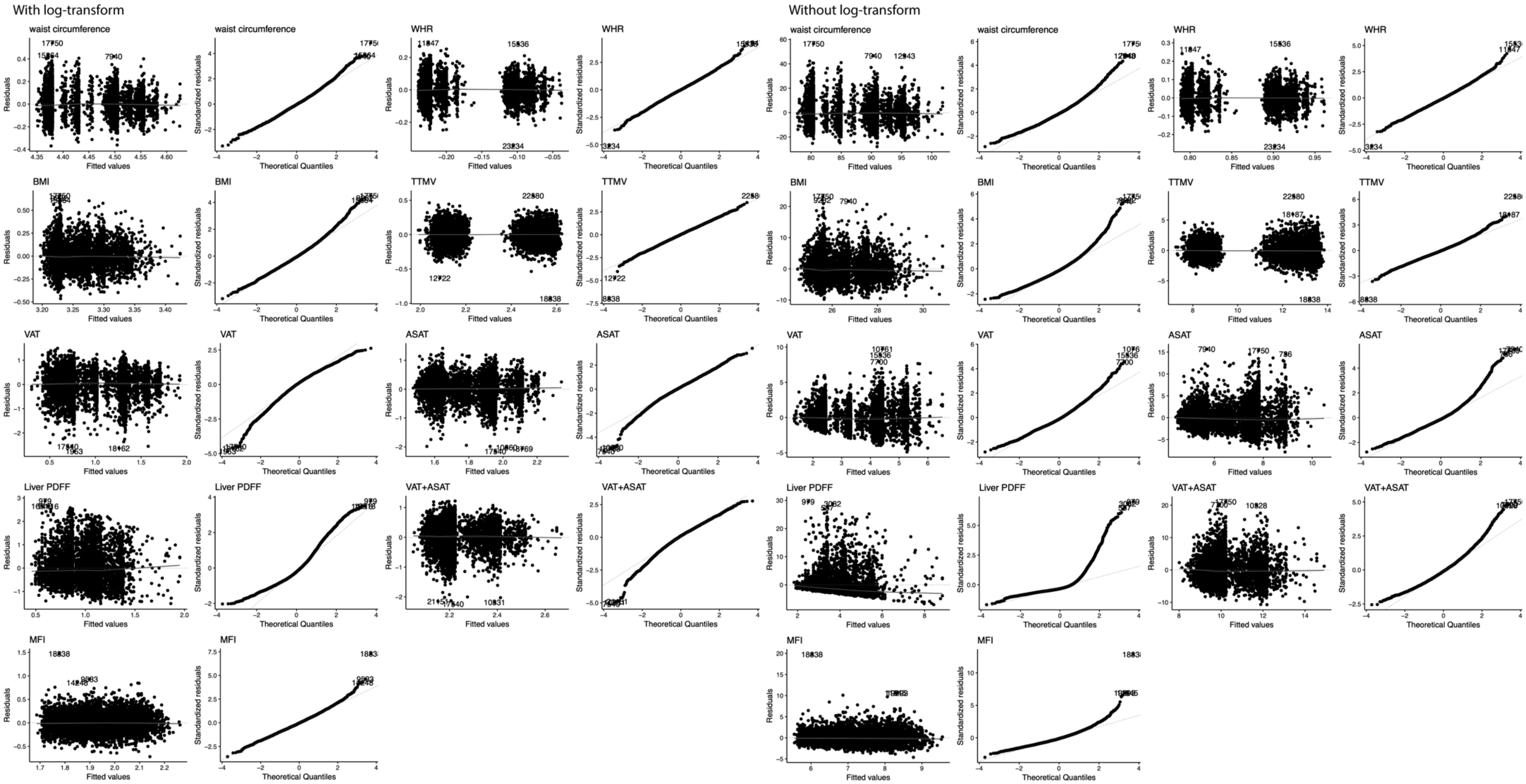

Notes: Residuals derived from model 1c. *Abbreviations:* ASAT – abdominal subcutaneous adipose tissue; BMI – body mass index; MFI – muscle fat infiltration; PDFF – proton density fat fraction; TTMV – total thigh muscle volume; VAT – visceral adipose tissue; VAT + ASAT – total abdominal adipose tissue; WHR – waist-hip-ratio.

Figure S17: Residual versus fitted value plots and Q-Q plots for models investigating brain structures as dependent variable after log-transformation of CSF, lateral ventricle, and 3rd ventricle (full sample; n=24,728).

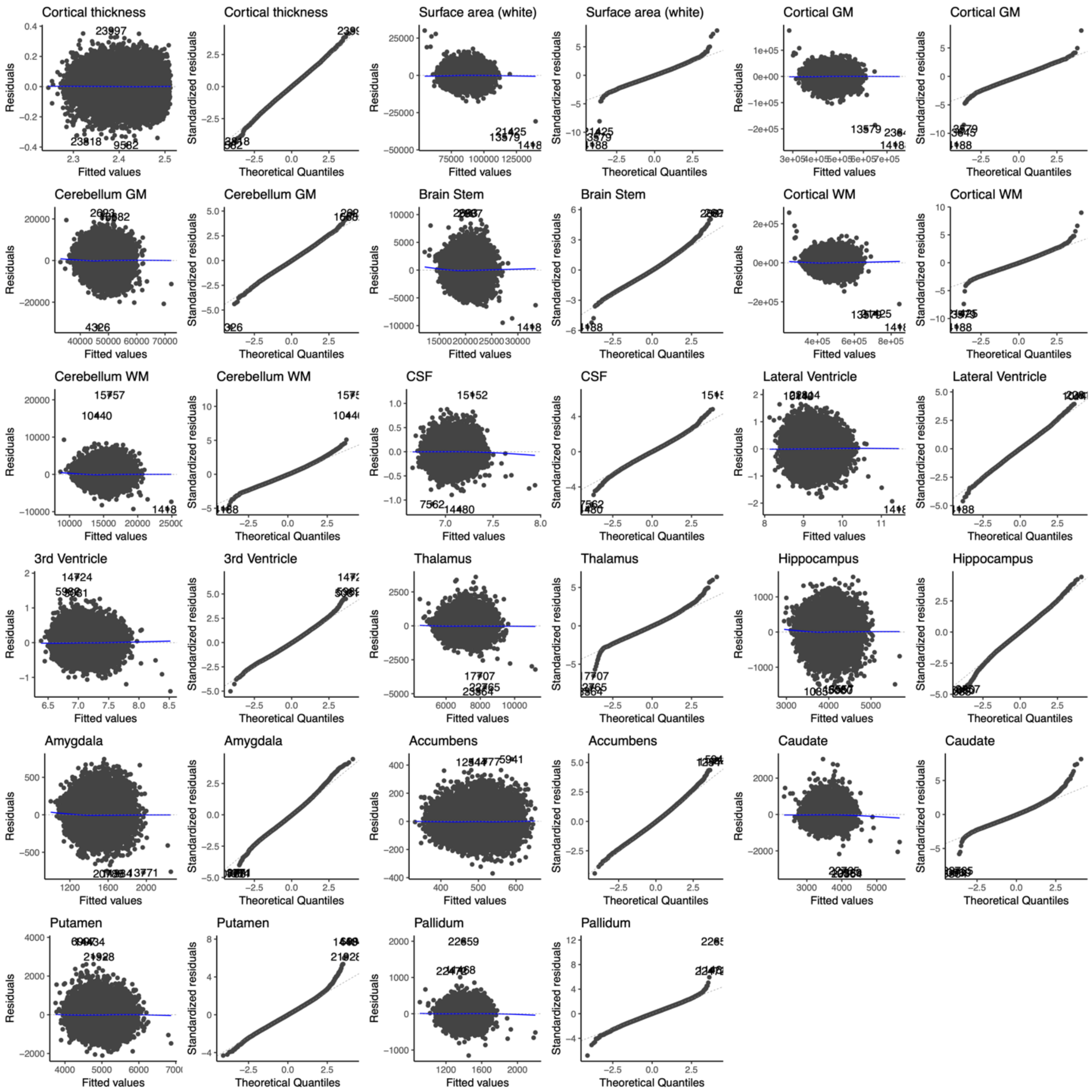

Notes: Residuals derived from model 2c with waist-hip-ratio (WHR) as independent variable. *Abbreviations:* CSF - cerebrospinal fluid; GM – gray matter; WM – white matter.

Figure S18: Residual versus fitted value plots and Q-Q plots for CSF, lateral ventricle, and 3rd ventricle as the respective dependent variables: with (left) and without (right) log-transformation (full sample; n=24,728).

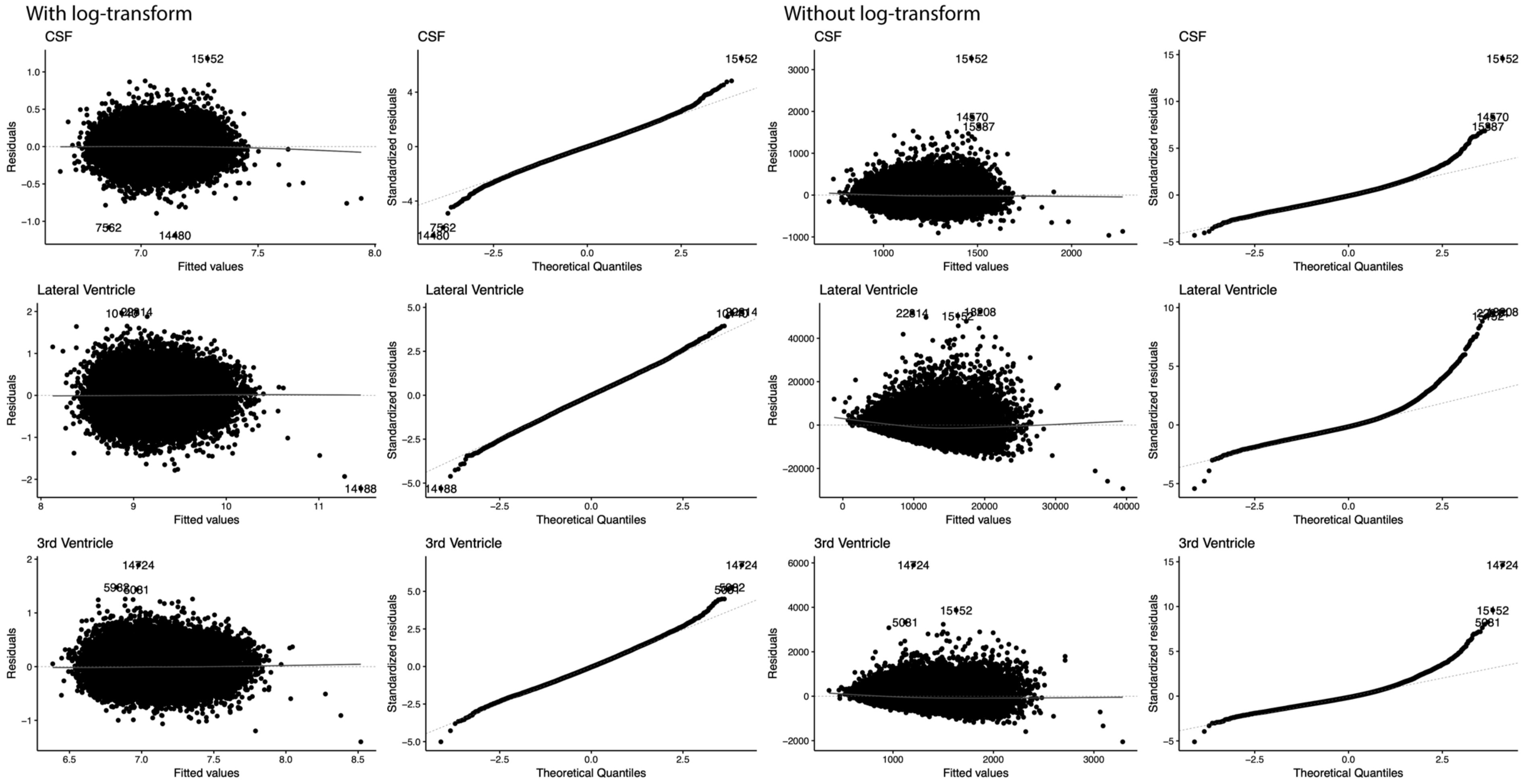

Notes: Residuals derived from model 2c with waist-hip-ratio (WHR) as independent variable. *Abbreviations:* CSF - cerebrospinal fluid.

Figure S19: Residual versus fitted value plots and Q-Q plots for models investigating brain structures as dependent variable after log-transformation of CSF, lateral ventricle, and 3rd ventricle (body MRI subsample; n=4,973).

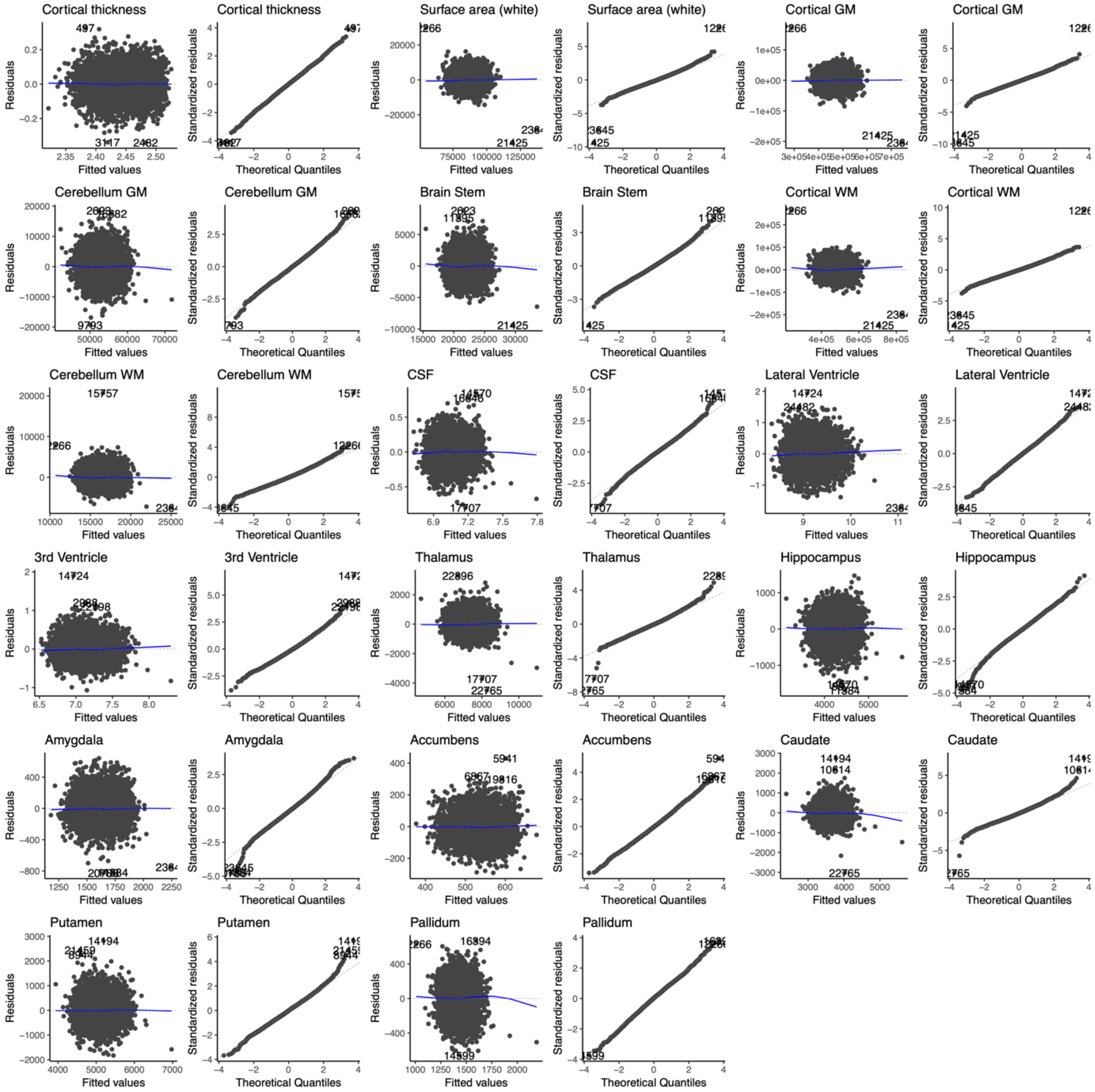

Notes: Residuals derived from model 2c with waist-hip-ratio (WHR) as independent variable. *Abbreviations:* CSF - cerebrospinal fluid; GM – gray matter; WM – white matter.

Figure S20: Evaluation of multiple linear regression model residuals for normality using residual versus fitted value plots and Q-Q plots for CSF, lateral ventricle, and 3rd ventricle as the respective dependent variables: with (left) and without (right) log-transformation (body MRI subsample; n=4,973).

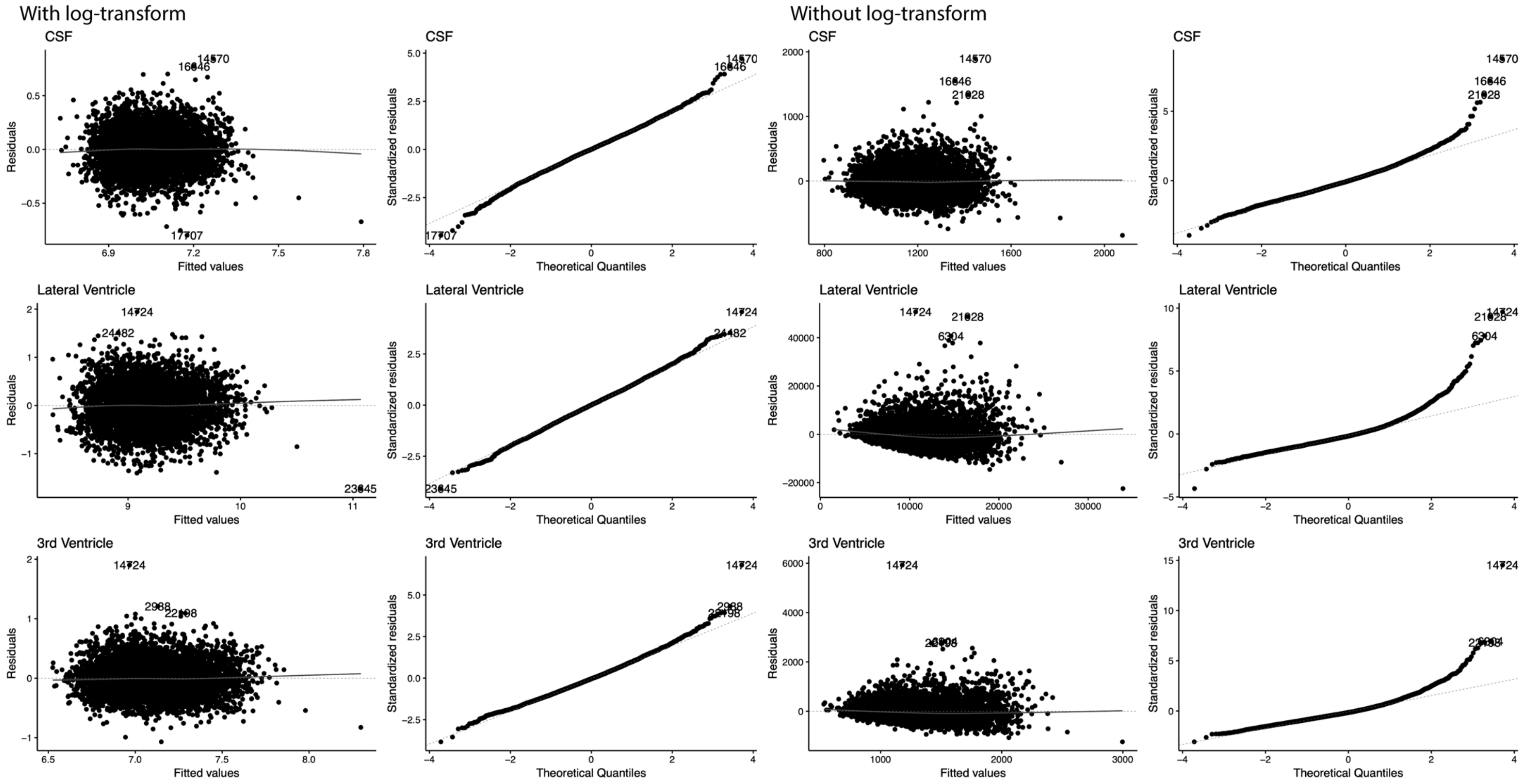

Notes: Residuals derived from model 2c with waist-hip-ratio (WHR) as independent variable. *Abbreviations:* CSF - cerebrospinal fluid; GM – gray matter; WM – white matter.

Figure S21: Body-brain associations in healthy for bilateral measures of brain structure (n=24,728).

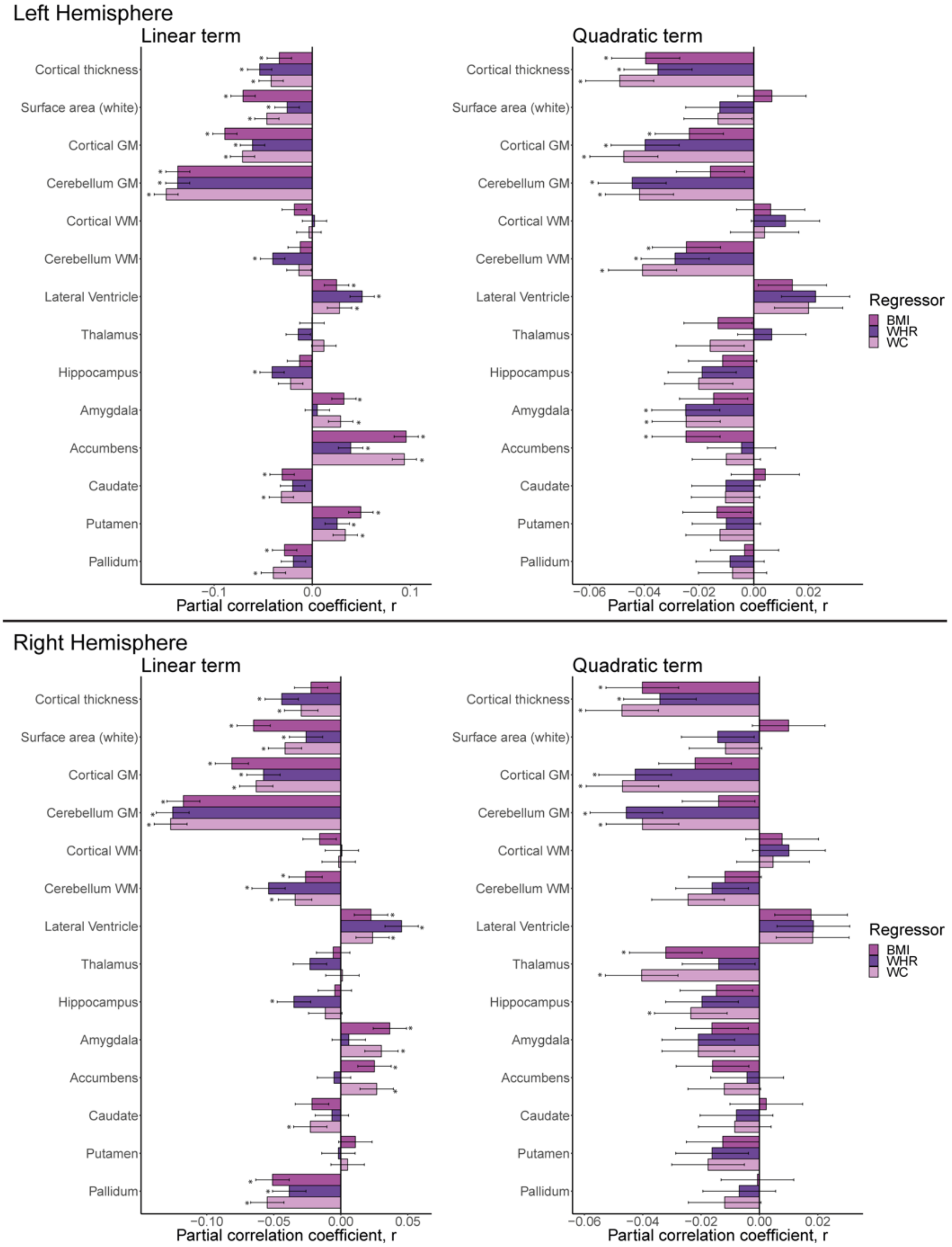

Notes: Results using model 2c to investigates body-brain connections through the inclusion of linear and quadratic term of anthropometric measures for left (top) and right (bottom) hemisphere structures. The regression model was adjusted for age, age^2, sex, age-by-sex, age^2-by-sex, intracranial volume (except cortical thickness), lifestyle/metabolic factors, Euler number, and site. Significant associations indicated by *. Dependent variables CSF, lateral/3rd ventricle were log-transformed. *Abbreviations:* BMI – body mass index; GM – gray matter; WC – waist circumference; WHR – waist-to-hip ratio; WM – white matter.

Figure S22-a: Linear body-brain associations in healthy across models 2a/b/c (n=24,728).

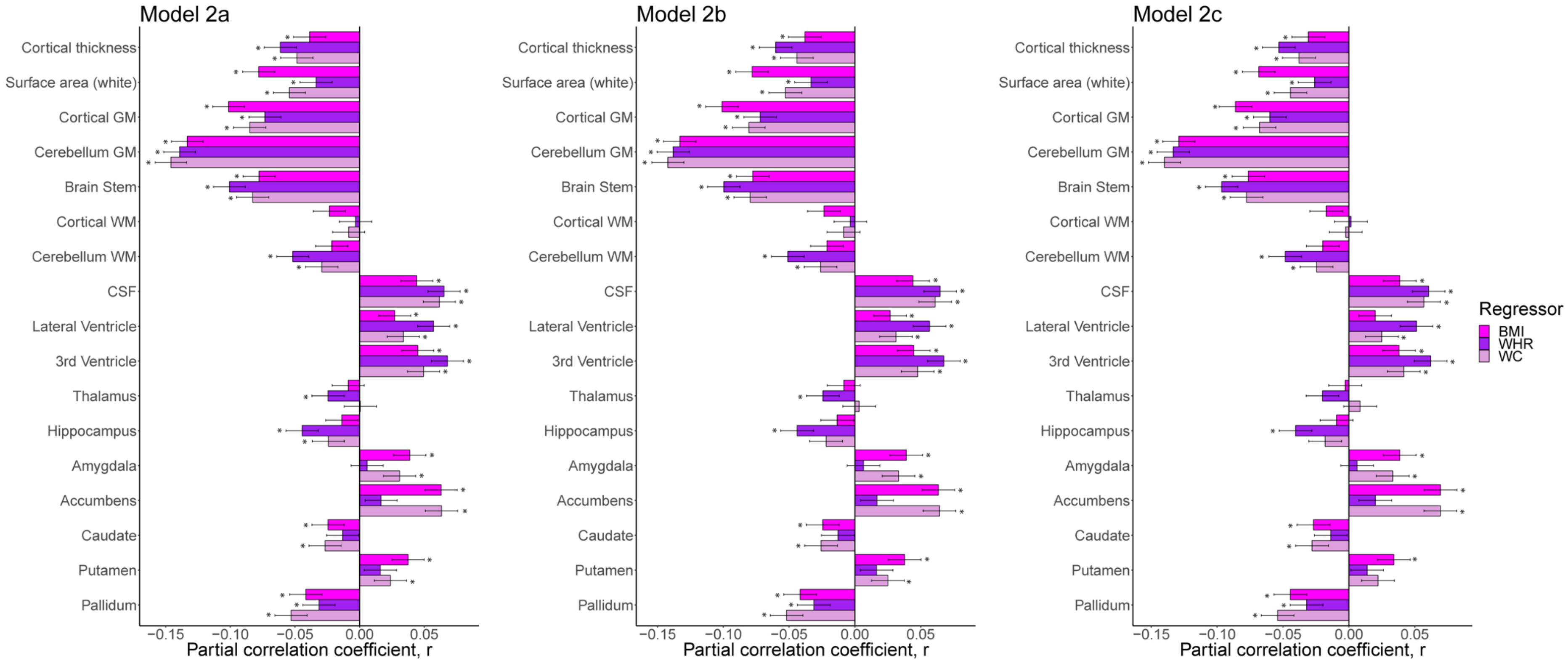

Notes Results from regression model 2a/b/c that investigates body-brain connections through the inclusion of linear and quadratic (only model 2b/c) terms of anthropometric measures, after adjusting for respective model covariates (see manuscript). Figure displays the association between **linear term** and brain structure. The * indicate significance. Dependent variables CSF, lateral/3rd ventricle were log-transformed. *Abbreviations:* BMI – body mass index; GM – gray matter; WC – waist circumference; WHR – waist-to-hip ratio; WM – white matter.

Figure S22-b: Quadratic body-brain associations in healthy across models 2b/c (n=24,728).

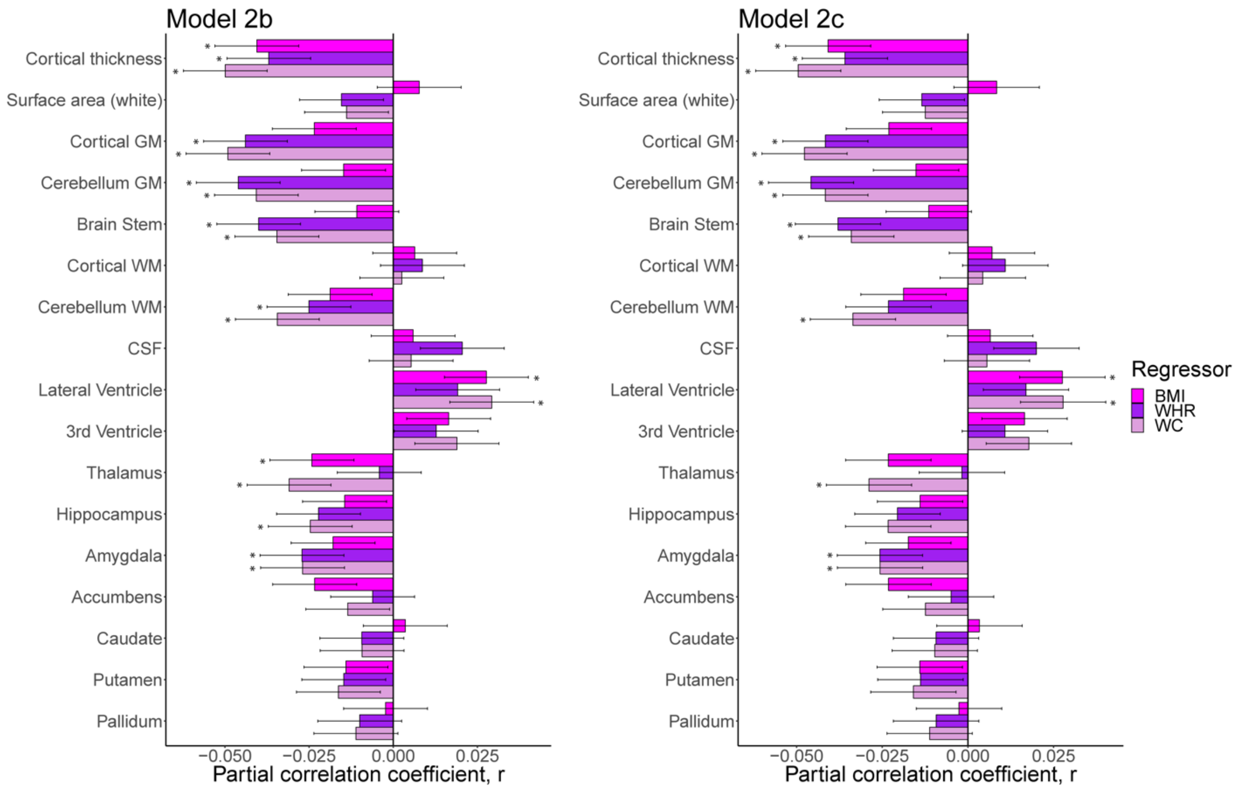

Notes: Results from regression model 2b/c that investigates body-brain connections through the inclusion of linear and quadratic terms of anthropometric measures, after adjusting for respective model covariates (see manuscript). Figure displays the association between **quadratic term** and brain structure. The * indicate significance. Dependent variables CSF, lateral/3rd ventricle were log-transformed. *Abbreviations:* BMI – body mass index; GM – gray matter; WC – waist circumference; WHR – waist-to-hip ratio; WM – white matter.

Figure S23: Quadratic body-brain association patterns across cortical parcellations in healthy individuals (n=24,728).

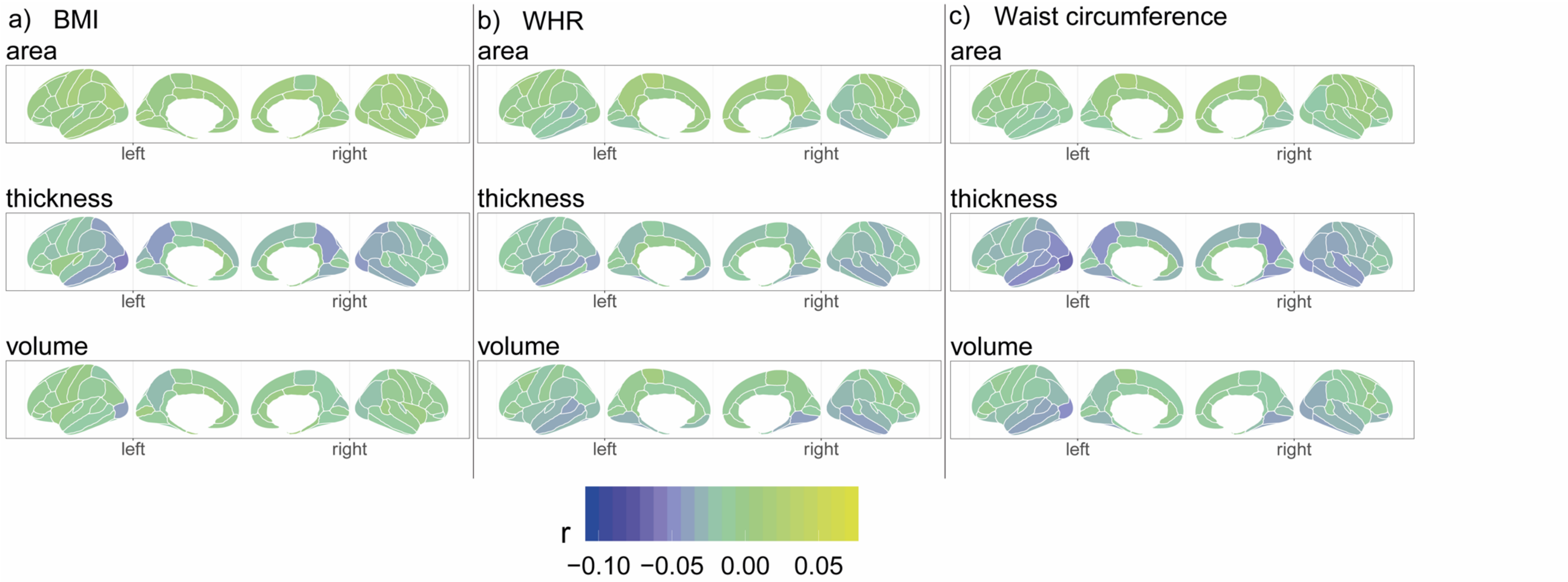

Notes: Results from model 2c that investigates body-brain connections through the inclusion of linear and quadratic terms of anthropometric measures, here showing the results of the **quadratic term** for a) BMI, b) WHR, and c) waist circumference. The regression model was adjusted for age, age^2, sex, age-by-sex, age^2-by-sex, intracranial volume (except cortical thickness), lifestyle/metabolic factors, Euler number, and site. Figure created using the *ggseg* [1] R function. *Abbreviations:* BMI – body mass index; r – partial correlation coefficient; WHR – waist-to-hip ratio.

Figure S24-a: Linear body-brain associations in healthy across models 2a/b/c (n=4,973) for anthropometric measures.

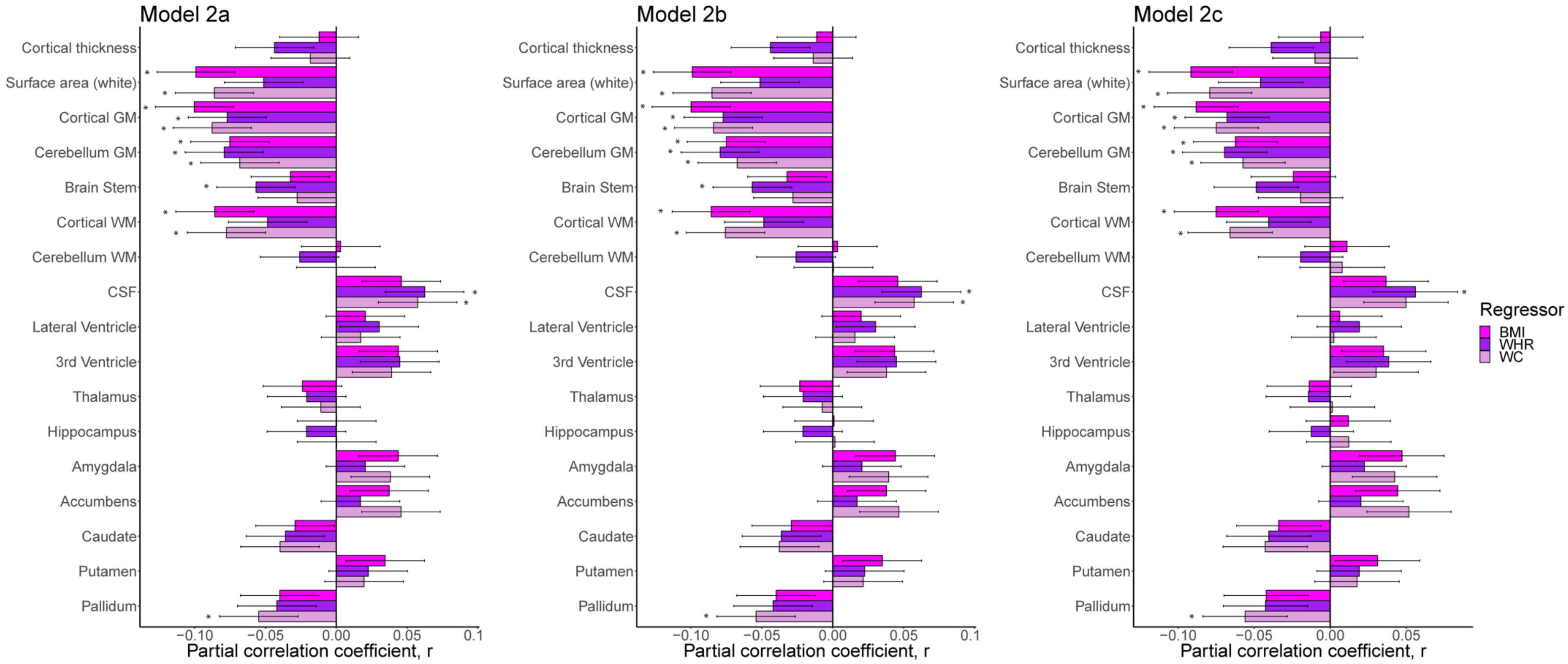

Notes: Results from regression model 2a/b/c that investigates body-brain connections through the inclusion of linear and quadratic (only model 2b/c) terms of anthropometric measures, after adjusting for respective model covariates (see manuscript). Figure displays the association between **linear term** and brain structure. *Abbreviations:* BMI – body mass index; GM- gray matter; WC – waist circumference; WHR – waist-to-hip ratio; WM – white matter.

Figure S24-b: Quadratic body-brain associations in healthy across models 2b/c (n=4,973) for anthropometric measures.

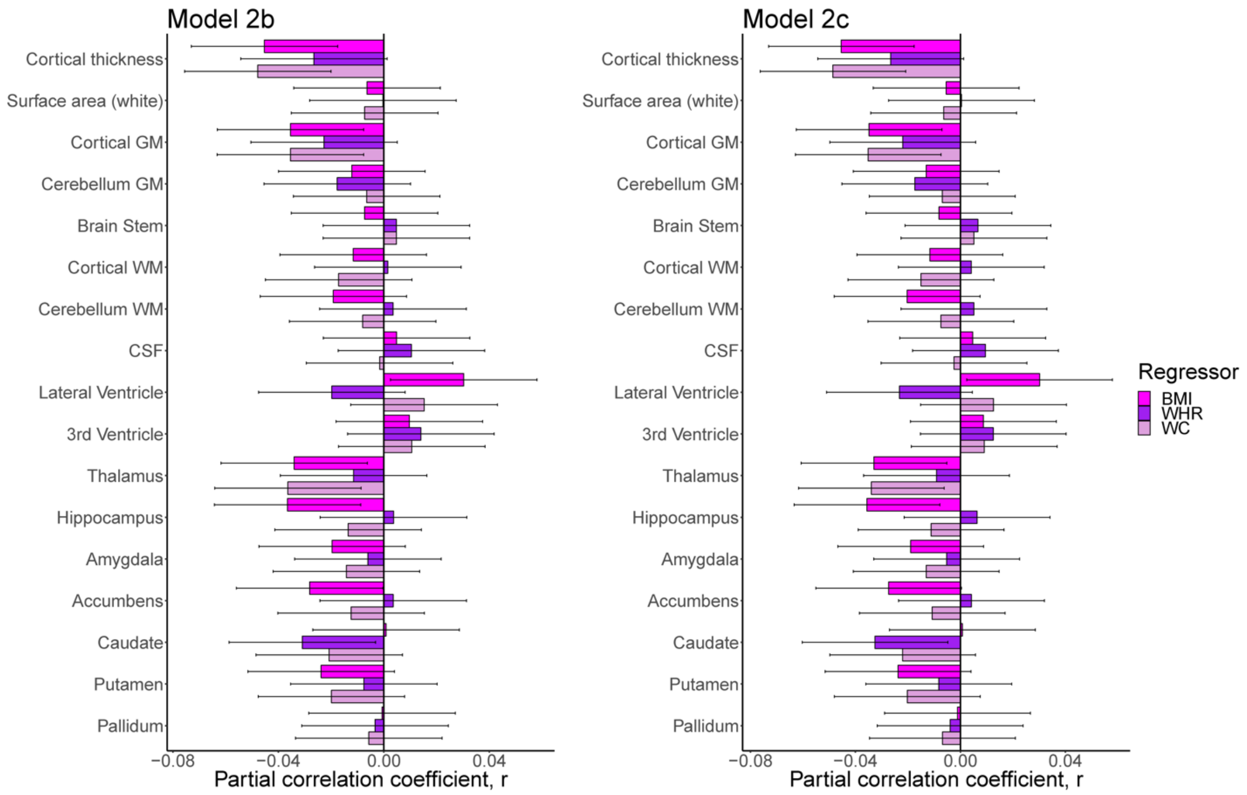

Notes: Results from regression model 2b/c that investigates body-brain connections through the inclusion of linear and quadratic terms of anthropometric measures, after adjusting for respective model covariates (see manuscript). Figure displays the association between **quadratic term** and brain structure. *Abbreviations:* BMI – body mass index; GM- gray matter; WC – waist circumference; WHR – waist-to-hip ratio; WM – white matter.

Figure S25-a: Linear body-brain associations in healthy across models 2a/b/c (n=4,973) for body composition measures.

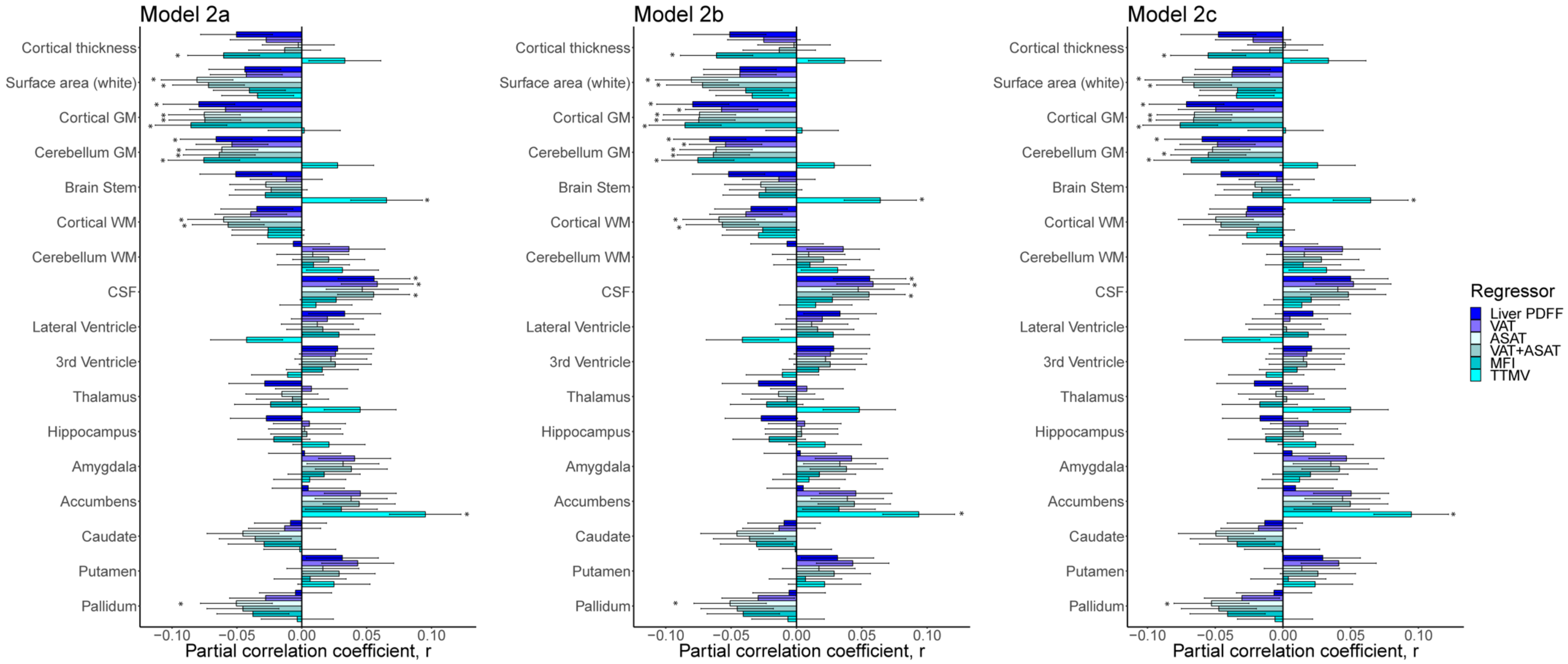

Notes: Results from regression model 2a/b/c that investigates body-brain connections through the inclusion of linear and quadratic (only model 2b/c) body composition terms, after adjusting for respective model covariates (see manuscript). Figure displays the association between **linear term** and brain structure. *Abbreviations:* ASAT – abdominal subcutaneous adipose tissue; GM- gray matter; MFI – muscle fat infiltration; PDFF – proton density fat fraction; TTMV – total thigh muscle volume; VAT – visceral adipose tissue; WM – white matter.

Figure S25-b: Quadratic body-brain associations in healthy across models 2b/c (n=4,973) for body composition measures.

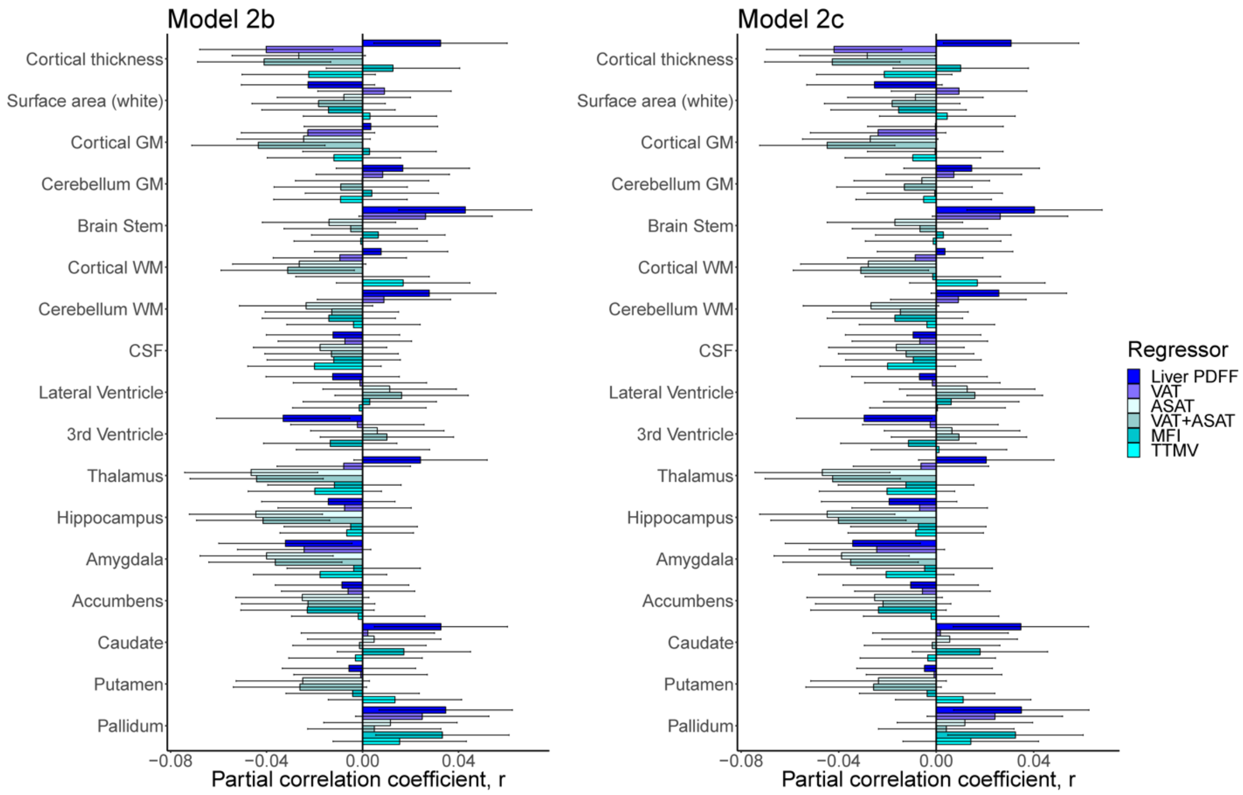

Notes: Results from regression model 2b/c that investigates body-brain connections through the inclusion of linear and quadratic body composition terms, after adjusting for respective model covariates (see manuscript). Figure displays the association between **quadratic term** and brain structure. *Abbreviations:* ASAT – abdominal subcutaneous adipose tissue; GM- gray matter; MFI – muscle fat infiltration; PDFF – proton density fat fraction; TTMV – total thigh muscle volume; VAT – visceral adipose tissue; WM – white matter.

Figure S26-a: Bilateral effects for anthropometric measures on brain structure in body MRI subsample (n=4,973) – Left hemisphere.

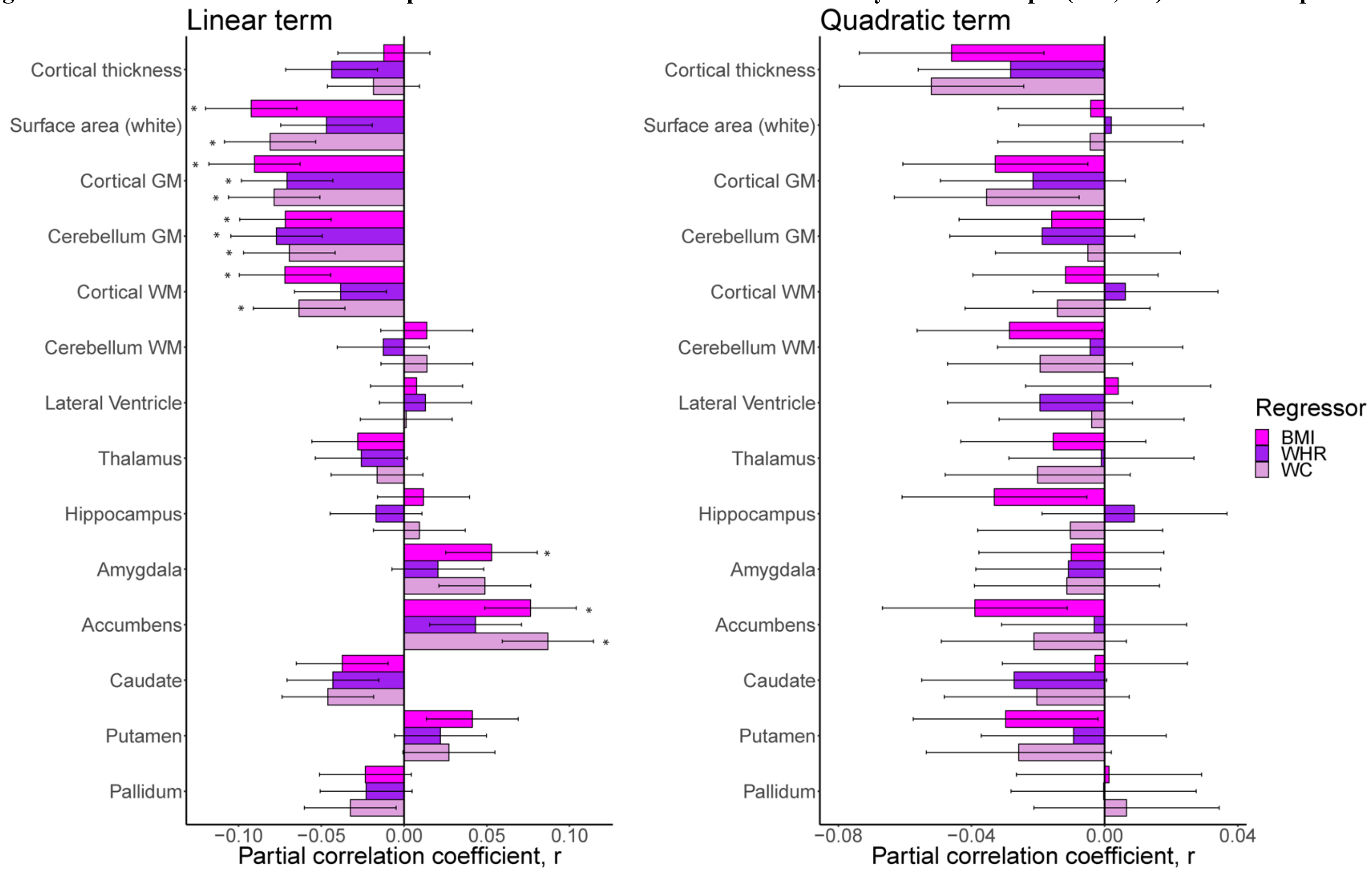

Notes: Results from regression model 2c that investigates body-brain connections through the inclusion of linear and quadratic terms of anthropometric measures, after adjusting for was adjusted for age, age^2, sex, age-by-sex, age^2-by-sex, intracranial volume (except cortical thickness), lifestyle/metabolic factors, and Euler number. Figure displays the association between **linear and quadratic terms** and brain structure for the **left hemisphere only**. *Abbreviations:* BMI – body mass index; GM- gray matter; WC – waist circumference; WHR – waist-to-hip ratio; WM – white matter.

Figure S26-b: Bilateral effects for anthropometric measures on brain structure in body MRI subsample (n=4,973) – Right hemisphere.

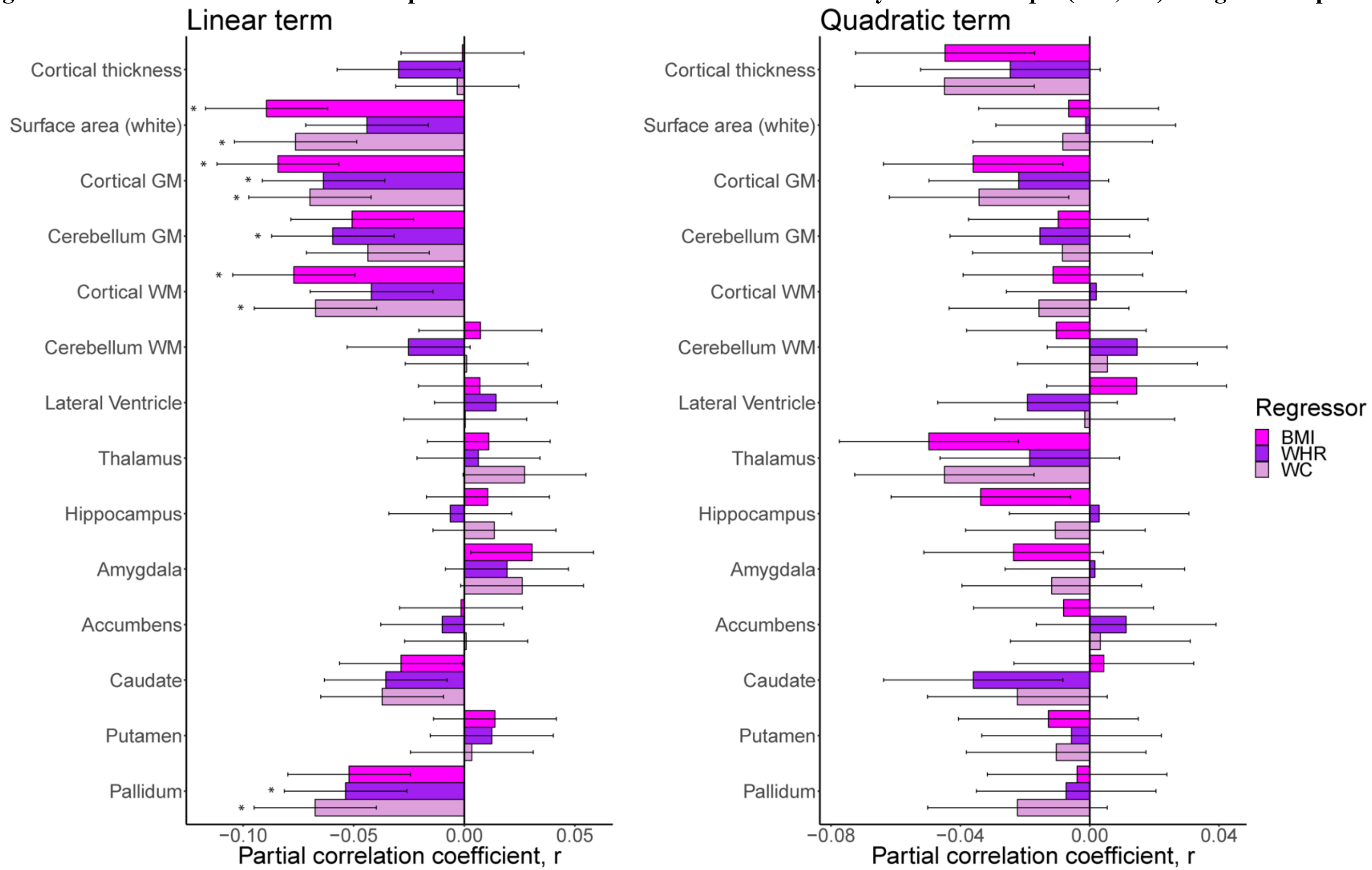

Notes: Results from regression model 2c that investigates body-brain connections through the inclusion of linear and quadratic terms of anthropometric measures, after adjusting for age, age^2, sex, age-by-sex, age^2-by-sex, intracranial volume (except cortical thickness), lifestyle/metabolic factors, and Euler number. Figure displays the association between **linear and quadratic terms** and brain structure for the **right hemisphere only**. *Abbreviations:* BMI – body mass index; GM- gray matter; WC – waist circumference; WHR – waist-to-hip ratio; WM – white matter.

Figure S27-a: Bilateral effects for body composition measures on brain structure in body MRI subsample (n=4,973) – Left hemisphere.

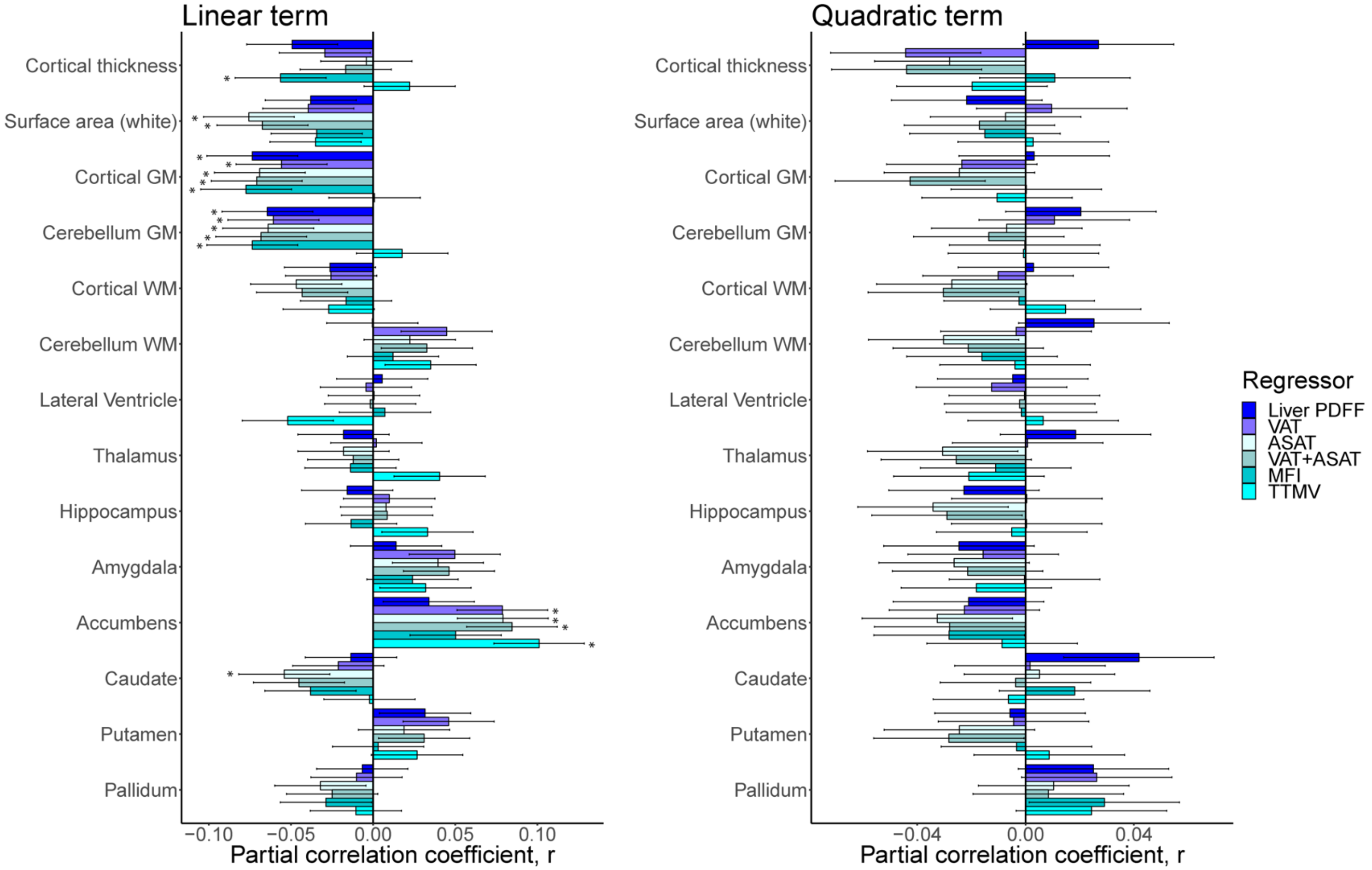

Notes: Results from regression model 2c that investigates body-brain connections through the inclusion of linear and quadratic body composition terms, after adjusting for age, age^2, sex, age-by-sex, age^2-by-sex, intracranial volume (except cortical thickness), lifestyle/metabolic factors, and Euler number. Figure displays the association between **linear and quadratic terms** and brain structure for the **left hemisphere only**. *Abbreviations:* PDFF – proton density fat fraction; VAT – visceral adipose tissue; ASAT – abdominal subcutaneous adipose tissue; VAT+ASAT – total abdominal adipose tissue; MFI – muscle fat infiltration; TTMV – total thigh muscle volume.

Figure S27-b: Bilateral effects for body composition measures on brain structure in body MRI subsample (n=4,973) – Right hemisphere.

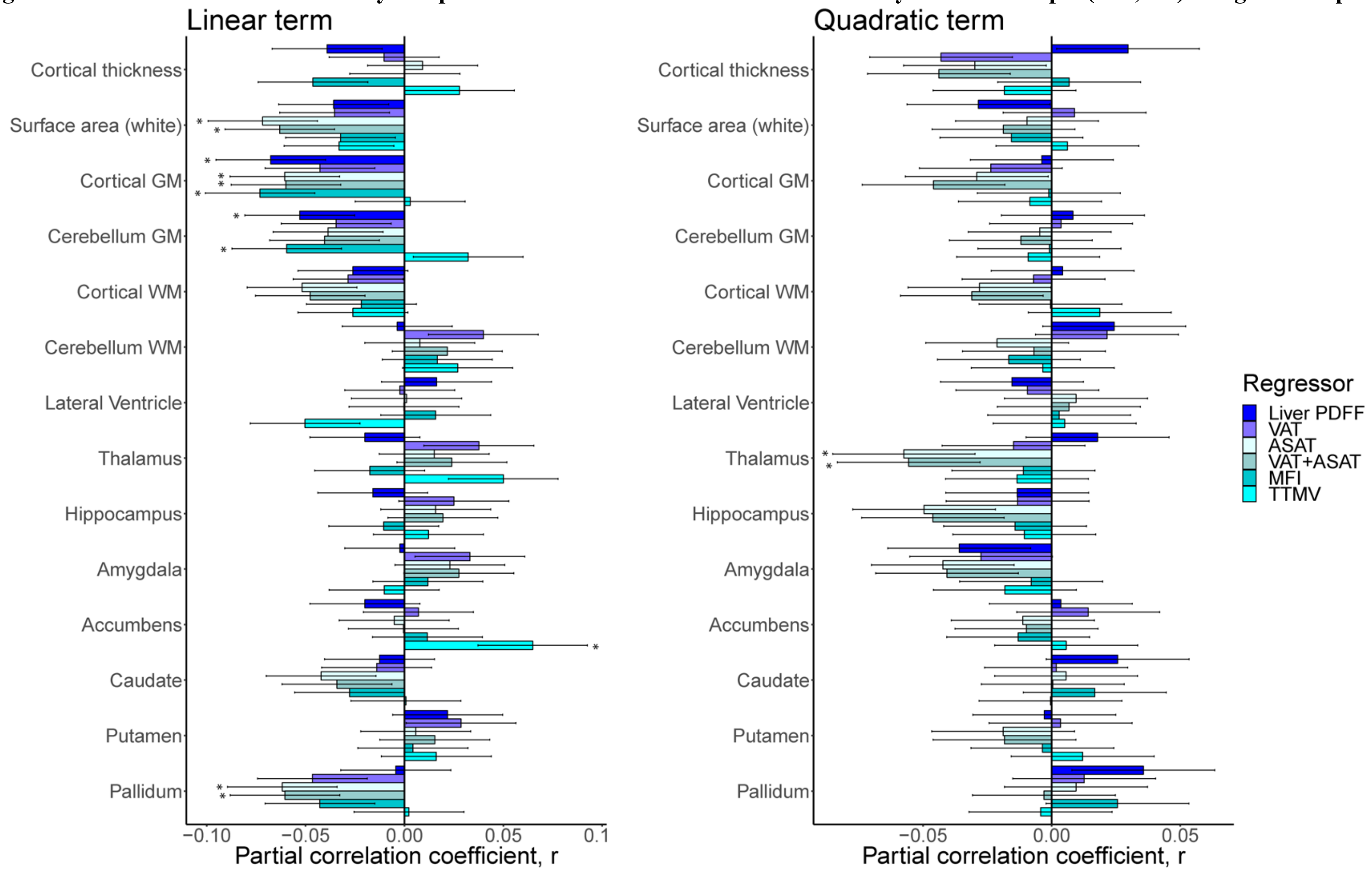

Notes: Results from regression model 2c that investigates body-brain connections through the inclusion of linear and quadratic body composition terms, after adjusting for age, age^2, sex, age-by-sex, age^2-by-sex, intracranial volume (except cortical thickness), lifestyle/metabolic factors, and Euler number. Figure displays the association between **linear and quadratic terms** and brain structure for the **right hemisphere only**. *Abbreviations:* PDFF – proton density fat fraction; VAT – visceral adipose tissue; ASAT – abdominal subcutaneous adipose tissue; VAT+ASAT – total abdominal adipose; MFI – muscle fat infiltration; TTMV – total thigh muscle volume.

Figure S28: Linear and quadratic body-brain association patterns across cortical parcellations for anthropometric measures in the body MRI subsample (n=4,973).

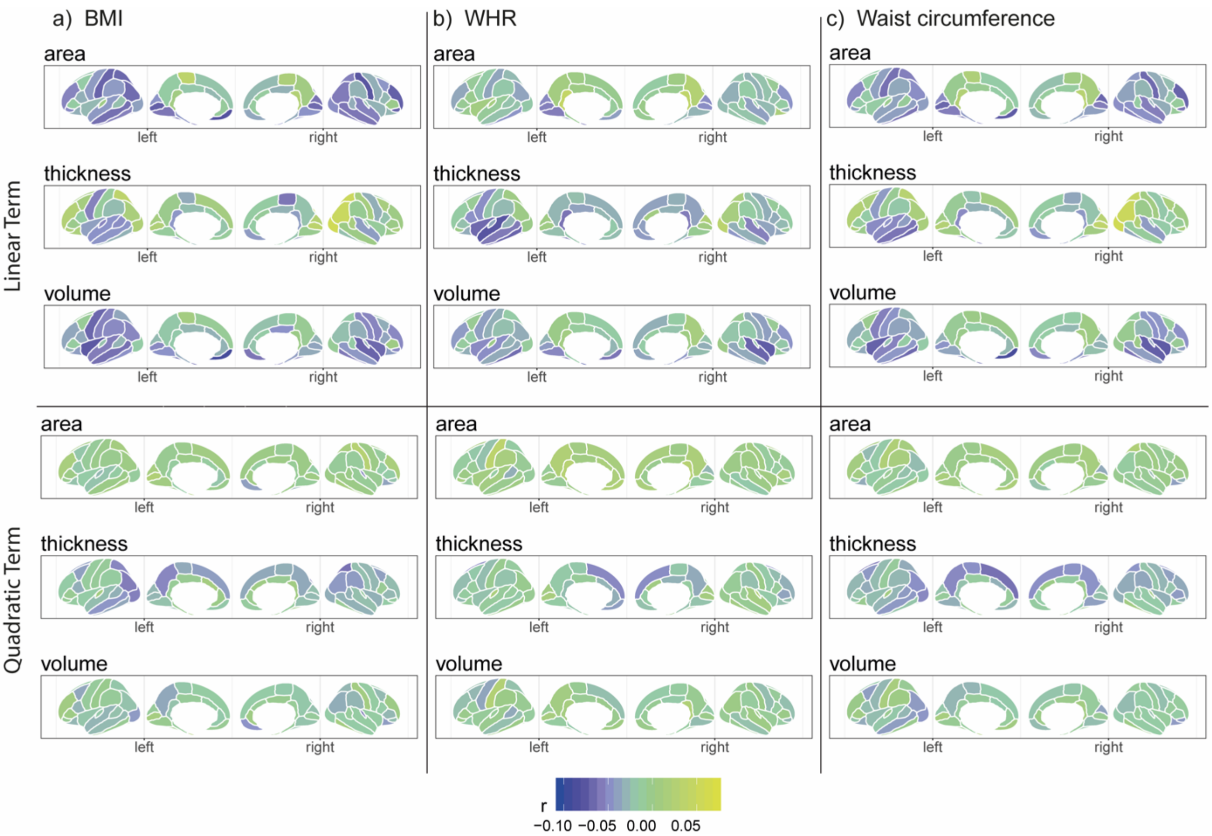

Notes: Results from model 2c that investigates body-brain connections through the inclusion of linear and quadratic terms of anthropometric measures, here showing the results of the **linear (top) and quadratic (bottom) terms** for a) BMI, b) WHR, and c) waist circumference. The regression model was adjusted for age, age^2, sex, age-by-sex, age^2-by-sex, intracranial volume (except cortical thickness), lifestyle/metabolic factors, and Euler number. Figure created using the *ggseg* [1] R function. *Abbreviations:* BMI – body mass index; WHR – waist-to-hip-ratio.

Figure S29: Quadratic body-brain association patterns across cortical parcellations for body composition measures (n=4,973).

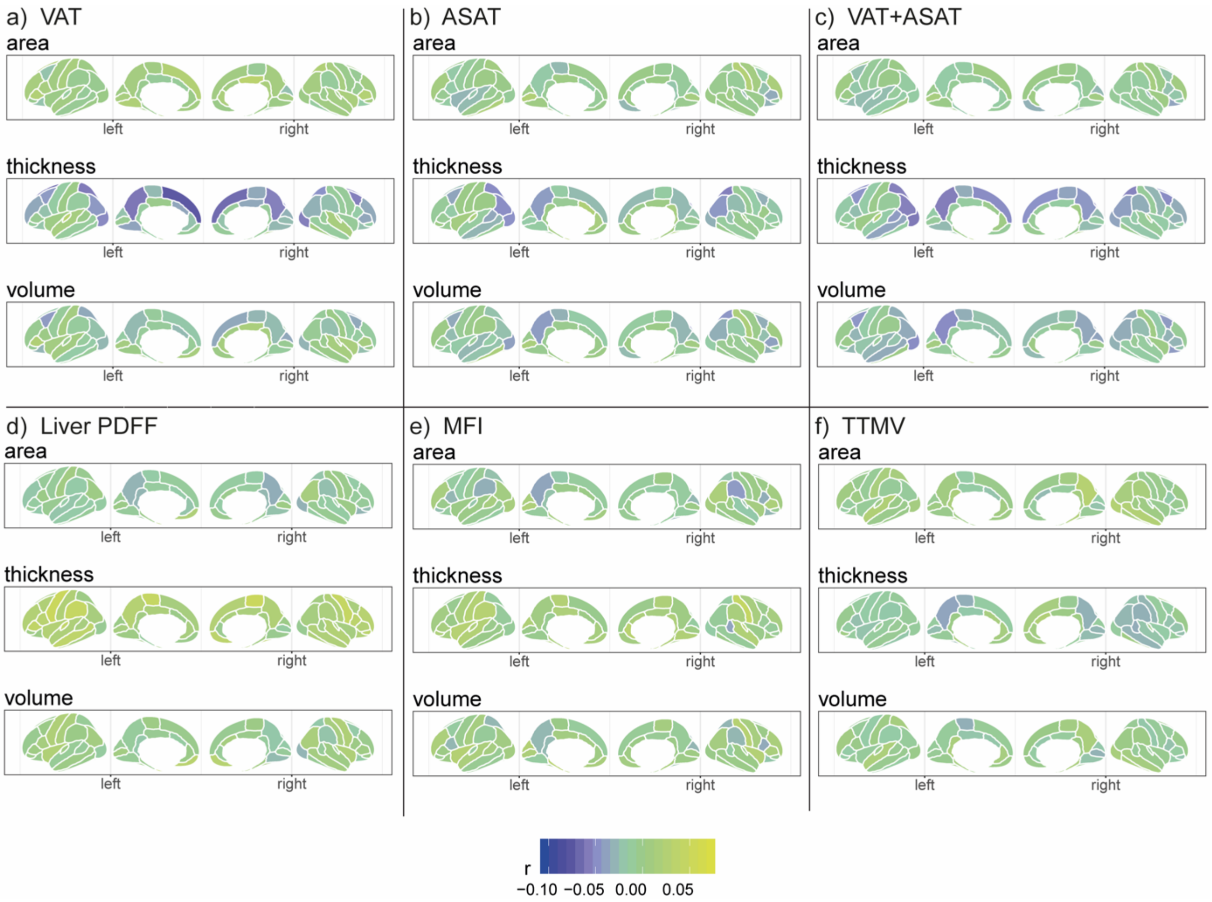

Notes: Results from model 2c that investigates body-brain connections through the inclusion of linear and quadratic body composition terms, here showing the results of the **quadratic term** for a) VAT, b) ASAT, c) VAT+ASAT, d) Liver PDFF, MFI, and TTMV. The regression model was adjusted for age, age^2, sex, age-by-sex, age^2-by-sex, intracranial volume (except cortical thickness), lifestyle/metabolic factors, and Euler number. Figure created using the *ggseg* [1] R function. *Abbreviations:* ASAT – abdominal subcutaneous adipose tissue; MFI – Muscle Fat Infiltration; PDFF – proton density fat fraction; TTMV – total thigh muscle volume; VAT – visceral adipose tissue; VAT+ASAT – total abdominal adipose tissue.

Supplemental Tables

Table S1: Demographics for the full sample (n=24,728)

	**Men** **n=11,677**	**Women** **n=13,051**	**χ^2-test / t-test / Wilcoxon rank-sum test**	**p-value**
Age (year)[1]	63±7.5	62.5±7.3	5.5	**3.1e−08**
European ancestry, N (%)[2]	11230 (96.2)	12629 (96.8)	6.3	**0.0124**
Smoker, N (%)[2,3]	471 (4)	369 (2.8)	27	**2.1e−07**
Alcohol drinker, N (%)[2,3]	11085 (94.9)	12174 (93.3)	29.7	**5.0e−08**
Height (cm)[1]	176.3±6.6	162.7±6.2	166.8	**0**
Weight (kg)[1]	83.1±13	68.3±12.7	90.2	**0**
BMI[1]	26.7±3.8	25.8±4.6	16.7	**3.2e−62**
Waist circumference (cm)[1]	93.3±10.3	82.2±11.5	80.2	**0**
Hip circumference (cm)[1]	100.3±7.1	100.4±9.6	−0.2	0.8029
WHR[1]	0.9±0.1	0.8±0.1	134.4	**0**
Diabetes, N (%)[2,3]	223 (1.9)	119 (0.9)	44.3	**2.9e−11**
Hypercholesterolemia, N (%)[2,3]	1545 (13.2)	957 (7.3)	235.1	**4.6e−53**
Hypertension, N (%)[2,3]	2422 (20.7)	1895 (14.5)	165.1	**8.6e−38**

Notes: Report mean ± standard deviation for continuous variables. P-values≤0.05 considered significant. *Abbreviations:* BMI – body mass index; WHR – waist-to-hip ratio.

[1] Welch two sample t-test.

[2] χ^2-test.

[3] Self-reported.

Table S2: Demographics for the body MRI subsample (n=4,973).

	**Men** n=2,321	**Women** n=2,652	**χ^2-test/t-test/ Wilcoxon rank-sum test**	**p-value**
Age (year)[1]	61.4±7.4	60.6±7.2	4	**7.7e−05**
European ancestry[2,3]	2222 (95.7)	2575 (97.1)	6.3	**0.0119**
Smoker[2,3]	119 (5.1)	93 (3.5)	7.6	**0.0059**
Alcohol drinker[2,3]	2202 (94.9)	2497 (94.2)	1.1	0.2964
Height (cm)[1]	175.8±6.4	162.9±6.2	72.6	**0**
Weight (kg)[1]	82.6±12.3	68.7±12.5	39.6	**3.2e−298**
BMI[4]	26.7±3.6	25.9±4.5	7.1	**1.4e−12**
Waist circumference (cm)[4]	91.9±9	81±10.7	38.9	**3.1e−289**
Hip circumference (cm)[4]	100.8±6.5	100.6±9.4	0.8	0.4425
WHR[4]	0.9±0.1	0.8±0.1	65	**0**
Liver PDFF (%)[5]	4.4±4.4	3.4±4.2	0.562	**4.0e−47**
VAT (L)[5]	4.6±2.2	2.5±1.4	1.98	**2.1e−277**
ASAT (L)[5]	5.6±2.3	7.8±3.3	−2.0091	**1.7e−151**
VAT+ASAT (L)[4]	10.2±4.1	10.3±4.5	−1	0.3231
MFI (%)[4]	6.6±1.5	7.7±1.7	−25.4	**2.2e−134**
TTMV (L)[4]	12.6±1.8	8.4±1.2	97.2	**0**
Diabetes[2,3]	38 (1.6)	21 (0.8)	6.8	**0.0089**
Hypercholesterolemia[2,3]	291 (12.5)	175 (6.6)	50.7	**1.1e−12**
Hypertension[2,3]	590 (25.4)	469 (17.7)	43.7	**3.8e−11**

Notes: Report mean ± standard deviation for continuous variables, number of participants (percentage) for categorical variables. P-values≤0.05 considered significant. *Abbreviations:* ASAT – abdominal subcutaneous adipose tissue; BMI – body mass index; L – liter; PDFF – proton density fat fraction; VAT – visceral adipose tissue; WHR – waist-to-hip ratio.

[1] Two-sample t-test

[2] χ^2-test used.

[3] Self-reported.

[4] Welch two sample t-test.

[5] Wilcoxon rank-sum test.

Tables S2-S31 are presented sheet-wise in a separate supplemental excel document

The included tables are:

Table S3: Sample description – Age- and sex-related associations on anthropometrics (full sample).

Table S4: Sample description – Age- and sex-related associations on anthropometrics and body composition (body MRI subsample).

Table S5: Sample description – Age- and sex-related associations on brain structure (full sample).

Table S6: Body-brain associations for BMI as covariate of interests (full sample)

Table S7: Body-brain associations for WHR as covariate of interests (full sample)

Table S8: Body-brain associations for waist circumference as covariate of interests (full sample)

Table S9: Body-brain associations for BMI, WHR, and waist circumference as covariate of interests for bilateral measures of brain structures (full sample).

Table S10: Body-brain associations for cortical parcellations with BMI as covariate of interests (full sample)

Table S11: Body-brain associations for cortical parcellations with WHR as covariate of interests (full sample)

Table S12: Body-brain associations for cortical parcellations with waist circumference as covariate of interests (full sample)

Table S13: Body-brain associations for BMI as covariate of interests (body MRI subsample)

Table S14: Body-brain associations for WHR as covariate of interests (body MRI subsample)

Table S15: Body-brain associations for waist circumference as covariate of interests (body MRI subsample)

Table S16: Body-brain associations for VAT (visceral adipose tissue) as covariate of interests (body MRI subsample)

Table S17: Body-brain associations for ASAT (abdominal subcutaneous adipose tissue) as covariate of interests (body MRI subsample)

Table S18: Body-brain associations for VAT+ASAT (total abdominal adipose tissue) as covariate of interests (body MRI subsample)

Table S19: Body-brain associations for liver PDFF (proton density fat fraction) as covariate of interests (body MRI subsample)

Table S20: Body-brain associations for MFI (muscle fat infiltration) as covariate of interests (body MRI subsample)

Table S21: Body-brain associations for TTMV (total thigh muscle volume) as covariate of interests (body MRI subsample)

Table S22: Body-brain associations for BMI, WHR, waist circumference, VAT, ASAT, VAT+ASAT, liver PDFF, MFI and TTMV as covariate of interests for bilateral measures of brain structures (subsample).

Table S23: Body-brain associations for cortical parcellations with BMI as covariate of interests (body MRI subsample)

Table S24: Body-brain associations for cortical parcellations with WHR as covariate of interests (body MRI subsample)

Table S25: Body-brain associations for cortical parcellations with waist circumference as covariate of interests (body MRI subsample)

Table S26: Body-brain associations for cortical parcellations with VAT (visceral adipose tissue) as covariate of interests (body MRI subsample)

Table S27: Body-brain associations for cortical parcellations with ASAT (abdominal subcutaneous adipose tissue) as covariate of interests (body MRI subsample)

Table S28: Body-brain associations for cortical parcellations with VAT+ASAT (total abdominal adipose tissue) as covariate of interests (body MRI subsample)

Table S29: Body-brain associations for cortical parcellations with liver PDFF (proton density fat fraction) as covariate of interests (body MRI subsample)

Table S30: Body-brain associations for cortical parcellations with MFI (muscle fat infiltration) as covariate of interests (body MRI subsample)

Table S31: Body-brain associations for cortical parcellations with TTMV (total thigh muscle volume) as covariate of interests (body MRI subsample)

Supplemental Notes

Note S1: Exclusion criteria for the study

All participants with cancer diagnosis were excluded (http://biobank.ctsu.ox.ac.uk/showcase/; UK-biobank data-field ID 20001). We also excluded participants that were diagnosed with selected traumas, neurological, psychiatric, substance abuse, cardiovascular, liver, or severe infectious conditions. Table SN1 yields an overview of the excluded non-cancer diagnosis (UK-biobank data-field 20002). We based the exclusion criteria on self-reported diagnosis.

Table SN1: Excluded non-cancer diagnosis.

Code	Meaning	Code	Meaning	Code	Meaning
**1066**	heart/cardiac problem	**1592**	aortic dissection	**1261**	multiple sclerosis (MS)
**1075**	heart attack/myocardial infarction	**1493**	other venous/lymphatic disease	**1262**	parkinsons disease
**1076**	heart failure/pulmonary odema	**1494**	varicose veins	**1263**	dementia/alzheimers/cognitive impairment
**1077**	heart arrhythmia	**1495**	lymphoedema	**1397**	other demyelinating disease (not MS)
**1471**	atrial fibrillation	**1593**	varicose ulcer	**1264**	Epilepsy
**1483**	atrial flutter	**1136**	liver/biliary/pancreas problem	**1265**	migraine
**1484**	wolff parkinson white/wpw syndrome	**1155**	hepatitis	**1433**	cerebral palsy
**1485**	irregular heart beat	**1156**	infective/viral hepatitis	**1434**	other neurological problem
**1486**	sick sinus syndrome	**1157**	non-infective hepatitis	**1436**	headaches (not migraine)
**1487**	svt/supraventricular tachycardia	**1578**	hepatitis a	**1437**	myasthenia gravis
**1488**	mitral valve prolapse	**1579**	hepatitis b	**1525**	benign/essential tremor
**1489**	mitral stenosis	**1580**	hepatitis c	**1526**	polio/poliomyelitis
**1584**	mitral valve disease	**1581**	hepatitis d	**1659**	meningioma/benign meningeal tumour
**1585**	mitral regurgitation/incompetence	**1582**	hepatitis e	**1683**	benign neuroma
**1078**	heart valve problem/heart murmur	**1158**	liver failure/cirrhosis	**1240**	neurological injury/trauma
**1586**	aortic valve disease	**1506**	primary biliary cirrhosis	**1266**	head injury
**1587**	aortic regurgitation/incompetence	**1604**	alcoholic liver disease/alcoholic cirrhosis	**1267**	spinal injury
**1490**	aortic stenosis	**1507**	haemochromatosis	**1297**	muscle/soft tissue problem
**1079**	cardiomyopathy	**1508**	jaundice (unknown cause)	**1407**	Burns
**1588**	hypertrophic cardiomyopathy (hcm/hocm)	**1475**	sclerosing cholangitis	**1626**	fracture skull/head
**1589**	pericarditis	**1244**	infection of nervous system	**1630**	fracture neck/cervical fracture
**1590**	pericardial effusion	**1245**	brain abscess/intracranial abscess	**1625**	cellulitis
**1080**	pericardial problem	**1246**	encephalitis	**1350**	polycystic ovaries/ovarian syndrome
**1426**	myocarditis	**1247**	meningitis	**1371**	sarcoidosis

**1479**	rheumatic fever	**1248**	spinal abscess	**1439**	hiv/aids
**1081**	stroke	**1249**	cranial nerve problem/palsy	**1577**	typhoid fever
**1086**	subarachnoid haemorrhage	**1250**	bell's palsy/facial nerve palsy	**1443**	schistosomiasis/bilharzia
**1491**	brain haemorrhage	**1523**	trigemminal neuralgia	**1288**	nervous breakdown
**1583**	ischaemic stroke	**1251**	spinal cord disorder	**1289**	schizophrenia
**1231**	post-natal depression	**1408**	alcohol dependency	**1409**	opioid dependency
**1082**	transient ischaemic attack (tia)	**1252**	paraplegia	**1290**	deliberate self-harm/suicide attempt
**1083**	subdural haemorrhage/haematoma	**1524**	spina bifida	**1291**	mania/bipolar disorder/manic depression
**1425**	cerebral aneurysm	**1254**	peripheral nerve disorder	**1531**	post-natal depression
**1067**	peripheral vascular disease	**1256**	acute infective polyneuritis/guillain-barre syndrome	**1469**	post-traumatic stress disorder
**1068**	venous thromboembolic disease	**1257**	trapped nerve/compressed nerve	**1470**	anorexia/bulimia/other eating disorder
**1087**	leg claudication/intermittent claudication	**1468**	diabetic neuropathy/ulcers	**1615**	Obsessive compulsive disorder (ocd)
**1088**	arterial embolism	**1258**	chronic/degenerative neurological problem	**1243**	psychological/psychiatric problem
**1093**	pulmonary embolism ± dvt	**1259**	motor neurone disease	**1286**	Depression
**1094**	deep venous thrombosis (dvt)	**1260**	myasthenia gravis	**1287**	anxiety/panic attacks
**1591**	aortic aneurysm rupture	**1255**	peripheral neuropathy	**1288**	nervous breakdown
**1410**	other substance abuse/dependency	**1614**	stress	**1616**	insomnia

Note S2: Extracted/computed demographic and clinical variables

For an overview of the extracted demographic and clinical data-field IDs from the UK-biobank, see Table SN2. Additionally, we computed the following ratios: (1) Body-mass-index (BMI) from weight and standing height as: weight in kg/(height in meters)^2, and (2) waist-to-hip ratio computed as: waist circumference/hip circumference.

We created binary (yes/no) variables respectively for: diagnosis (data-field 20002.2.*) of diabetes (diabetes, diabetes type 1, diabetes type2), hypertension, and high cholesterol (hypercholesterolemia); current alcohol consumption, and current cigarette smoking (yes: current, no: previous/never). For ethnicity, we created a binary variable for self-identified European/non-European ancestry based on the MRI time point when available, and complemented incomplete data with baseline information since ethnicity does not change with time (although knowledge/perception of ethnic background can change).

Table SN2: Extracted demographic and clinical variables with data-field ID.

Data-field ID	Field Description
31.0.0	Sex
54.2.0	Assessment center
21003.2.0	Age
50.2.0	Standing height
21002.2.0	Weight
48.2.0	Waist circumference
49.2.0	Hip circumference
21000.*.0[1]	Ethnicity
20001.2.*[2]	Cancer diagnosis (self-reported)
20002.2.*[3]	Non-cancer diagnosis (self-reported)
20117.2.0	Alcohol drinking status
20116.2.0	Cigarette smoking status

[1] Baseline and imaging timepoint extracted.
[2] All sub-items (*), from 0 to 5 extracted, imaging timepoint used when available.
[3] All sub-items (*), from 0 to 32 extracted.

Note S3: MRI acquisition

Brain MRI was available from three sites (Cheadle, Reading, and Newcastle), and body and liver MRI from one site (Cheadle). Similar scanners/protocols were used across sites [2,3]. Briefly, a single sagittal T1-weighted brain MRI was acquired on a 3T Siemens Skyra scanner equipped with a 32-channel head coil using a 3D MPRAGE sequence with pre-scan normalization [2,3]. Body and liver MRI was acquired on a 1.5T Siemens MAGNETOM Aera scanner using a body dual-echo Dixon Vibe protocol [4,5] and a single-slice multi-echo gradient Dixon acquisition [5], respectively.

Note S4: Body MRI processing details

For the body MRI data, we acquired the processed data from the UK-biobank. The data was processed for abdominal fat, liver proton density fat fraction (PDFF), muscle fat infiltration, and thigh muscle volume by AMRA (Linköping, Sweden; https://www.amramedical.com).

The body MRI processing by AMRA include intensity inhomogeneity correction, non-rigid registration of atlases to acquired image volumes, quantification of fat and muscle composition using a voting scheme, and visual inspection for segmentation accuracy and manual adjustment [4] (for technical details, see [6–8]). Adipose tissue within the abdominal cavity was defined as visceral adipose tissue (VAT), adipose tissue between the top of the femoral head and the top of T9 was defined as abdominal subcutaneous adipose tissue (ASAT), and lean thigh muscle volume included the gluteus, iliacus, adductors, hamstrings, quadriceps femoris and sartorius [4]. Muscle fat infiltration (MFI) was derived from the anterior thighs [5]. The liver MRI data was processed using a magnitude-based chemical shift technique with a 6-peak lipid model, and then registered to the body MRI data and corrected for liver T2* to obtain a T1-weighted measure of liver proton density fat fraction (PDFF) of the whole liver [5] (for further details see Linge et al. 2018, supplemental material [5]). AMRA implements manual quality control of the image/segmentation quality.

We extracted volumetric body MRI variables from the UK-biobank repository (Table SN3). Of the participants with body MRI that met the inclusion criteria of this study, 32.9% were missing the AMRA provided Total trunk fat (VAT+ASAT; Data-field ID: 22410.2.0). We therefore chose to recompute this variable as the sum of VAT (Data-field ID: 22407.2.0) and ASAT (Data-field ID: 22408.2.0), which is yields the same number as the one released by AMRA.

For further information about the UK-biobank body MRI, see the documentation at the UK-biobank showcase (http://biobank.ctsu.ox.ac.uk/showcase/).

Table SN3: Extracted body MRI variables with data-field ID

Data-field ID	Field Description
12224.2.0	Indications whether abdominal MRI has been completed.
22407.2.0	Visceral adipose tissue (VAT) volume
22408.2.0	Abdominal subcutaneous adipose tissue (ASAT) volume
22409.2.0	Total thigh muscle volume (TTMV; sum of all lean thigh muscle volumes)
22410.2.0	Total trunk fat (VAT+ASAT)
22435.2.0	Muscle fat infiltration (MFI)
22436.2.0	Liver proton density fat fraction (PDFF)
22414.2.*[1]	Image quality indicator

[1]All sub-items (*) from 0 to 1 extracted. Variable used for quality control of body MRI measures.

Note S5: Brain MRI Quality control

For the T1 weighted brain MRI data, we applied an automated quality control based on the FreeSurfer [9] Euler number [10,11]. Higher negative Euler number imply worse image quality. For each hemisphere, participants were iteratively excluded if the Euler number exceeded three standard deviations (SD) from the mean in either hemisphere (one-sided). We iterated until there were no outliers left, resulting in seven iterations (Figure S1).

Table SN4 gives the number of excluded/included participants together with the average Euler number, while Figure SN1 shows illustrates the Euler number distribution of included/excluded participants for the left/right hemisphere.

Table SN4: Average FreeSurfer Euler number of excluded/included participants.

	**Included (n=24,728)**	**Excluded (n=2,602)**
Left hemisphere	-51.4±23.7	-174.1±87.8
Right hemisphere	-48.7±22.6	-164±84.2

Notes: Report mean ± standard deviation

Figure SN1: Violin plots of included/excluded participants based FreeSurfer Euler numbers.

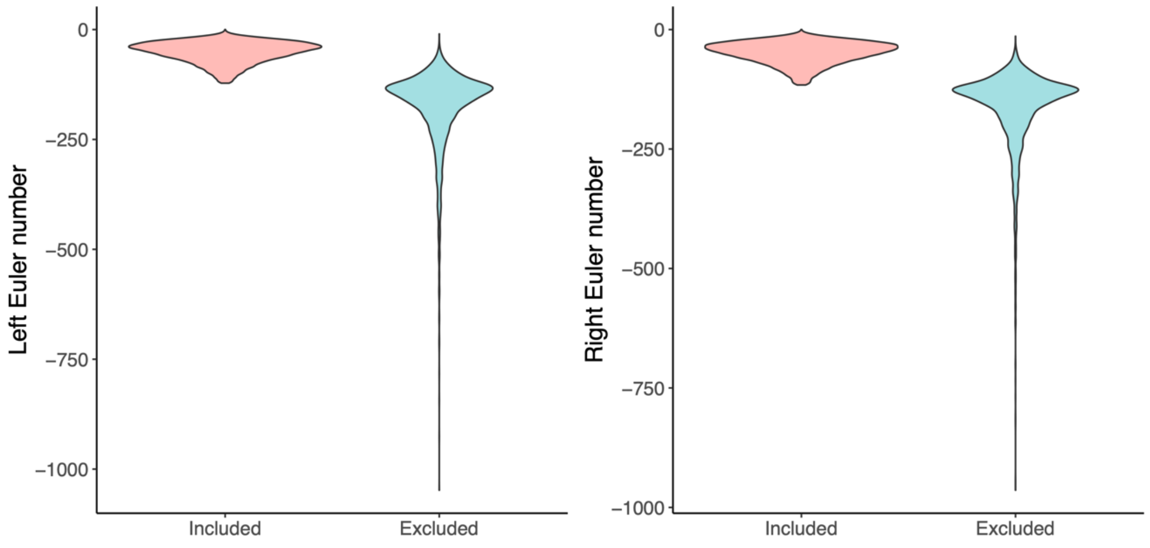

Notes: Plots of included/excluded participants are not adjusted for sample size.

Note S6: Linear regression models using the *lm* in r

Below we present the linear regression models as implemented in R (version 3.5.2; https://www.r-project.org) using the *lm* for the sample description and main analyses.

Sample description: Anthropometrics and body composition

Model 1a: lm(log(body†) ~ poly(Age,2)+Sex**)**

Model 1b: lm(log(body†) ~ poly(Age,2)*Sex**)**

Model 1c: lm(log(body†) ~ poly(Age,2)*Sex + Ethnicity + Diabetic + Hypercholesteremia + Hypertension + Smoking + Alcohol**)**

Sample description: Brain structure

Model 1a: lm(brain‡ ~ poly(Age,2)+Sex + ICV§ + Euler number + Assessment center**)**

Model 1b: lm(brain‡ ~ poly(Age,2)*Sex + ICV§ + Euler number + Assessment center**)**

Brain structure, anthropometrics and body composition

Model 2a: lm(*brain‡ ~ body† + poly(Age,2)*Sex + ICV§ + Euler number + Assessment center*****)***

Model 2b: lm(*brain‡ ~ poly(body†,2) + poly(Age,2)*Sex + ICV§ + Euler number + Assessment center*****)***

Model 2c: lm(*brain‡ ~ poly(body†,2) + poly(Age,2)*Sex + ICV§ + Ethnicity + Diabetic + Hypercholesteremia+ Hypertension + Smoking + Alcohol + Euler number + Assessment center*****)***

† Anthropometric or body composition measure.
‡ Measured brain structure. We *log*-transformed CSF, Lateral and 3rd ventricle.
§ We did not adjust the mean cortical thickness for ICV (intracranial volume).
** Not included for body MRI subset (single site).

Note S7: Sample description analyses of anthropometric and body composition measures and brain structure.

Sample description: Anthropometrics and body composition

Analyses including the full sample (Figure SN2; Table S2) revealed, as expected, higher BMI, WHR, and waist circumference in men compared to women, and that age was negatively associated with BMI and positively associated with WHR and to a certain degree with waist circumference. In the body MRI subsample (Figure SN3; Table S3), men showed higher liver fat, visceral adipose tissue, and total thigh muscle volume, and lower abdominal subcutaneous adipose tissue and muscle fat infiltration compared to women. Age was positively associated with liver fat, visceral adipose tissue, and muscle fat infiltration, and negatively with total thigh muscle volume. Liver fat and visceral adipose tissue showed significant age-by-sex interactions suggestive of age-related attenuation in men. These effects were similar across models 1a/b/c.

Sample description: Brain structure

Analyses including the full sample revealed significant age- and sex-associations across most included brain structures. As expected, global cortical and cerebellum measures and subcortical structures generally showed age-related decreases with significant quadratic terms, indicating increasing age-related associations at higher ages, while CSF, lateral, and third ventricles showed similar increases with age. Pallidum was positively associated with age. Men generally showed higher brain volumes and steeper age-related decreases, except thinner mean cortical thickness, and lower total cerebellum white matter, relative to women. These effects were similar across models 1a/b (Figure SN4; Table S4).

Figure SN2: Age and sex in relation to anthropometric measures (n=24,728).

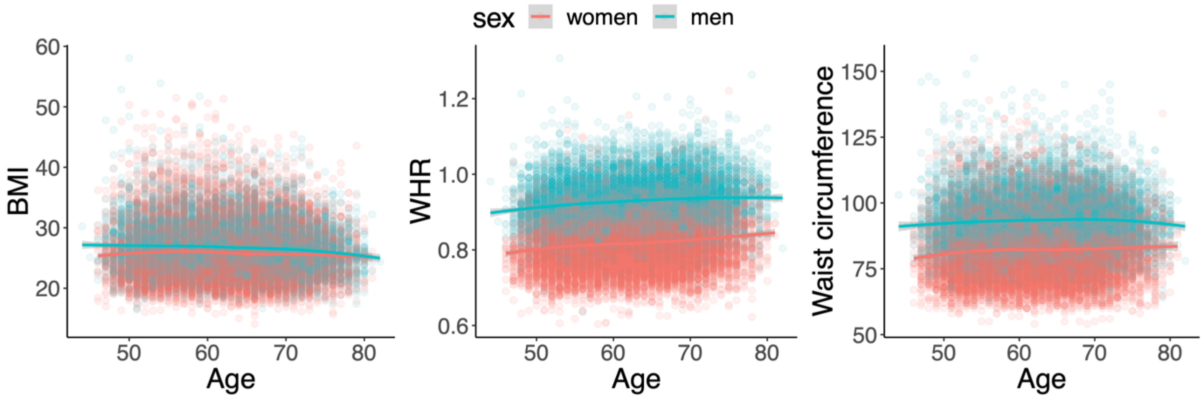

Notes: Unadjusted LOESS scatter plots. Age is given in years and waist circumference is given in cm. BMI is computed as weight in kg / (height in meters)^2 and WHR is computed as waist circumference in cm / hip circumference in cm. *Abbreviations:* BMI – Body mass index; WHR – waist-hip-ratio.

Figure SN3: Age and sex in relation to anthropometric and body composition measures (n=4,973).

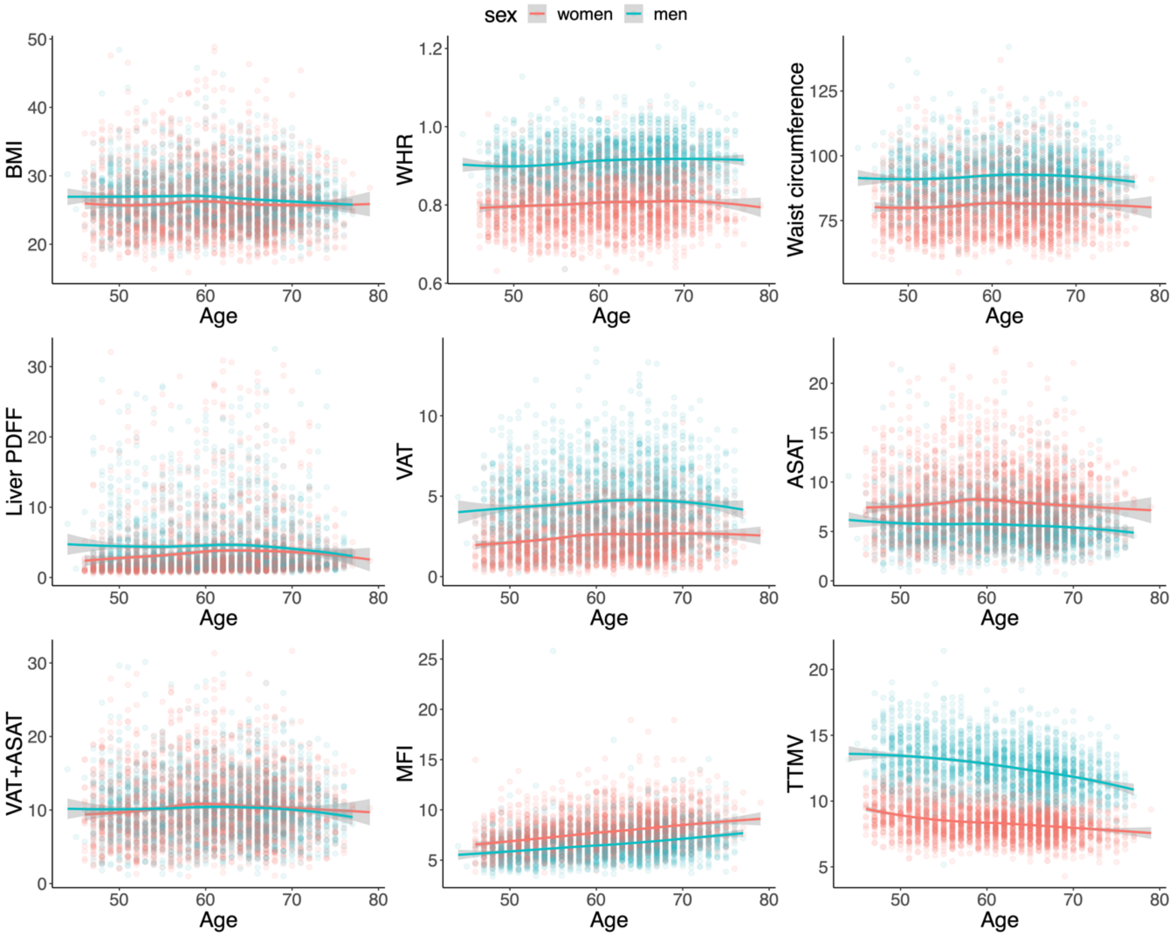

Notes: Unadjusted LOESS scatter plots. Age is given in years and waist circumference is given in cm. BMI is computed as weight in kg / (height in meters)^2 and WHR is computed as waist circumference in cm / hip circumference in cm. VAT, ASAT, VAT+ASAT, and TTMV are given in L, while Liver PDFF and MFI are given in %. *Abbreviations:* ASAT – abdominal subcutaneous adipose tissue; BMI – Body mass index; L – liter; MFI – muscle fat infiltration; PDFF – proton density fat fraction; TTMV – total thigh muscle volume; VAT – visceral adipose tissue; VAT+ASAT – total abdominal adipose tissue; WHR – waist-hip-ratio.

Figure SN4: Age and sex in relation to brain structure in generally healthy participants (n=24,728).

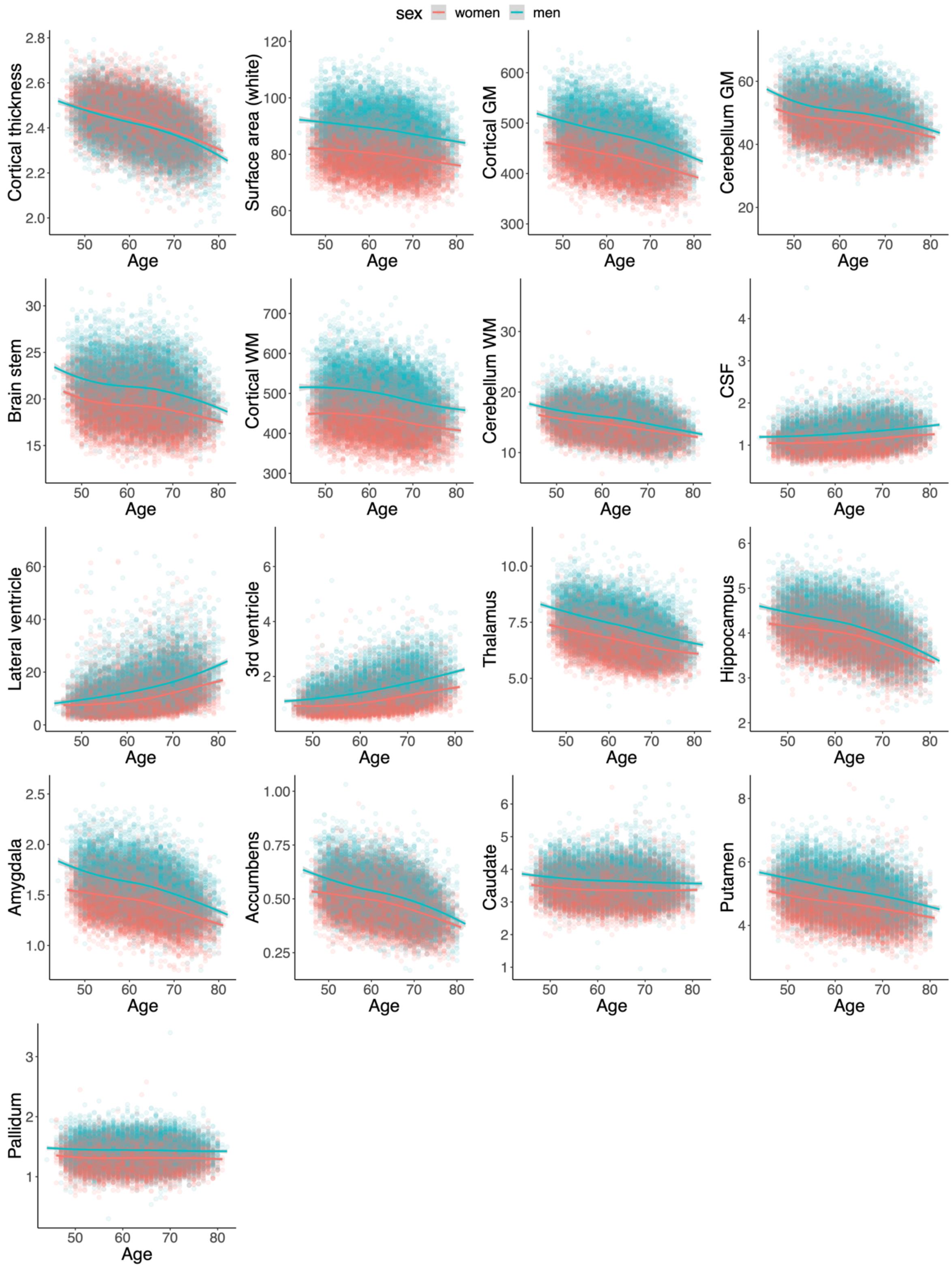

Notes: Unadjusted LOESS scatter plots. Age is given in years and all brain structures are given in ml (except surface area given in m^2 and cortical thickness given in mm). *Abbreviations:* CSF – cerebrospinal fluid; GM – gray matter; ICV – intracranial volume; WM – white matter.

References

1 Mowinckel AM, Vidal-Piñeiro D. Visualization of Brain Statistics With R Packages ggseg and ggseg3d. *Advances in Methods and Practices in Psychological Science* 2020; **3**: 466–483.

2 Alfaro-Almagro F *et al.* Image processing and Quality Control for the first 10,000 brain imaging datasets from UK Biobank. *Neuroimage* 2018; **166**: 400–424.

3 Miller KL *et al.* Multimodal population brain imaging in the UK Biobank prospective epidemiological study. *Nature Neuroscience* 2016; **19**: 1523–1536.

4 West J *et al.* Feasibility of MR-Based Body Composition Analysis in Large Scale Population Studies. *PLoS ONE* 2016; **11**: e0163332.

5 Linge J *et al.* Body Composition Profiling in the UK Biobank Imaging Study. *Obesity* 2018; **26**: 1785–1795.

6 Borga M *et al.* Validation of a fast method for quantification of intra-abdominal and subcutaneous adipose tissue for large-scale human studies. *NMR in Biomedicine* 2015; **28**: 1747–1753.

7 Karlsson A *et al.* Automatic and quantitative assessment of regional muscle volume by multi-atlas segmentation using whole-body water–fat MRI. *Journal of Magnetic Resonance Imaging* 2015; **41**: 1558–1569.

8 Leinhard OD *et al.* Quantitative abdominal fat estimation using MRI. 2008, pp 1–4.

9 Fischl B. FreeSurfer. *NeuroImage* 2012; **62**: 774–81.

10 Dale AM, Fischl B, Sereno MI. Cortical Surface-Based Analysis: I. Segmentation and Surface Reconstruction. *NeuroImage* 1999; **9**: 179–194.

11 Rosen AFG *et al.* Quantitative assessment of structural image quality. *Neuroimage* 2018; **169**: 407–418.
